# Successful rapid improvement of acute respiratory distress syndrome induced by EGFR-mutated non-small cell lung cancer with almonertinib: a case report

**DOI:** 10.1186/s12890-024-03292-3

**Published:** 2024-09-27

**Authors:** Cheng Sun, Zhike Liang, Zhiyun Yan, Yawen Feng, Wanna Tang, Shuquan Wei, Weinong Zhong, Ziwen Zhao, Yujun Li

**Affiliations:** 1 Department of Pulmonary and Critical Care Medicine, Guangzhou First People’s Hospital, South China University of Technology, Guangzhou, Guangdong 510180 China; 2 Jinan University, Guangzhou, Guangdong 510632 China; 3 Guangdong Medical University, Zhanjiang, Guangdong 510089 China; 4 School of Pharmacy, Guangdong Pharmaceutical University, Guangzhou, Guangdong 510006 China

**Keywords:** Acute respiratory distress syndrome, Non-small cell lung cancer, Epidermal growth factor receptor, Almonertinib, Tyrosine kinase inhibitor, Case report

## Abstract

**Background:**

Acute respiratory distress syndrome (ARDS) is a life-threatening condition frequently encountered in critically ill patients, including those with advanced non-small cell lung cancer (NSCLC). Almonertinib, a third-generation epidermal growth factor receptor (EGFR) tyrosine kinase inhibitor (TKI), has shown promise as a first-line treatment for NSCLC with classical EGFR mutations. However, its efficacy in NSCLC patients suffering from ARDS has not been well-documented.

**Case Presentation:**

We report the case of a 63-year-old Chinese Han female with severe NSCLC complicated by ARDS. Upon hospital admission, the patient exhibited progressive dyspnea and required intubation to maintain oxygenation. Pathological analysis of bronchoalveolar lavage fluid sediment confirmed lung adenocarcinoma, and genetic testing of blood identified an EGFR E19 mutation. The patient was treated with almonertinib, resulting in significant clinical improvement and successful extubation after nine days. Radiographic imaging showed substantial reduction in pulmonary lesions, highlighting the efficacy of almonertinib.

**Conclusion:**

This case represents the first documented successful treatment of ARDS induced by EGFR E19 mutated NSCLC using almonertinib. The favorable clinical response observed in this critically ill patient suggests that almonertinib may be a viable therapeutic option for managing severe complications in NSCLC. Further research is necessary to corroborate these findings and optimize dosage and toxicity management strategies for broader clinical application.

**Supplementary Information:**

The online version contains supplementary material available at 10.1186/s12890-024-03292-3.

## Introduction

Lung cancer remains a predominant cause of cancer-related mortality worldwide, with non-small cell lung cancer (NSCLC) comprising approximately 85% of all lung cancer cases [1, 2]. The global burden of NSCLC is significant, with a high incidence and poor prognosis contributing to substantial morbidity and mortality [1]. Among NSCLC subtypes, tumors harboring mutations in the epidermal growth factor receptor (EGFR) gene represent a critical clinical subset [3]. EGFR mutations, particularly exon 19 deletions and exon 21 L858R point mutations, are present in roughly 10–15% of NSCLC cases in Western populations and up to 40% in Asian populations [4, 5].

Targeted therapies, specifically tyrosine kinase inhibitors (TKIs), have revolutionized the treatment landscape for EGFR-mutated NSCLC [6, 7]. TKIs such as gefitinib, erlotinib, and afatinib inhibit the tyrosine kinase activity of the EGFR, thereby blocking downstream signaling pathways that promote tumor cell proliferation and survival [8]. Almonertinib, a third-generation EGFR-TKI, has emerged as a potent therapeutic option for patients with EGFR-mutated NSCLC [9]. Its enhanced efficacy and safety profile, characterized by its ability to cross the blood-brain barrier and limited activity against wild-type EGFR, make it a valuable treatment option for EGFR-mutated NSCLC [10, 11].

Despite the advancements in targeted therapy, the management of severe complications such as acute respiratory distress syndrome (ARDS) in NSCLC patients remains challenging [12]. ARDS, a life-threatening condition characterized by acute hypoxemia and bilateral pulmonary infiltrates, complicates the clinical course of NSCLC and is often associated with poor outcomes [13]. Notably, there have been no previous case reports detailing the use of TKIs, specifically almonertinib, in the treatment of NSCLC-induced ARDS. This case report aims to fill this gap by presenting the first documented instance of successful treatment of ARDS induced by EGFR-mutated NSCLC using almonertinib.

## Case presentation

A 63-year-old Chinese Han female presented to our hospital on January 11, 2024, with complaints of progressive dyspnea on exertion for over 20 days, which had acutely worsened in the past day. She reported experiencing unexplained exertional dyspnea, alleviated by rest, since December 20, 2023. This was associated with palpitations, cough, chest tightness, and production of white sputum, without accompanying symptoms such as fever, chills, headache, chest pain, or hemoptysis. The patient had no history of smoking but had been exposed to second-hand smoke for 30 years. She mentioned a previous health examination in August 2023 that indicated a potential lung malignancy, which was not followed up due to a lack of concern from her and her family. The patient denied any additional comorbidities or family history of malignant tumors.

**Fig. 1:**
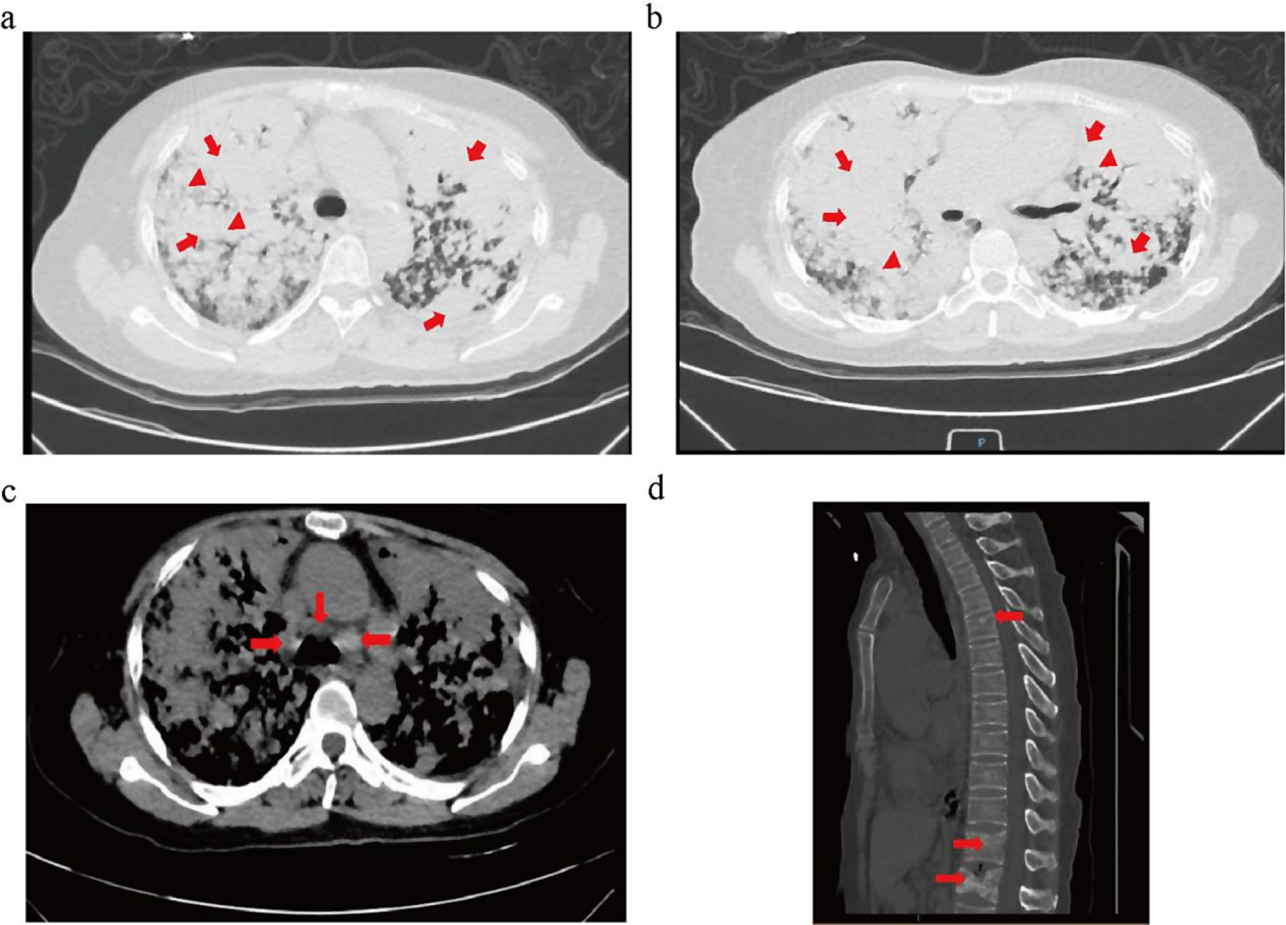
Chest computed tomography (CT) images at initial hospitalization. The scan reveals multiple nodules and plaques in both lungs (**a**, **b**, red arrow), air bronchial sign (**a**, **b**, red triangle), enlarged mediastinal lymph nodes (**c**, red arrow), and increased density within the thoracic (T5/T12) and lumbar (L1) vertebrae (**d**, red arrow)

On January 10, 2024, the patient experienced a marked exacerbation of symptoms, including dyspnea and chest tightness, with increased severity of episodes following physical exertion. There were no associated symptoms such as fever, chills, headache, chest pain, or hemoptysis. Her lowest recorded oxygen saturation level at home was 74% that day. Consequently, her family called an ambulance, and she was brought to the emergency department of our hospital. A chest computed tomography (CT) scan performed on January 11, 2024, revealed multiple nodules and plaques in both lungs, enlarged mediastinal lymph nodes, a small pericardial effusion, minor left pleural effusion, and increased density in the thoracic (T5/T12) and lumbar (L1) vertebrae (Fig. 1). While receiving high-flow nasal cannula (HFNC) therapy at a flow rate of 70 L/min and an oxygen concentration of 95%, her oxygen saturation fluctuated between 80 and 86%, with an oxygenation index of only 54 on the first day. The carcinoembryonic antigen (CEA) level was greater than 100 ng/ml (reference value < 5 ng/ml). Routine blood analysis upon hospital admission on January 11, 2024, revealed the following: white blood cell count at 14.7 × 10^9^/L, neutrophils at 12.21 × 10^9^/L, hemoglobin at 150 g/L, and platelets at 307 × 10^9^/L. Hypersensitive C-reactive protein was elevated at 47 mg/L (reference range: 0–10 mg/L), and procalcitonin was 0.04 ng/ml (reference range: 0-0.05 ng/ml). Urinalysis and fecal examinations returned normal results. Coagulation studies indicated a fibrinogen level of 5.86 g/L (reference range: 2–4 g/L) and a D-dimer level of 1470 µg/L (reference range: 0–500 µg/L). Biochemical and electrolyte panels showed no significant abnormalities. The patient was diagnosed with severe ARDS according to the Berlin Definition [14]. In the absence of evidence for pulmonary infection, it was postulated that her ARDS was secondary to severe lung cancer. Initially, her family was resistant to tracheal intubation and invasive mechanical ventilation. However, the patient’s condition rapidly declined on January 13, 2024. Despite receiving HFNC oxygen therapy with an oxygen concentration nearing 100%, her oxygen saturation remained between 65 and 75%, accompanied by significant dyspnea and profuse sweating.

The patient was emergently transferred to the respiratory intensive care unit (RICU) on January 13, 2024. Upon arrival, her oxygen saturation was critically low at 72%. Vital signs included a temperature of 36.9 °C, heart rate of 128 beats per minute, respiratory rate of 36 breaths per minute, and blood pressure of 118/80 mmHg. Physical examination revealed confusion, diminished breath sounds bilaterally with audible wet rales, a normal cardiac rhythm, and no pathological murmurs. Endotracheal intubation, facilitated by bronchoscopy, was performed on January 13, 2024, with informed consent from the family.

**Fig. 2:**
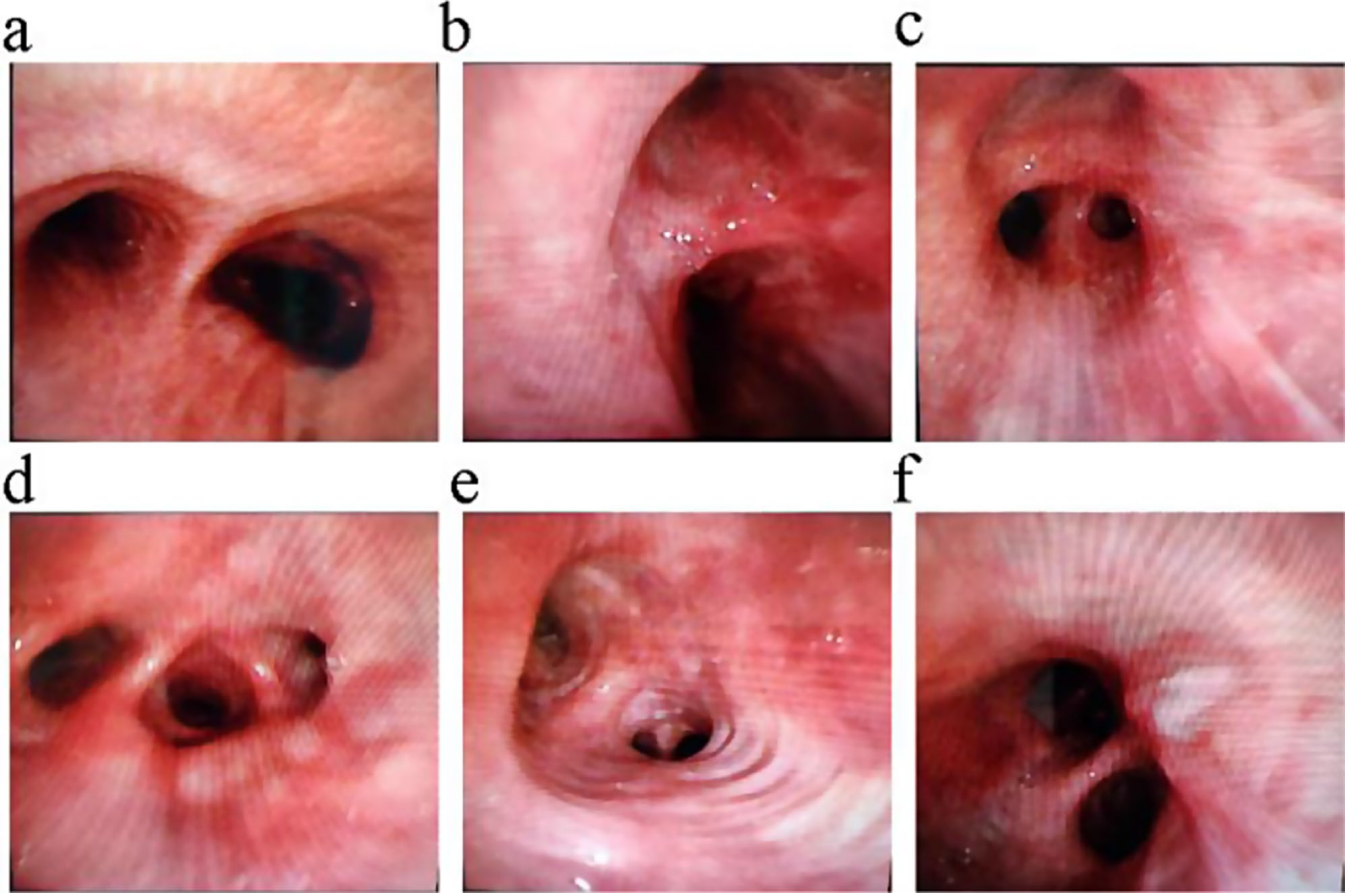
Bronchoscopic images of the bronchial lumen. (**a**) Carina, (**b**) Right second carina, (**c**) Right upper lobe bronchus, (**d**) Right middle lobe and right lower lobe bronchi, (**e**) Left upper lobe and left lingual lobe bronchi, (**f**) Left lower lobe bronchi

On January 15, 2024, sputum culture results revealed the presence of methicillin-resistant Staphylococcus aureus (MRSA). The following day, January 16, 2024, a bronchoscopy was conducted with the intent to obtain a tissue biopsy. The bronchoscopy showed congestion and hypertrophy of the bronchial mucosa, but no evident neoplastic formations were observed (Fig. 2). However, due to the severity of the patient’s ARDS, tissue biopsies could not be completed as planned. Instead, bronchoalveolar lavage (BAL) and brushing were performed on multiple lobes and segments, including the right upper lobe, right middle lobe, basal and dorsal segments of the right lower lobe, left upper lobe, and basal and dorsal segments of the left lower lobe. Metagenomic next-generation sequencing (mNGS) of the BAL fluid detected Candida albicans with a sequence count of 68.

**Fig. 3:**
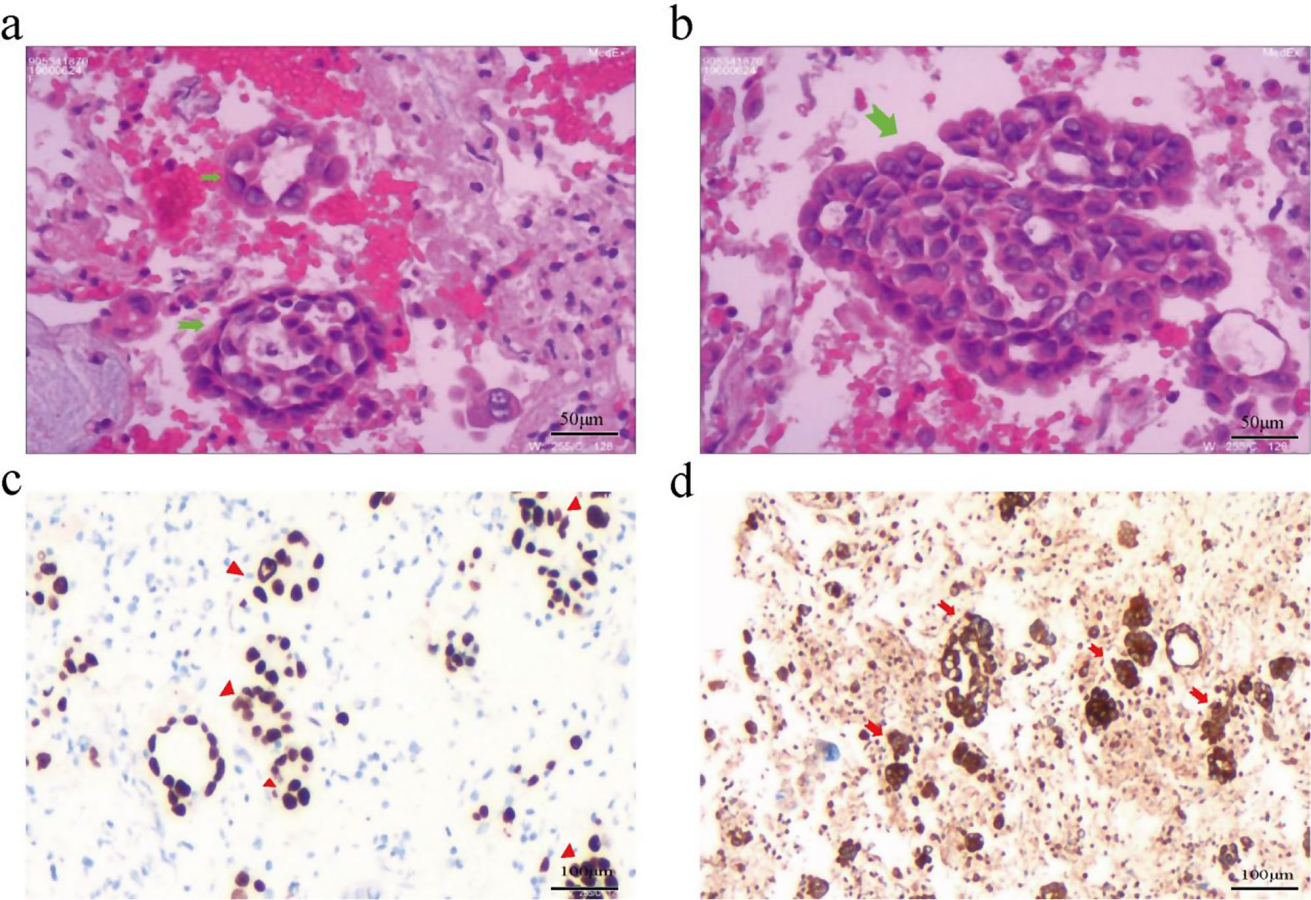
Pathological examination of bronchoalveolar lavage fluid sediment. **a** and **b**. Hematoxylin and eosin (HE) staining indicating adenocarcinoma cells (green arrow) amidst red blood cells; **c**. Immunohistochemical staining showing TTF-1 positivity (red triangle); **d**. Immunohistochemical staining showing CEA positivity (red arrow)

Immunohistochemical analysis of the alveolar lavage fluid sediment, performed on January 18, 2024, revealed positive staining for TTF-1, Napsin A, and CEA, and negative staining for ALK (Ventana), consistent with a diagnosis of lung adenocarcinoma (Fig. 3). Concurrently, next-generation sequencing (NGS) of the blood identified an EGFR exon 19 deletion mutation (Table S1). Consequently, the patient was diagnosed with lung adenocarcinoma (cT4N3M1c, stage IVB) and had an Eastern Cooperative Oncology Group (ECOG) performance status (PS) score of 4. Based on the Chinese Society of Clinical Oncology (CSCO) guidelines and clinical expert consensus, considering the patient with interstitial lung abnormality (ILA) and ARDS at admission. oral almonertinib at a dose of 110 mg once daily as the first-line treatment was initiated on January 18th. Throughout the treatment, the patient did not report any adverse reactions. Given the patient’s hospitalization in the RICU, intubation, and mechanical ventilation, there was a heightened risk of concurrent infections. Therefore, a regimen of antibiotics, including piperacillin-sulbactam, caspofungin, and vancomycin, was administered from January 19, 2024, to January 24, 2024.

**Fig. 4:**
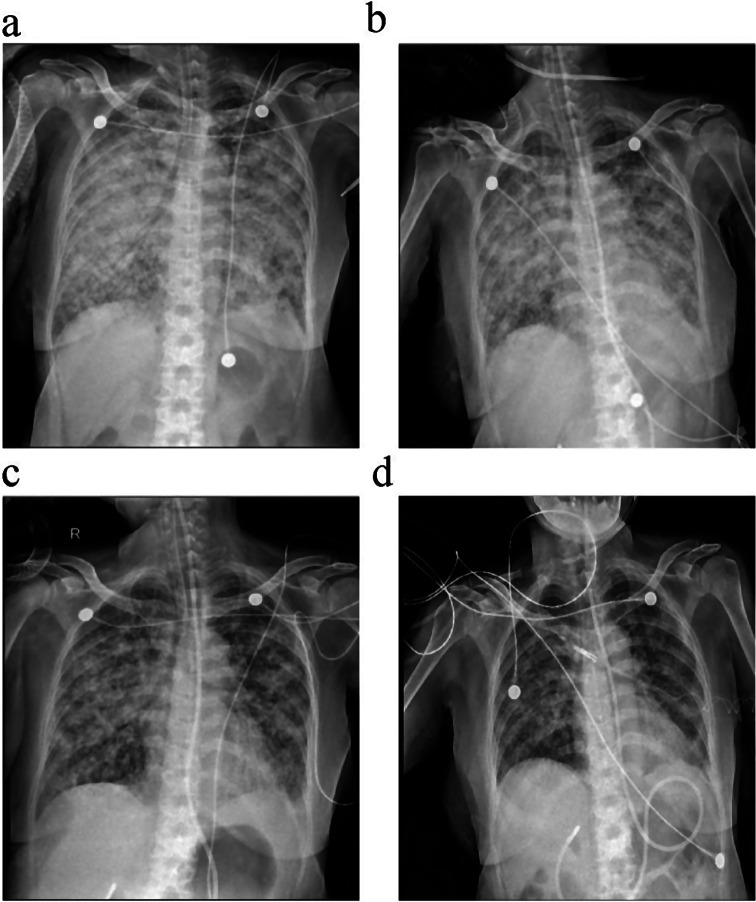
Dynamic changes in chest X-rays before and after treatment. (**a**) Multiple plaques in both lungs on January 13, 2024; (**b**) Minor reduced plaques on January 17, 2024; (**c**) Partial remission on January 21, 2024; (**d**) Significant remission on January 26, 2024

**Fig. 5:**
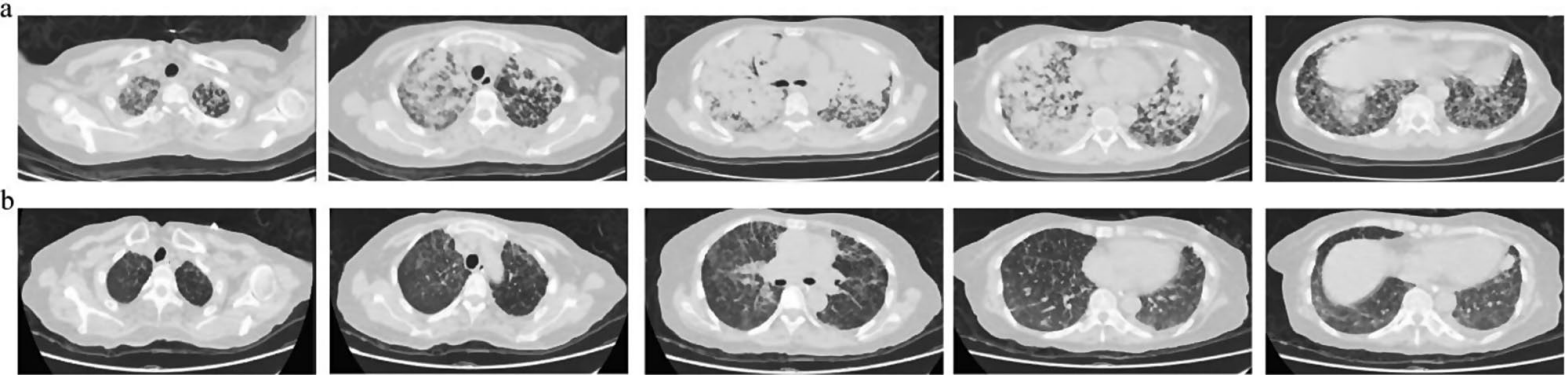
Significant improvement in chest computed tomography (CT) findings before and after treatment. (**a**) Multiple nodules and plaques in both lungs at initial hospitalization on January 11, 2024; (**b**) Resolution of diffuse nodular and patchy densification shadows in both lungs on January 30, 2024

On January 21, 2024, a chest X-ray demonstrated partial remission of multiple nodules and plaques in the lungs (Fig. 4). The patient was successfully extubated on January 22 and transferred out of the RICU on January 25. Continued administration of almonertinib resulted in progressive radiographic improvement, as evidenced by follow-up X-rays (Fig. 4). A chest CT scan on January 30, 2024, showed significant improvement compared to previous imaging (Fig. 5). Ultimately, the patient achieved an oxygenation index of 200 and was discharged on January 31, without any abnormal results of creatine phosphokinase (CPK), aspartate aminotransferase (AST), alanine aminotransferase (ALT), leukocyte and hemoglobin. Besides, she underwent a PET CT scan (Data S1) on March 1, 2024, which also showed that the overall disease control of the patient was satisfactory and the primary lesion reached partial response (PR). Considering the effectiveness of the treatment, we have not adjusted the antitumor protocol. Follow-up in the outpatient clinic is presently ongoing to monitor for recurrences every month and the patient has no rash, diarrhea or dyspnea and is still maintaining a good PS.

## Discussion

This case report delineates the successful management of a critically ill patient with advanced NSCLC complicated by ARDS. The patient, a 63-year-old Chinese Han female, presented with severe dyspnea and rapidly worsening respiratory function. Diagnostic workup, including immunohistochemical analysis and NGS, confirmed lung adenocarcinoma with an EGFR exon 19 deletion. Despite a poor performance status (ECOG score of 4) and severe ARDS, almonertinib therapy led to remarkable clinical improvement. Almonertinib, a third-generation EGFR TKI, was selected for its efficacy and ability to overcome resistance mutations. The patient’s treatment was complicated by potential secondary infections, necessitating broad-spectrum antibiotics. Notably, almonertinib was well tolerated, resulting in significant radiographic and clinical improvement, culminating in successful extubation and discharge.

Lung cancer remains one of the most prevalent and lethal malignancies globally [1, 2]. Traditional treatment modalities, including surgery, chemotherapy, radiotherapy, and interventional therapies, have been the cornerstone of management. However, recent advancements in targeted therapies, antiangiogenic agents, and immune checkpoint inhibitors (ICIs) have significantly extended survival rates for lung cancer patients. Most clinical trials, however, have predominantly included patients with ECOG PS scores of 0 to 1, excluding those with higher PS scores of 3 or 4. Consequently, the current guidelines often recommend supportive care for these patients due to a paucity of high-quality evidence supporting other interventions. In real-world clinical practice, approximately 25% of lung cancer patients present with or develop a PS score of 3 or 4 during their treatment course [15]. Despite their poor prognosis, some patients with high PS scores may benefit from individualized anti-tumor treatments coupled with appropriate life-support measures. The concept of “advanced severe lung cancer,” introduced in 2017, differentiates these patients from those with end-stage disease. This classification includes stage IIIB, IIIC, and IV lung cancers with a PS score of 2–4, which may be influenced by the disease or its treatment but can potentially respond to systemic anti-tumor therapies [16]. Patients with severe lung cancer necessitate a comprehensive treatment approach, integrating multiple modalities such as surgery, radiotherapy, interventional techniques, targeted anti-tumor drugs, and advanced life-support technologies, including ventilators, artificial liver and kidney support, and extracorporeal membrane oxygenation (ECMO) [16]. This case highlights the successful management of a patient with advanced NSCLC complicated by severe ARDS, who experienced rapid recovery through a multidisciplinary approach involving mechanical ventilation, targeted therapy with almonertinib, antibiotic therapy, and so on. The patient’s PS score improved from 4 to 2, demonstrating the potential reversibility and fluctuation of PS scores with appropriate intervention.

ARDS is a critical condition responsible for approximately 10% of admissions to intensive care units (ICU) and 23% of mechanically ventilated patients, with a mortality rate reaching up to 45% in severe cases [17]. ARDS is characterized by acute hypoxemic respiratory failure accompanied by bilateral infiltrates on chest imaging, not fully attributable to cardiac failure or fluid overload [14, 18]. It is typically triggered by a variety of risk factors, including pneumonia, non-pulmonary sepsis, gastric aspiration, trauma, pancreatitis, burns, inhalation injury, drug overdose, multiple transfusions, or shock [19]. Currently, there are no reported cases in the literature specifically addressing the treatment of ARDS caused by lung cancer. However, there have been case reports of patients with NSCLC who required mechanical ventilation due to respiratory failure and were treated successfully with targeted therapies [20]. These reports indicate that targeted therapy may have a therapeutic benefit in mechanically ventilated patients. For instance, the use of ALK inhibitors like crizotinib [21] and alectinib [22] in ALK-positive NSCLC patients has shown promising outcomes, including improved performance status and successful weaning from mechanical ventilation. Additionally, EGFR-TKIs have demonstrated significant efficacy in improving survival rates and facilitating ventilator weaning in critically ill EGFR-mutant NSCLC patients [23]. In our case, the patient exhibited a notable clinical improvement primarily due to the administration of almonertinib, a third-generation EGFR-TKI, which underscores the potential efficacy of targeted therapy in managing ARDS secondary to advanced lung cancer. While the anti-infective treatment played a supportive role, the rapid and substantial recovery can be largely attributed to the targeted antitumor therapy. This case highlights the importance of considering targeted therapy as a viable treatment option in critically ill patients with oncogene-driven NSCLC and ARDS. Further research is warranted to explore and validate the use of TKIs and other targeted agents in similar clinical scenarios to improve patient outcomes.

Almonertinib, a third-generation EGFR TKI, has emerged as a potent therapeutic agent for NSCLC harboring EGFR mutations [24]. Its mechanism of action involves the replacement of the methyl group on the indole nitrogen with a cyclopropyl group, enhancing stability and allowing for irreversible covalent binding with the ATP-binding domain of EGFR. This modification enables almonertinib to efficiently inhibit both activating mutations (such as exon 19 deletions and L858R) and resistance mutations (such as T790M), while minimizing activity against wild-type EGFR [24, 25]. Additionally, almonertinib improves penetration of the blood-brain barrier, effectively suppressing brain and spinal cord metastases, which is crucial for advanced NSCLC patients [10, 11]. Clinical studies have demonstrated the efficacy and safety of almonertinib in treating patients with EGFR-mutated NSCLC. In the phase II clinical trial APOLLO, almonertinib showed a primary endpoint overall response rate of 68.9%, with a median progression-free survival (PFS) of 12.4 months [26]. Notably, patients with L858R mutations and exon 19 deletions achieved similar benefits in terms of PFS and overall survival (OS) [27]. On December 16, 2021, almonertinib was approved by the National Medical Products Administration as first-line treatment of locally advanced or metastatic NSCLC with 19Del and 21L858R mutation. The design of almonertinib, which incorporates a cyclopropyl group, reduces the production of metabolites that inhibit wild-type EGFR, thereby decreasing adverse effects commonly associated with EGFR inhibition [25]. The main adverse events of almonertinib are elevation of CPK (19.6%), AST (12.3%), and ALT (11.4%), rash (12.7%), leukopenia (11.2%) and diarrhea (7.4%) [24], which are superior to other EGFR-TKIs. The consensus is that patients with concurrent or previous interstitial lung disease (ILD) are more likely to develop targeted drugs-induced ILD and have a higher mortality. ILD is a deadly adverse effect of EGFR-TKIs such as gefitinib, osimertinib, however, in almonertinib this has been rare. Recently some cases of almonertinib-induced ILD have been also reported [28, 29]. Besides, Ting Li and her colleague has reported that one patient who received almolertinib and anlotinib experienced severe pulmonary embolism (PE) [30]. In our case report, almonertinib exhibited significant anti-tumor activity, leading to rapid symptomatic improvement and favorable chest imaging results. This underscores its potential as an effective treatment for severe NSCLC, particularly in patients with critical complications like ARDS. Continued follow-up, which will be essential to evaluate the long-term effects and safety profile of almonertinib in this patient population, is ongoing and the patient has no secondary ILD, PE or any other severe complication. These findings contribute to the growing body of evidence supporting the use of advanced EGFR-TKIs in personalized cancer therapy, highlighting the importance of integrating molecular diagnostics and targeted treatments in clinical practice.

## Conclusion

This study is the first to document the successful treatment of ARDS induced by EGFR E19 mutated NSCLC with almonertinib. The remarkable clinical improvement observed in this case underscores the potential benefits of almonertinib as a therapeutic option for patients with severe complications from NSCLC. Despite these promising results, further research is necessary to provide more robust evidence regarding the efficacy of almonertinib, particularly in critically ill patients. Additionally, studies focusing on how to select specific groups of advanced severe lung cancer patients who may benefit from almonertinib are essential. These studies are crucial for fully establishing the therapeutic profile of almonertinib and ensuring its safe application in clinical practice.

## Electronic supplementary material

Below is the link to the electronic supplementary material.

Supplementary Material 1

Supplementary Material 2

## Data Availability

The data that support the fndings of this study are not publicly available due to their containing information that could compromise the privacy of patient but are available from the corresponding author (liyujun_0110@163.com) upon reasonable request. The simplified version of mNGS results during the current study is shown in supplementary Table 1. Further enquiries can be directed to the corresponding author.We have included the information mentioned above in the “Availability of Data and Materials” section in our manuscript.

## Declarations

### Clinical trial number

No clinical trial number was applied for this paper.

## Abbreviations

ARDS: Acute respiratory distress syndrome
NSCLC: Non-small cell lung cancer
EGFR: Epidermal growth factor receptor
TKI: Tyrosine kinase inhibitor
CT: Computed tomography
HFNC: High-flow nasal cannula
CEA: Carcinoembryonic antigen
RICU: Respiratory intensive care unit
MRSA: Methicillin-resistant Staphylococcus aureus
BAL: Bronchoalveolar lavage
mNGS: Metagenomic next-generation sequencing
ECOG: Eastern Cooperative Oncology Group
CSCO: Chinese Society of Clinical Oncology
ICIs: Immune checkpoint inhibitors
ECMO: Extracorporeal membrane oxygenation
PFS: Progression-free survival
OS: Overall survival
