# Supplementary material for: Successful rapid improvement of acute respiratory distress syndrome induced by EGFR-mutated non-small cell lung cancer with almonertinib: a case report
Source: BMC Pulm Med. 2024 Sep 27;24:471. doi: 10.1186/s12890-024-03292-3 (PMC11437641; doi:10.1186/s12890-024-03292-3)
Supplement: Supplementary file 2 — Supplementary Material 2 [file 12890_2024_3292_MOESM2_ESM.pdf]

PET-CT

Name: YEH **Sex:** **Age: 63** **Inspection date: 2024-** **Inspection No.:**

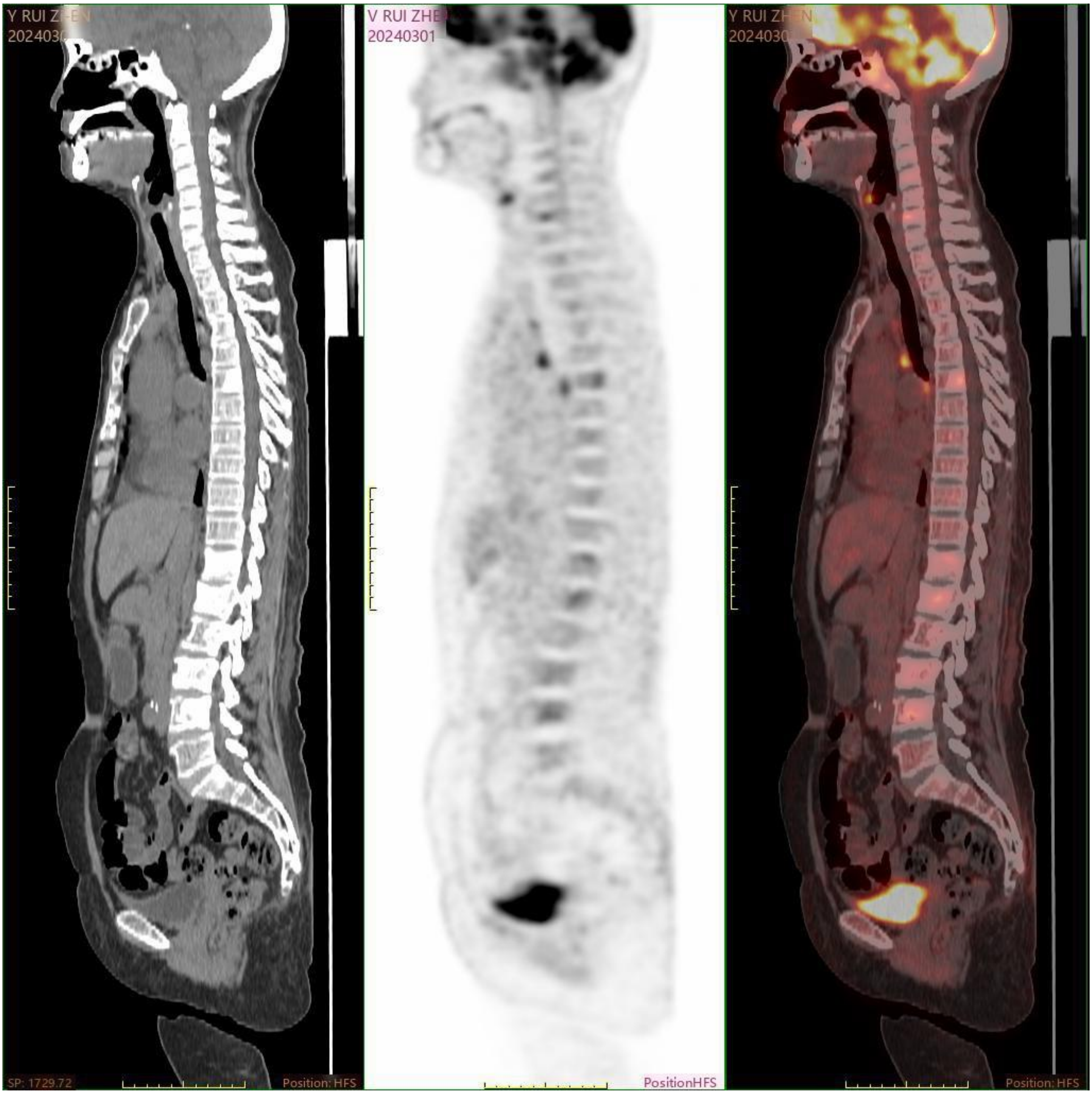

PET-CT

Name: YEH **Sex:** **Age: 63** **Inspection date: 2024-** **Inspection No.:**

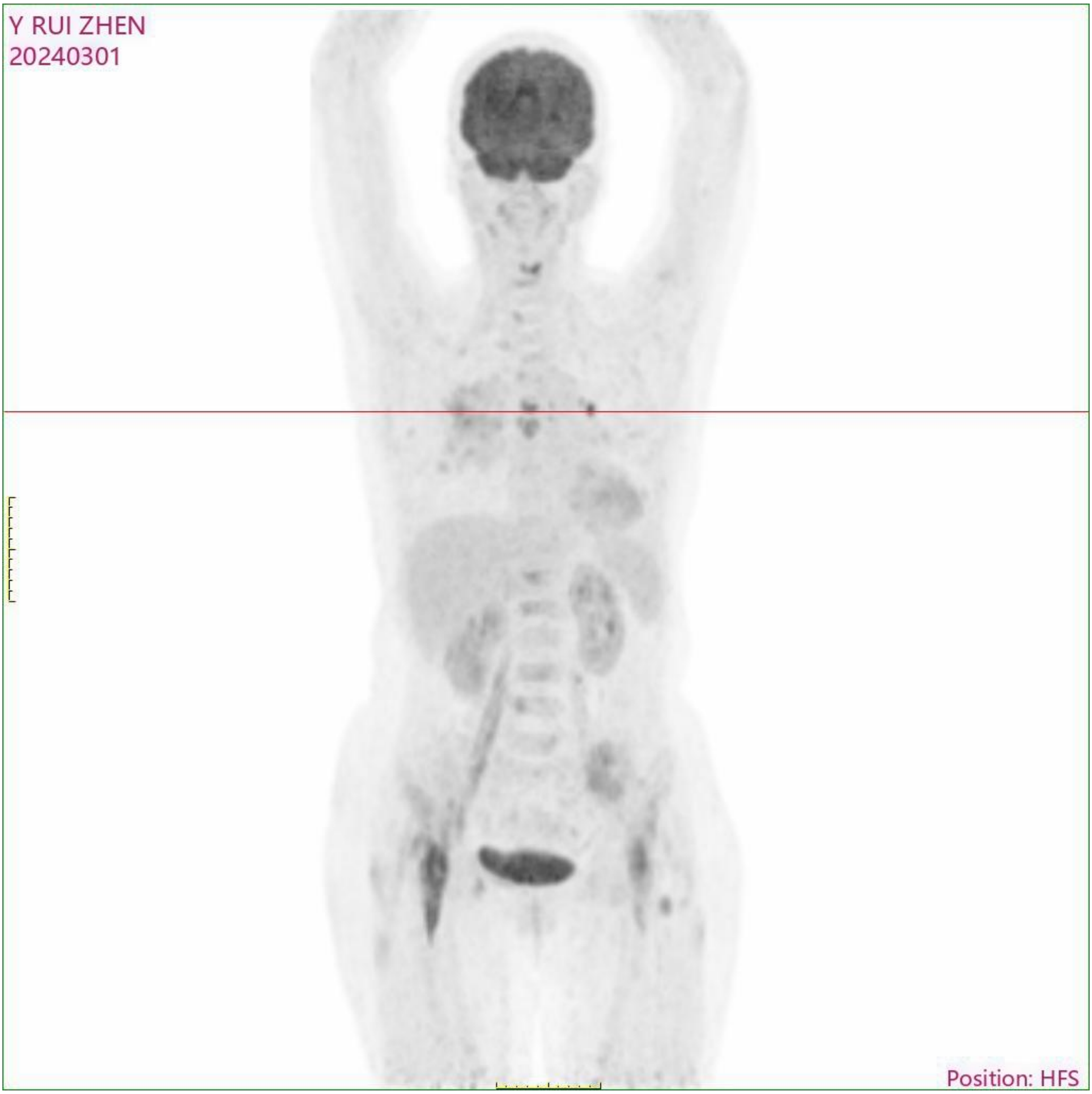

Address:Pavilion 21, No.117 Liuhua Road, Yuexiu Tel: 020-83220688

PET-CT

Name: YEH **Sex:** **Age: 63** **Inspection date: 2024-** **Inspection No.:**

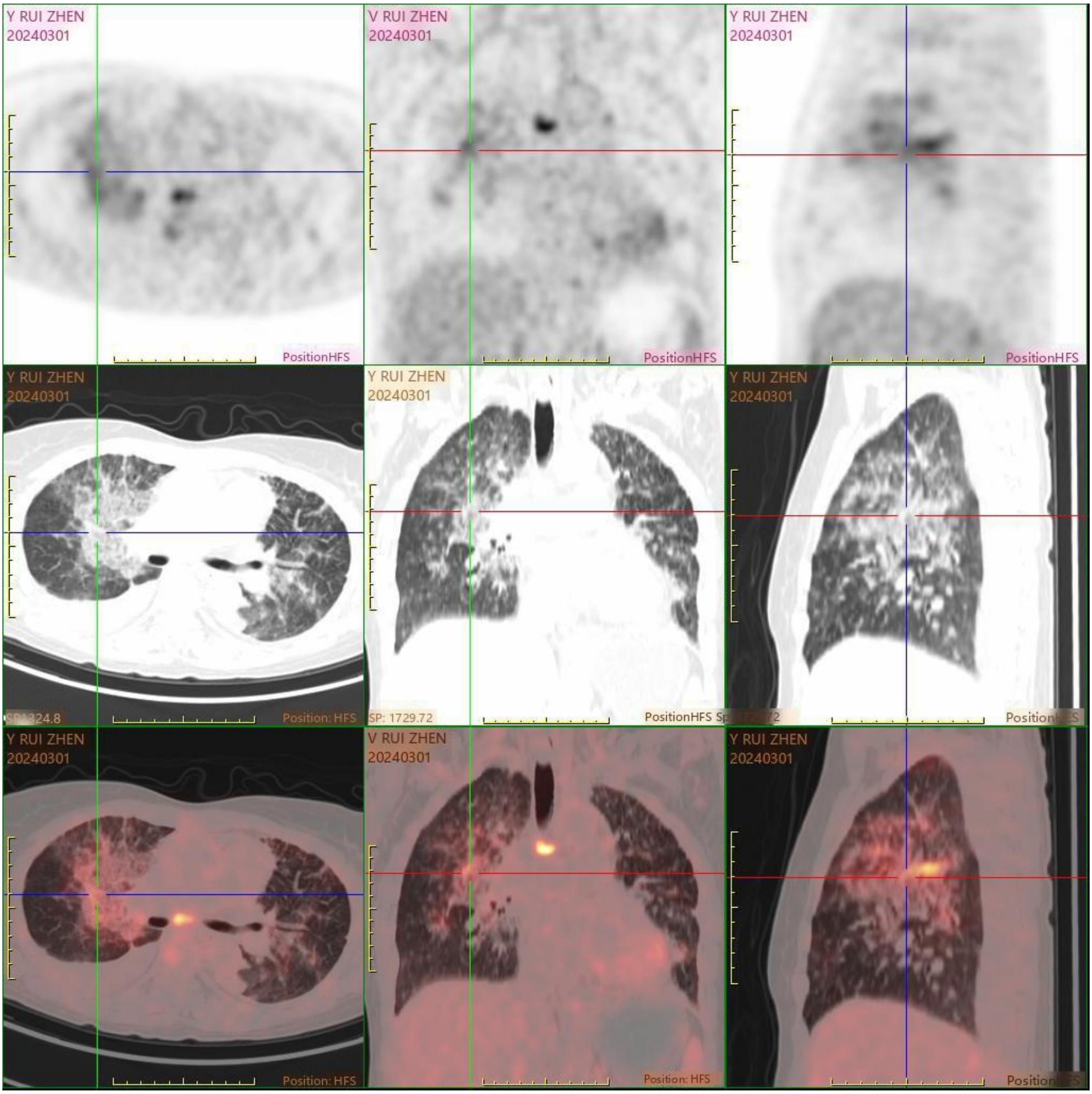

Address:Pavilion 21, No.117 Liuhua Road, Yuexiu Tel: 020-83220688

PET-CT

Name: YEH **Sex:** **Age: 63** **Inspection date: 2024-** **Inspection No.:**

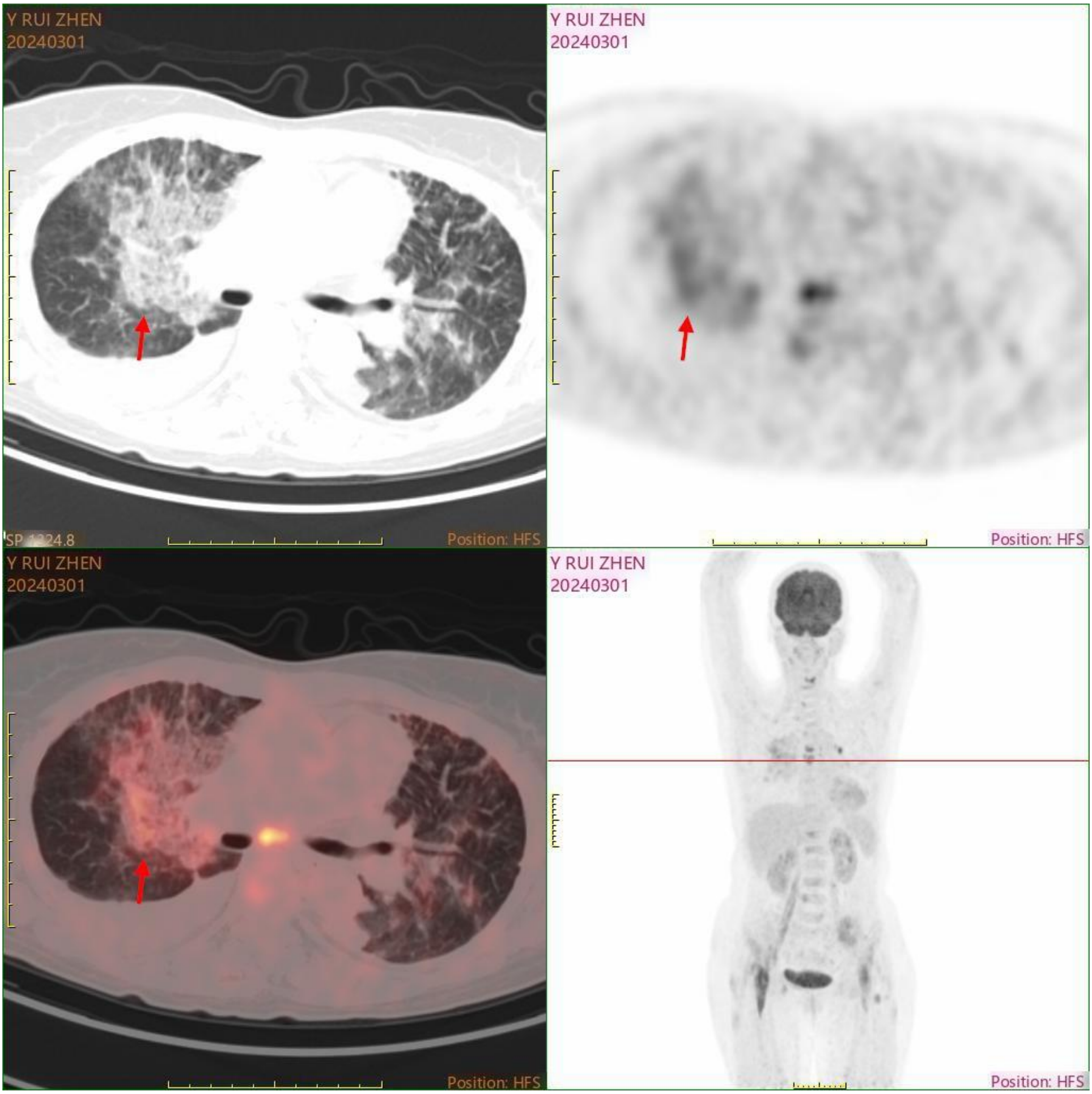

PET-CT

Name: YEH **Sex:** **Age: 63** **Inspection date: 2024-** **Inspection No.:**

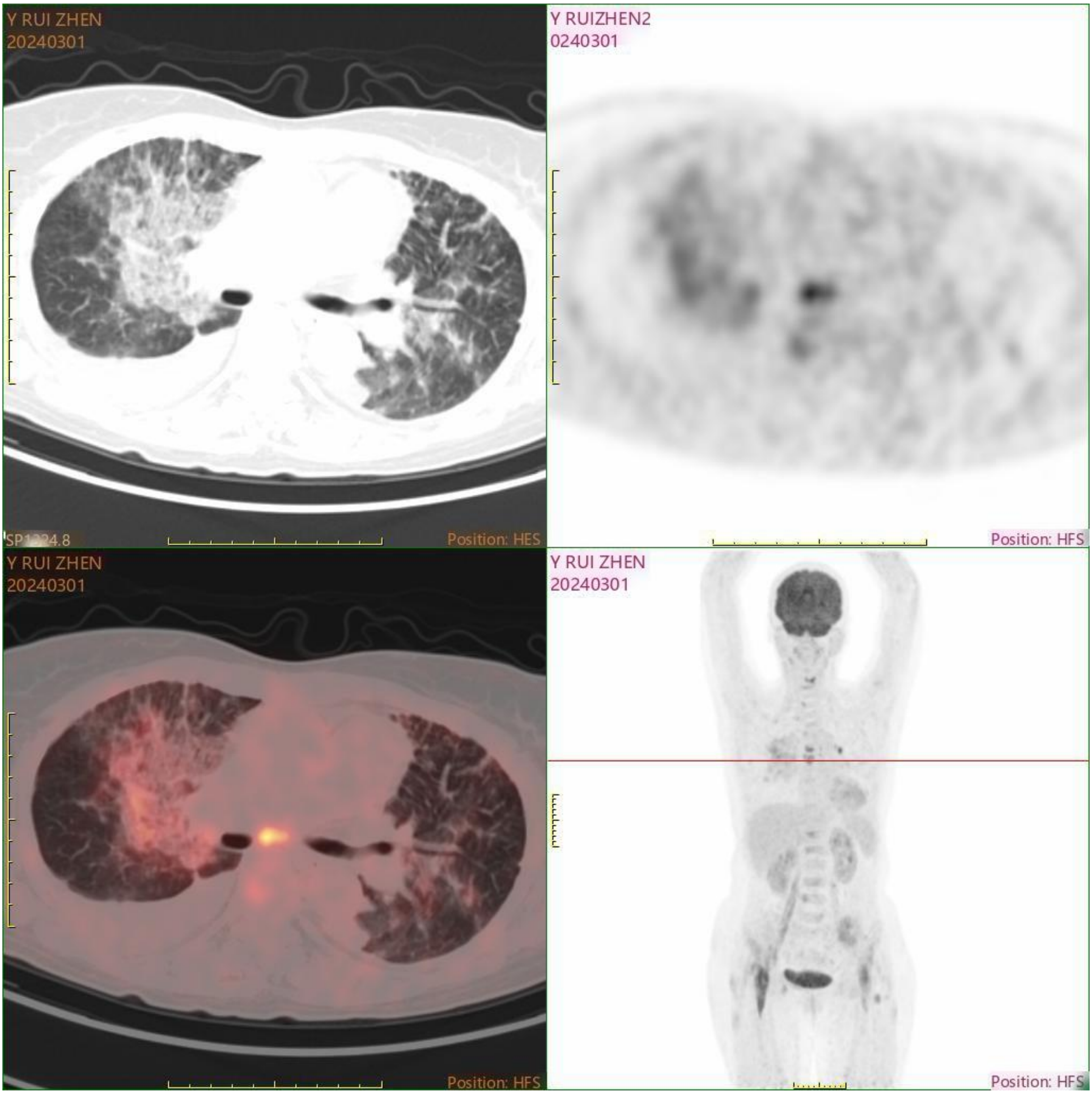

Address:Pavilion 21, No.117 Liuhua Road, Yuexiu
Tel: 020-83220688

PET-CT

Name: YEH **Sex:** **Age: 63** **Inspection date: 2024-** **Inspection No.:**

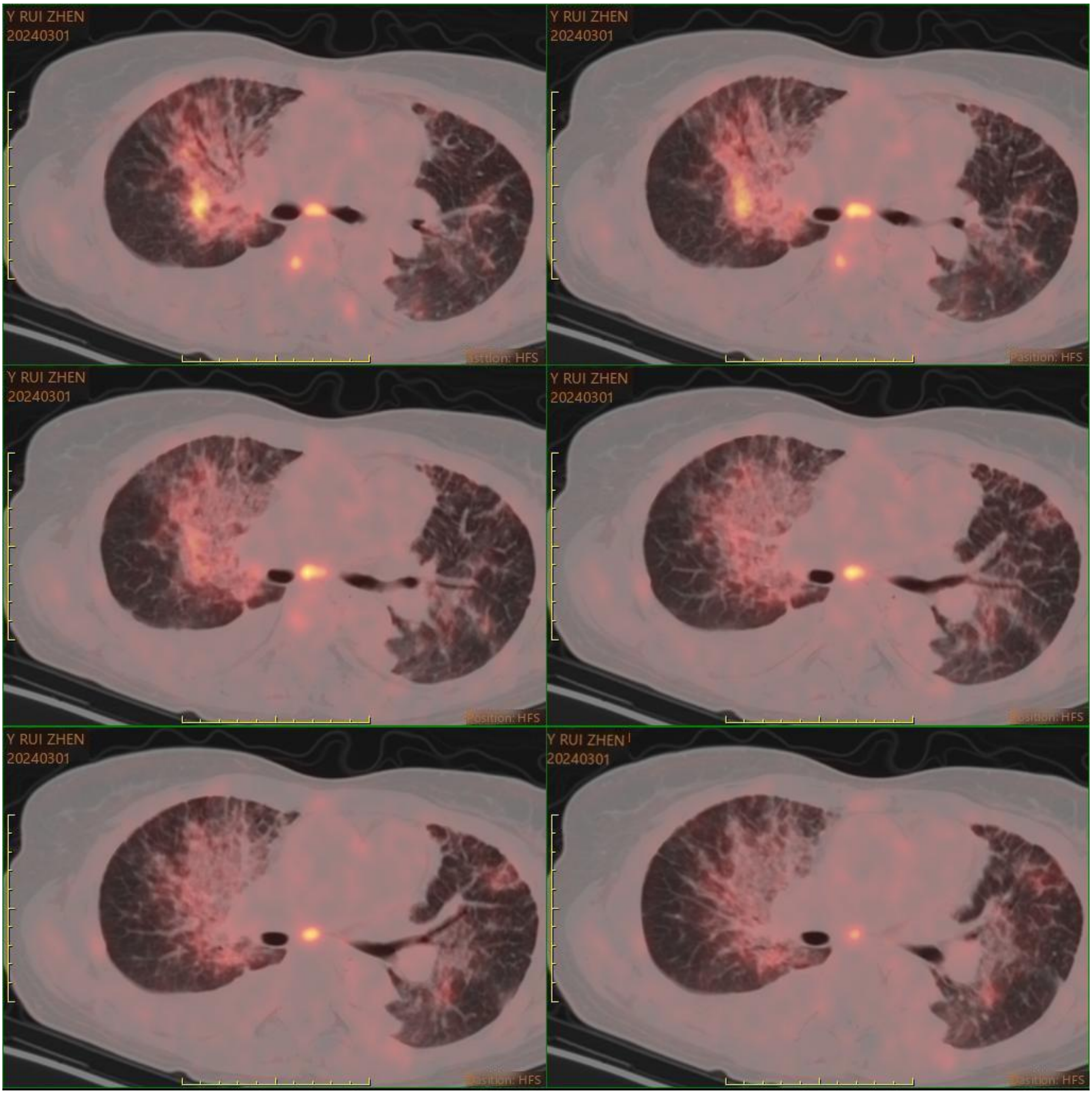

Address:Pavilion 21, No.117 Liuhua Road, Yuexiu Tel: 020-83220688

PET-CT

Name: YEH **Sex:** **Age: 63** **Inspection date: 2024-** **Inspection No.:**

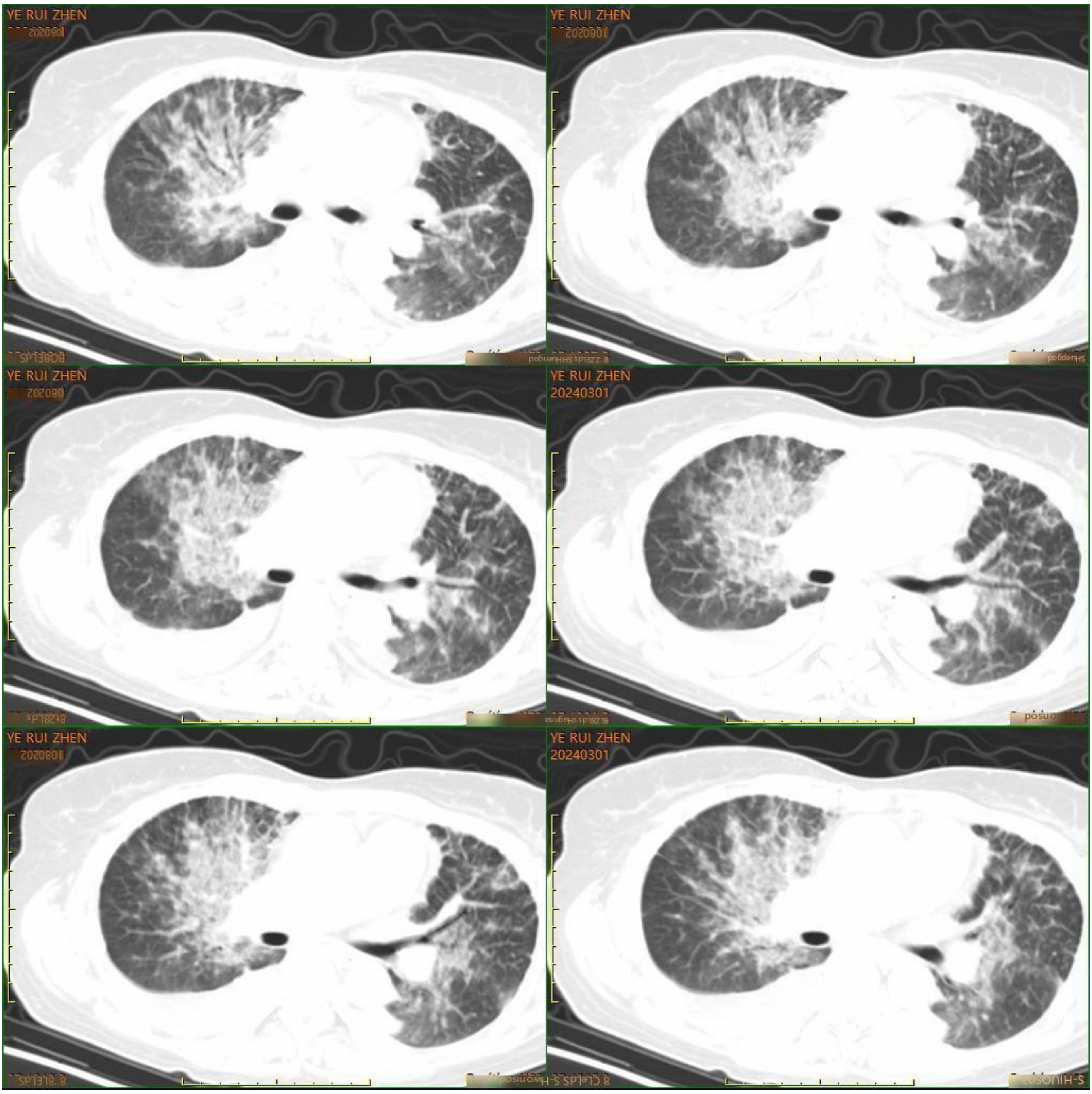

Address:Pavilion 21, No.117 Liuhua Road, Yuexiu Tel: 020-83220688

PET-CT

Name: YEH **Sex:** **Age: 63** **Inspection date: 2024-** **Inspection No.:**

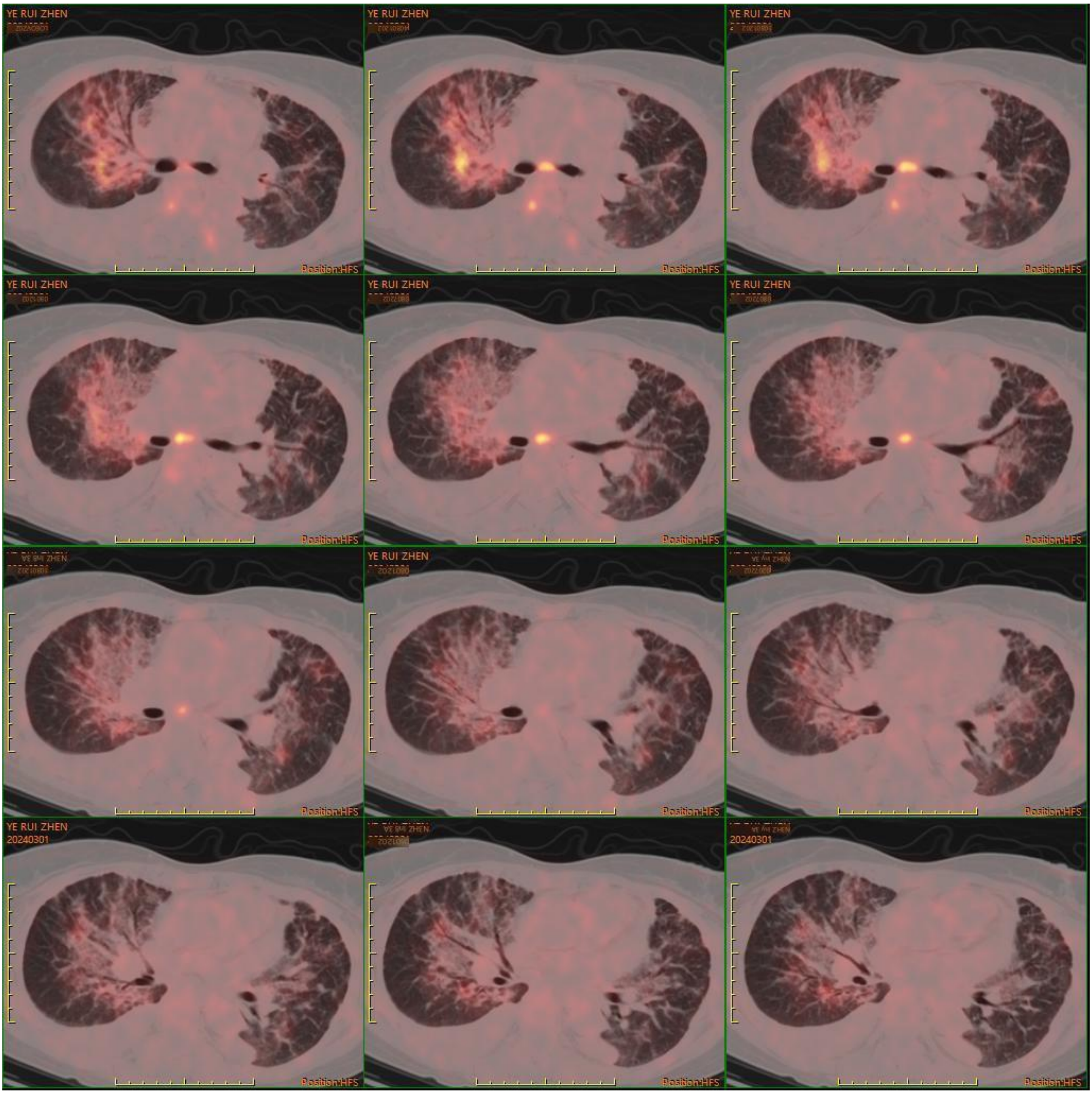

PET-CT

Name: YEH **Sex:** **Age: 63** **Inspection date: 2024-** **Inspection No.:**

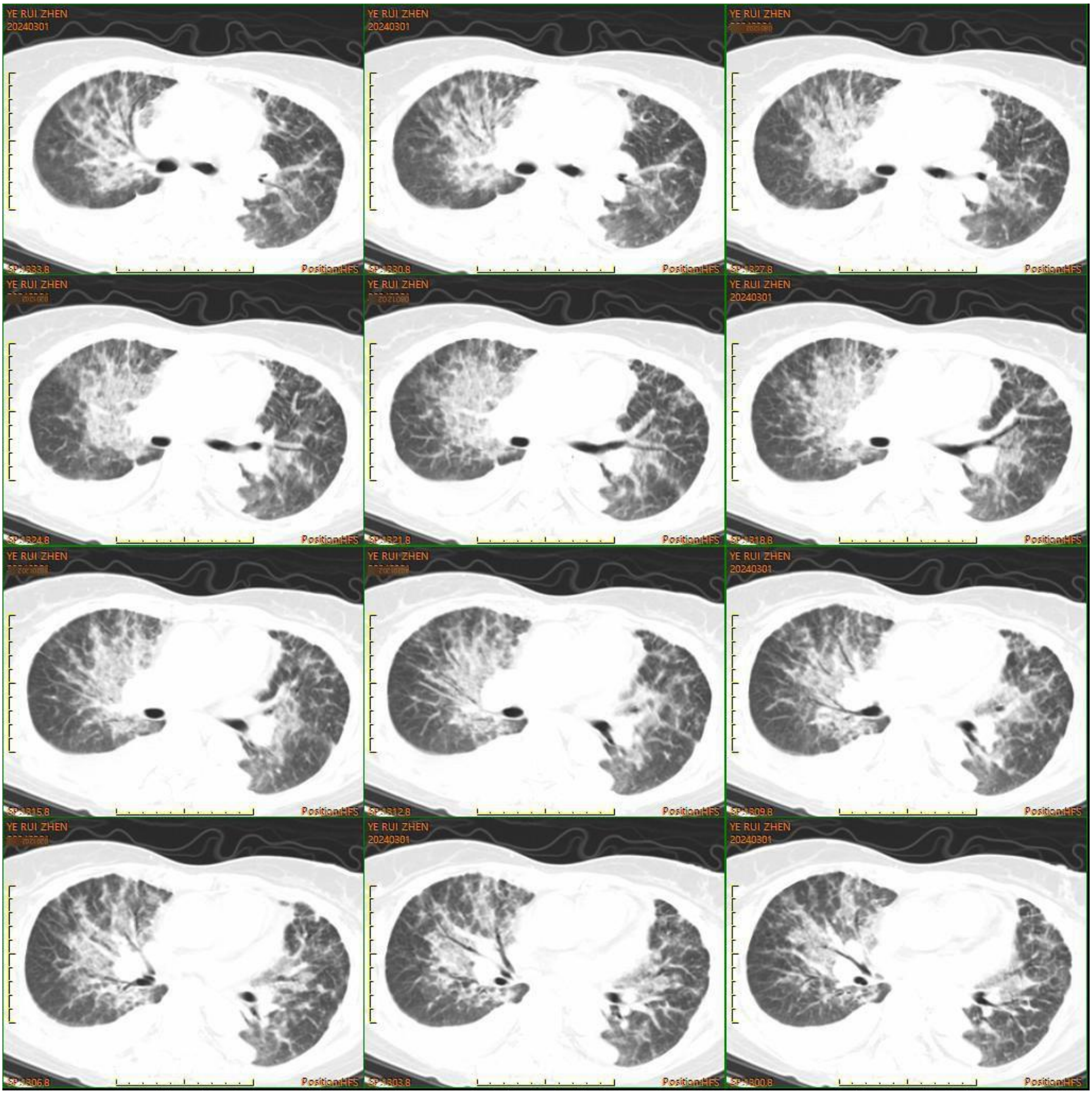

PET-CT

Name: YEH **Sex:** **Age: 63** **Inspection date: 2024-** **Inspection No.:**

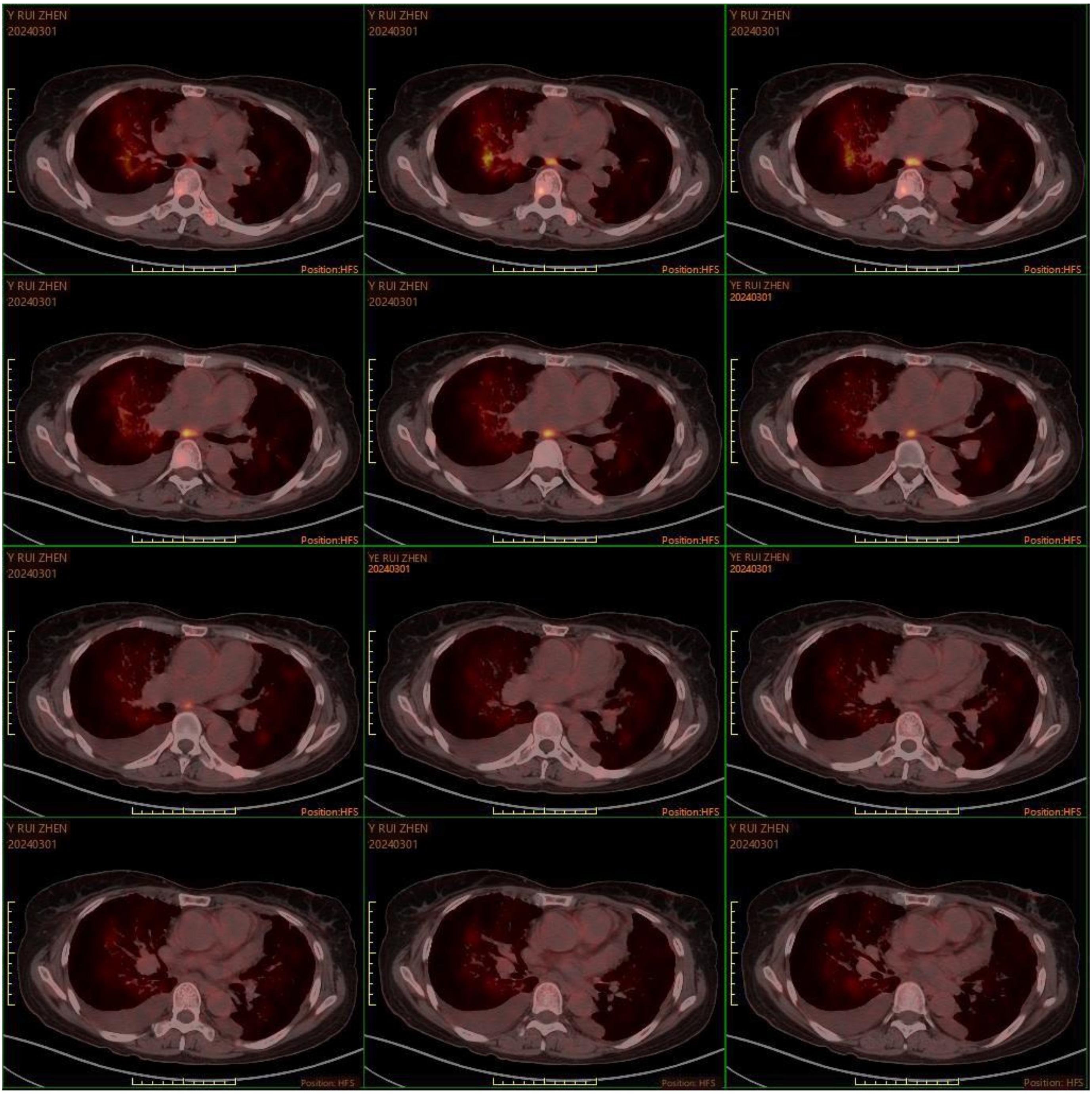

Address:Pavilion 21, No.117 Liuhua Road, Yuexiu Tel: 020-83220688

PET-CT

Name: YEH **Sex:** **Age: 63** **Inspection date: 2024-** **Inspection No.:**

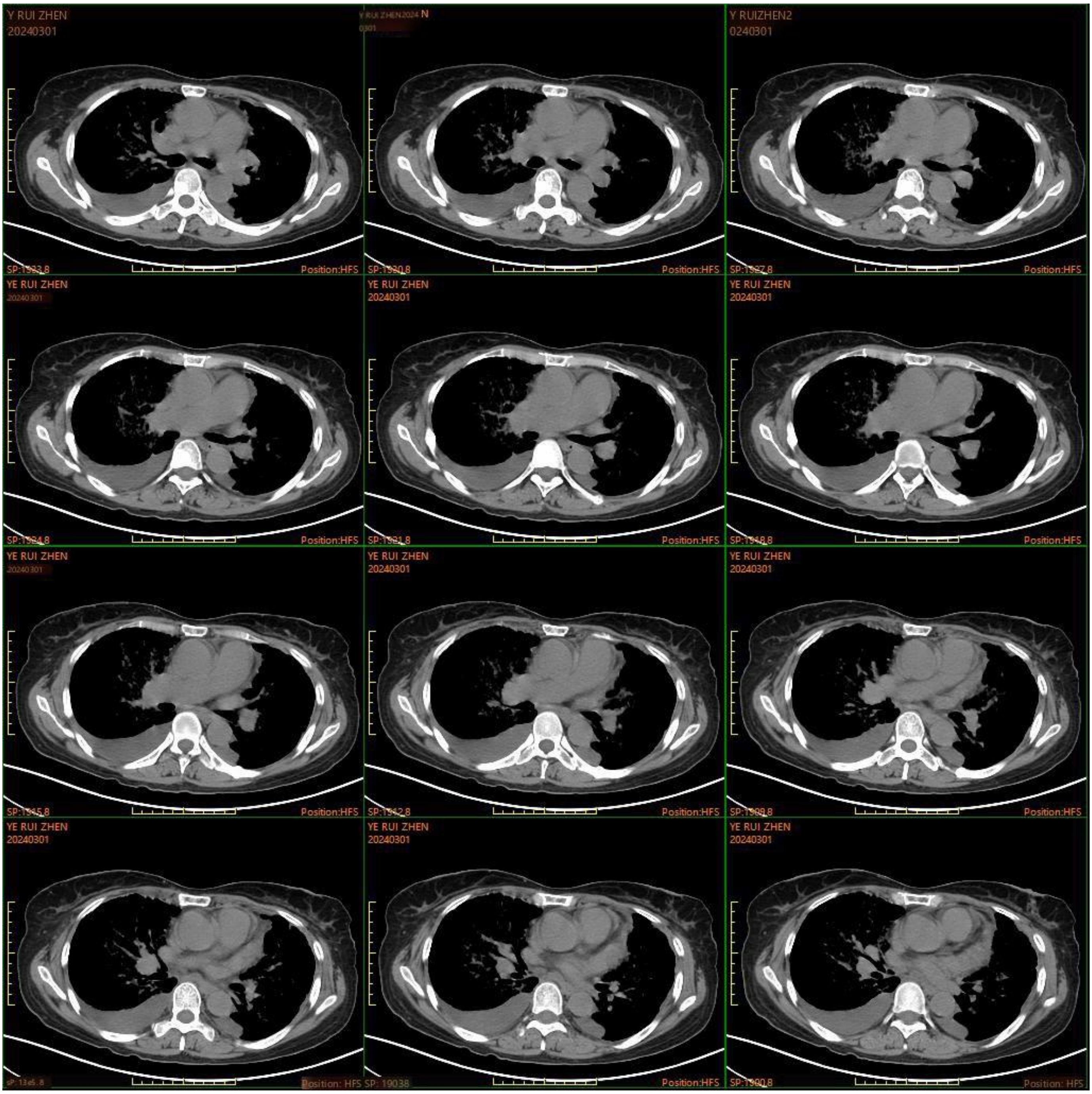

Address:Pavilion 21, No.117 Liuhua Road, Yuexiu
Tel: 020-83220688

PET-CT

Name: YEH **Sex:** **Age: 63** **Inspection date: 2024-** **Inspection No.:**

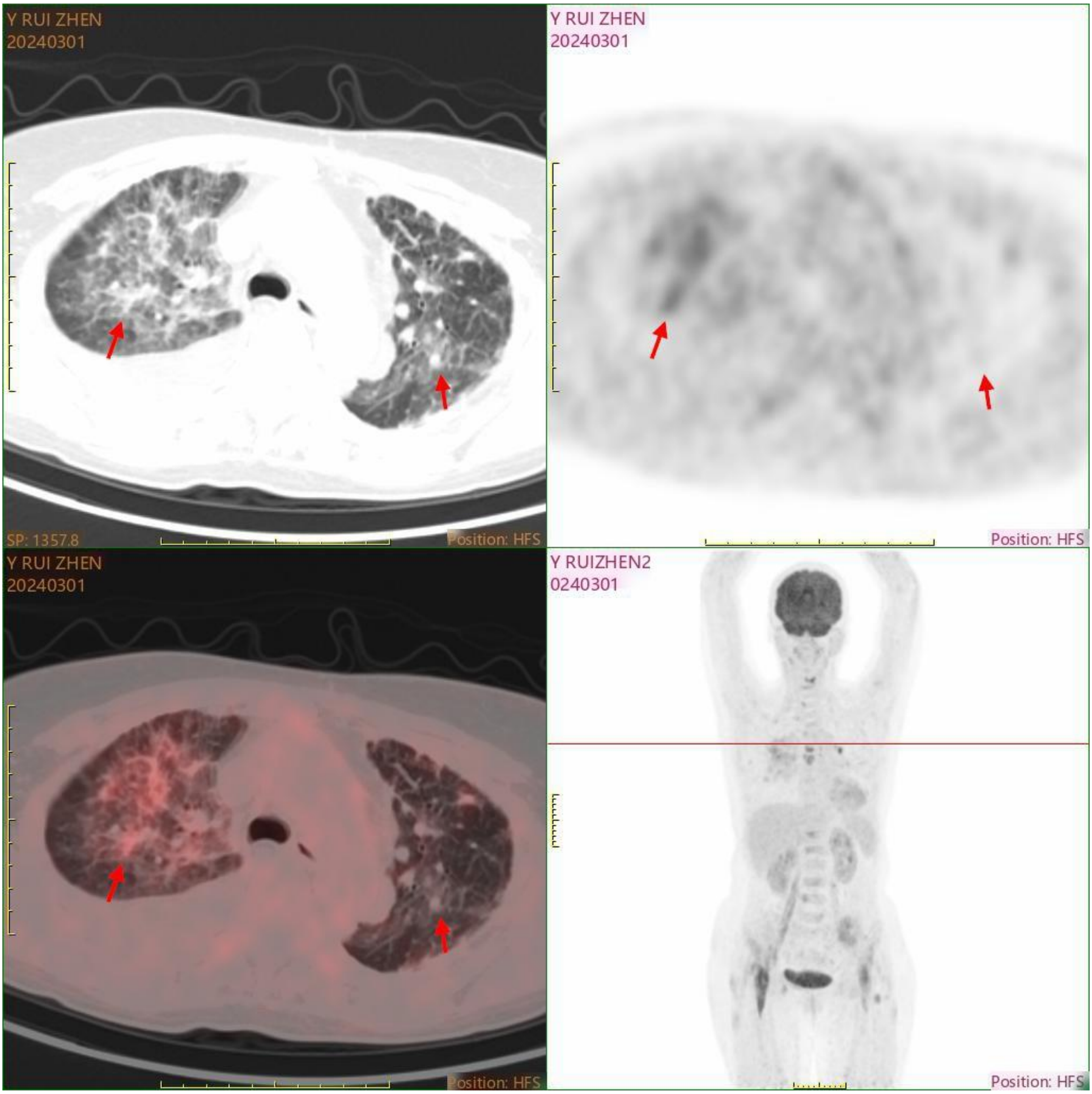

Address:Pavilion 21, No.117 Liuhua Road, Yuexiu Tel: 020-83220688

PET-CT

Name: YEH **Sex:** **Age: 63** **Inspection date: 2024-** **Inspection No.:**

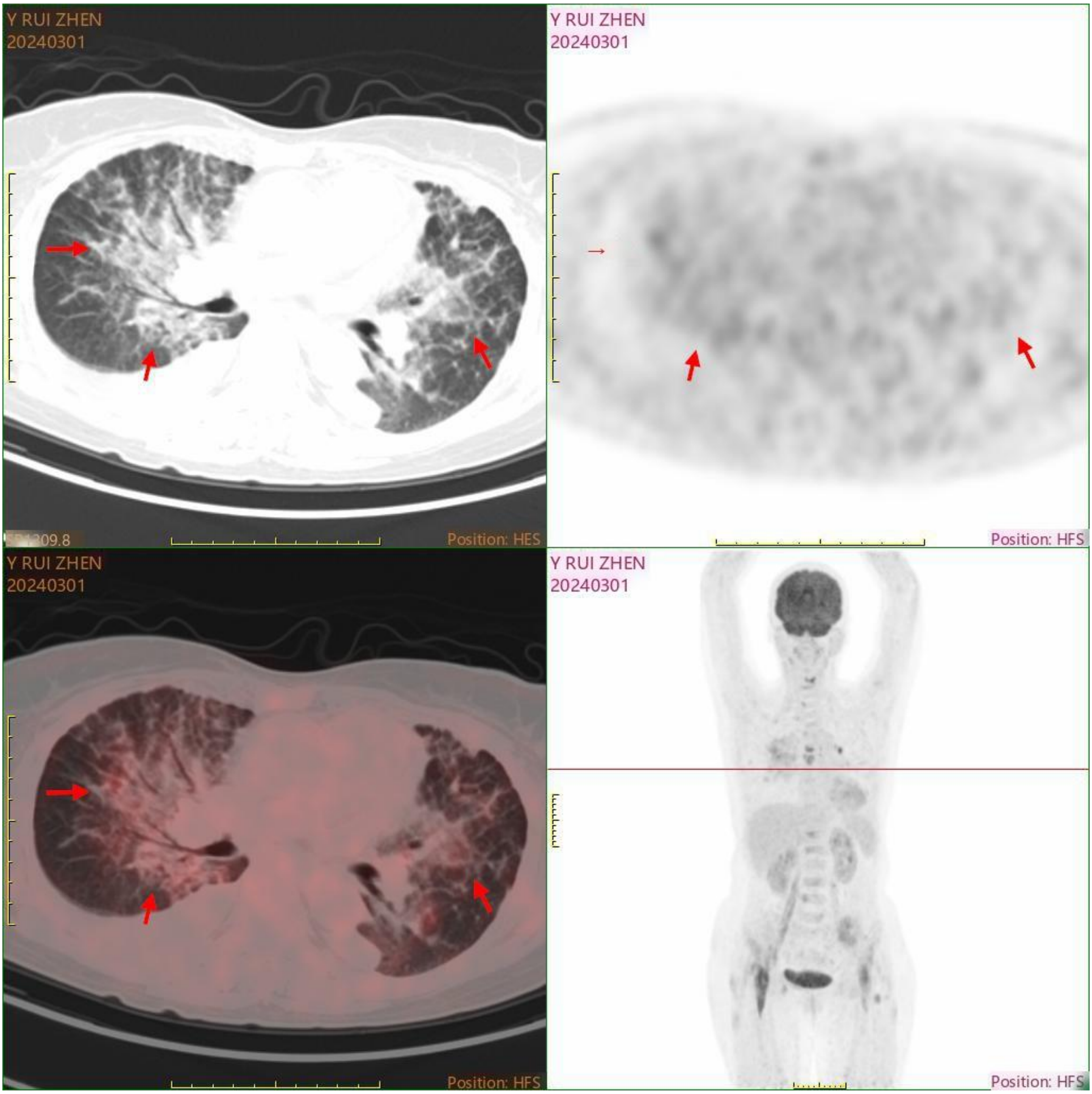

Address:Pavilion 21, No.117 Liuhua Road, Yuexiu Tel: 020-83220688

PET-CT

Name: YEH **Sex:** **Age: 63** **Inspection date: 2024-** **Inspection No.:**

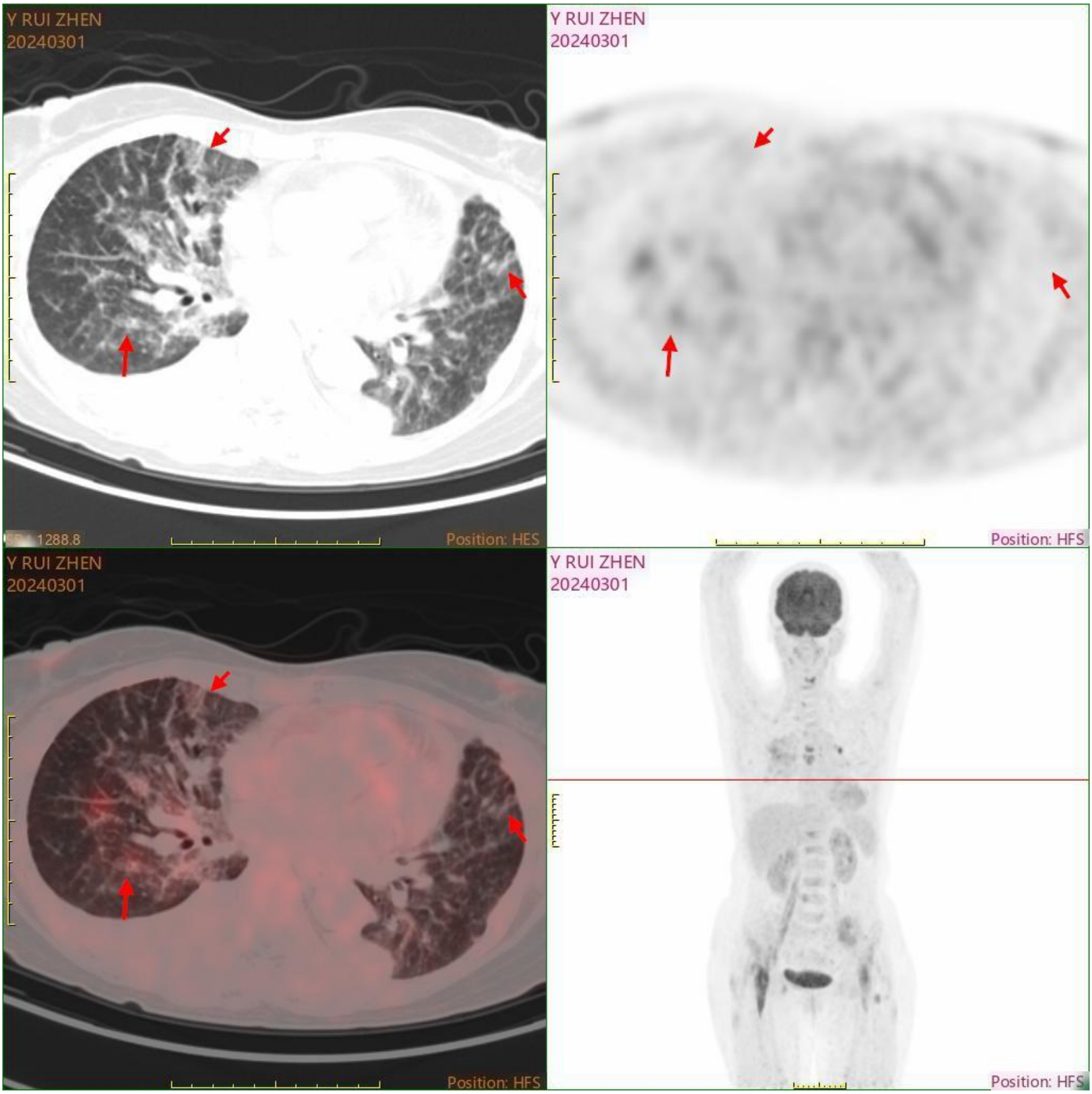

Address:Pavilion 21, No.117 Liuhua Road, Yuexiu Tel: 020-83220688

PET-CT

Name: YEH **Sex:** **Age: 63** **Inspection date: 2024-** **Inspection No.:**

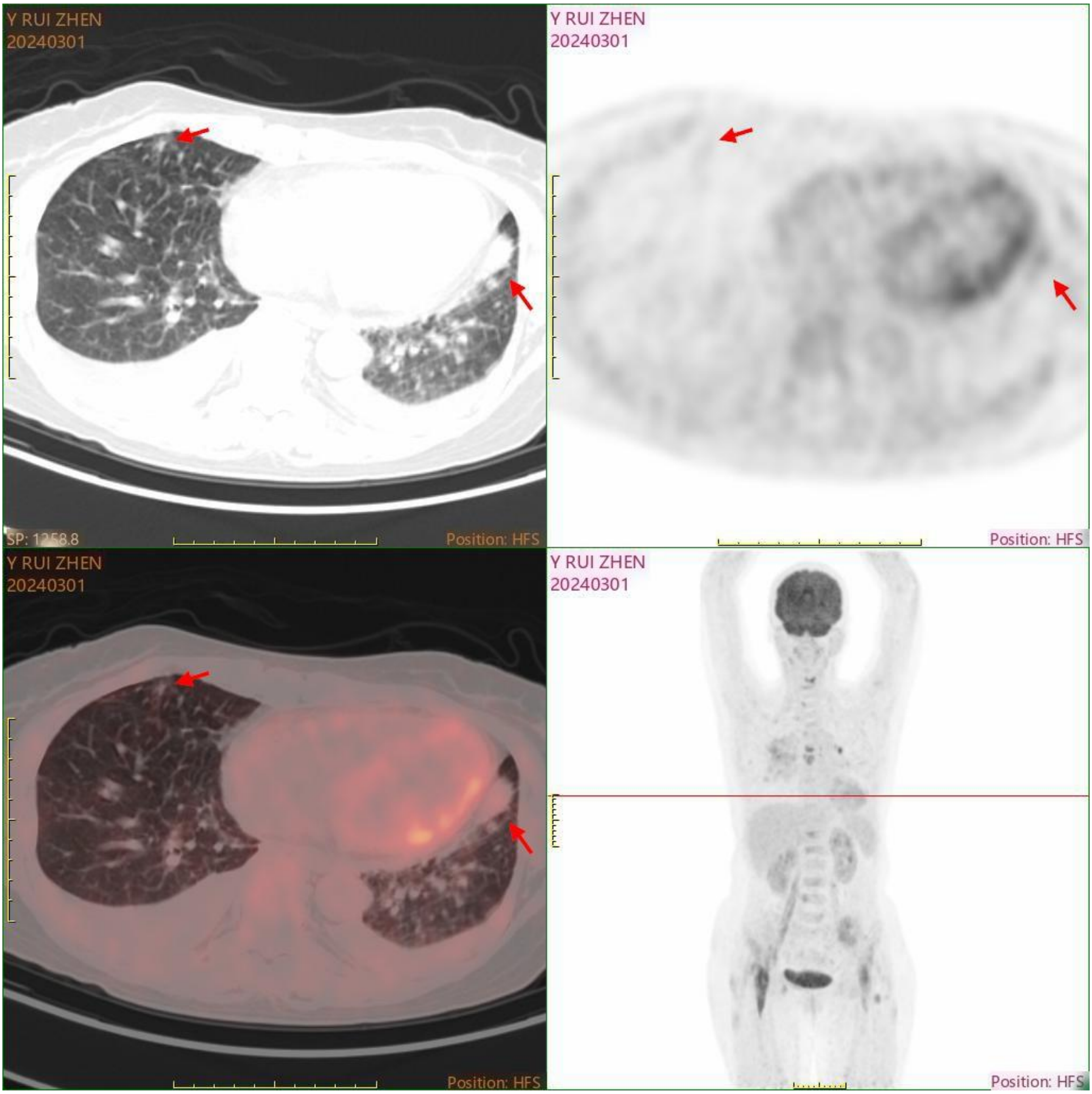

Address:Pavilion 21, No.117 Liuhua Road, Yuexiu Tel: 020-83220688

PET-CT

Name: YEH **Sex:** **Age: 63** **Inspection date: 2024-** **Inspection No.:**

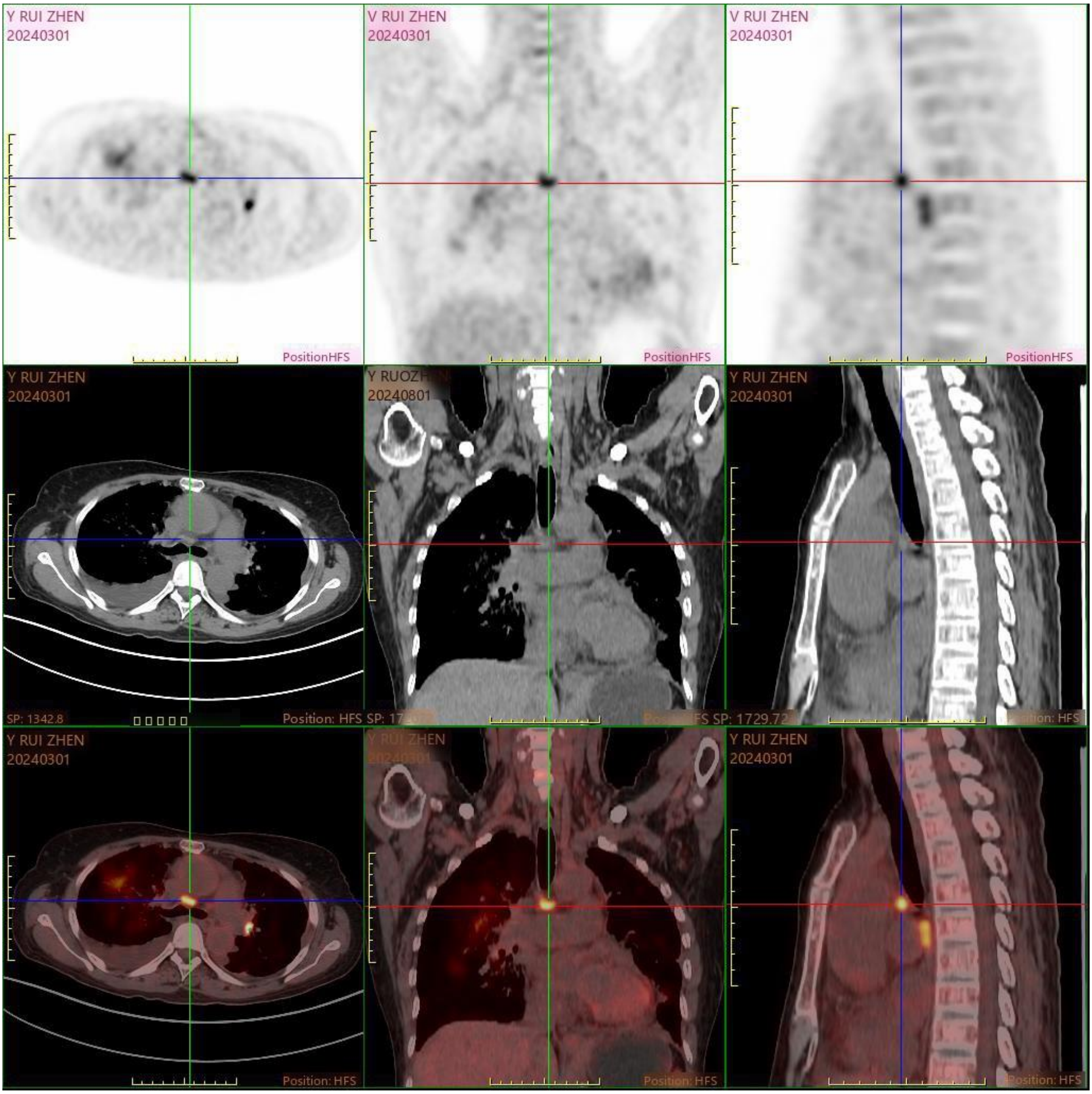

Address:Pavilion 21, No.117 Liuhua Road, Yuexiu Tel: 020-83220688

PET-CT

Name: YEH **Sex:** **Age: 63** **Inspection date: 2024-** **Inspection No.:**

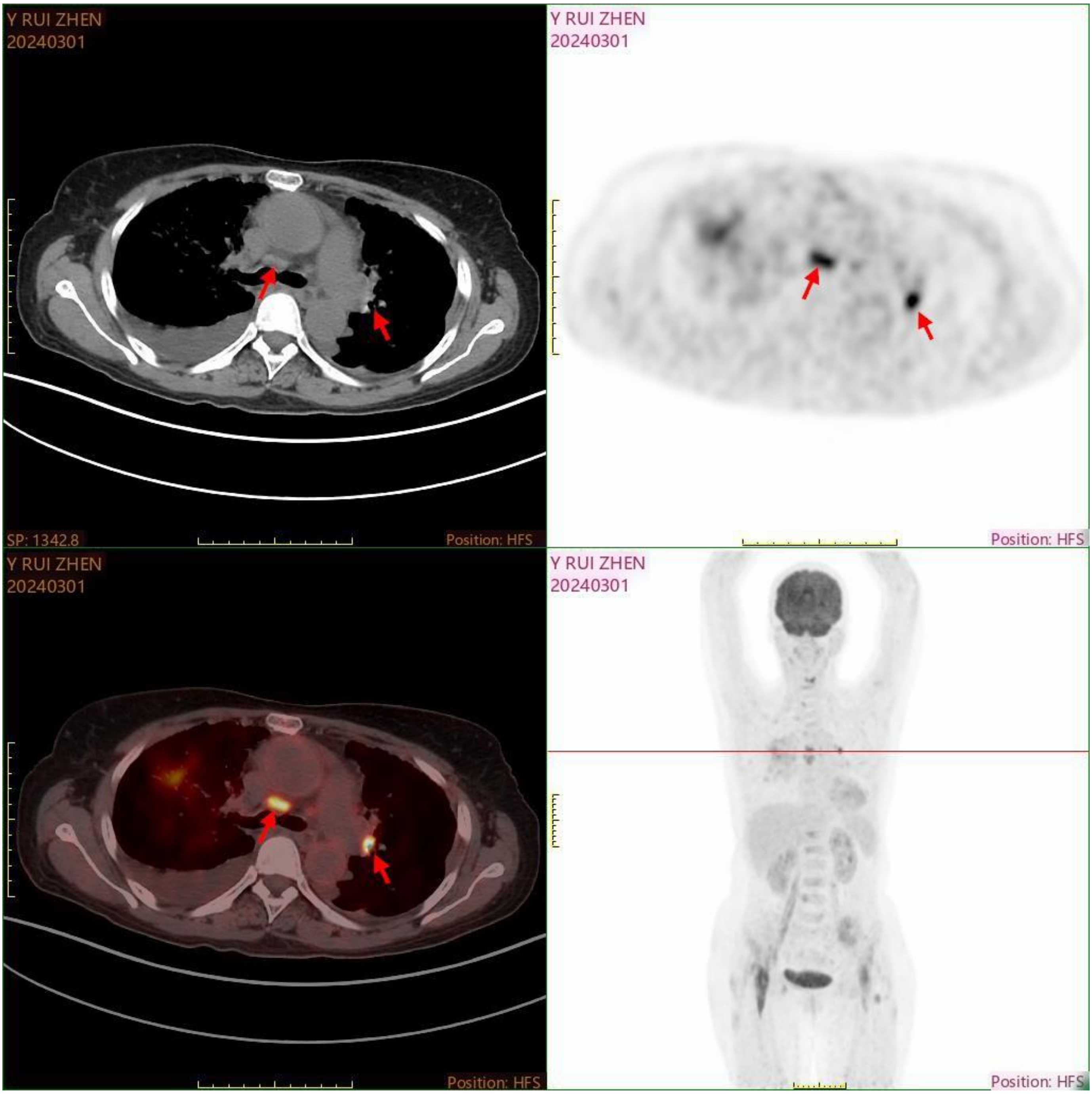

Address:Pavilion 21, No.117 Liuhua Road, Yuexiu Tel: 020-83220688

PET-CT

Name: YEH **Sex:** **Age: 63** **Inspection date: 2024-** **Inspection No.:**

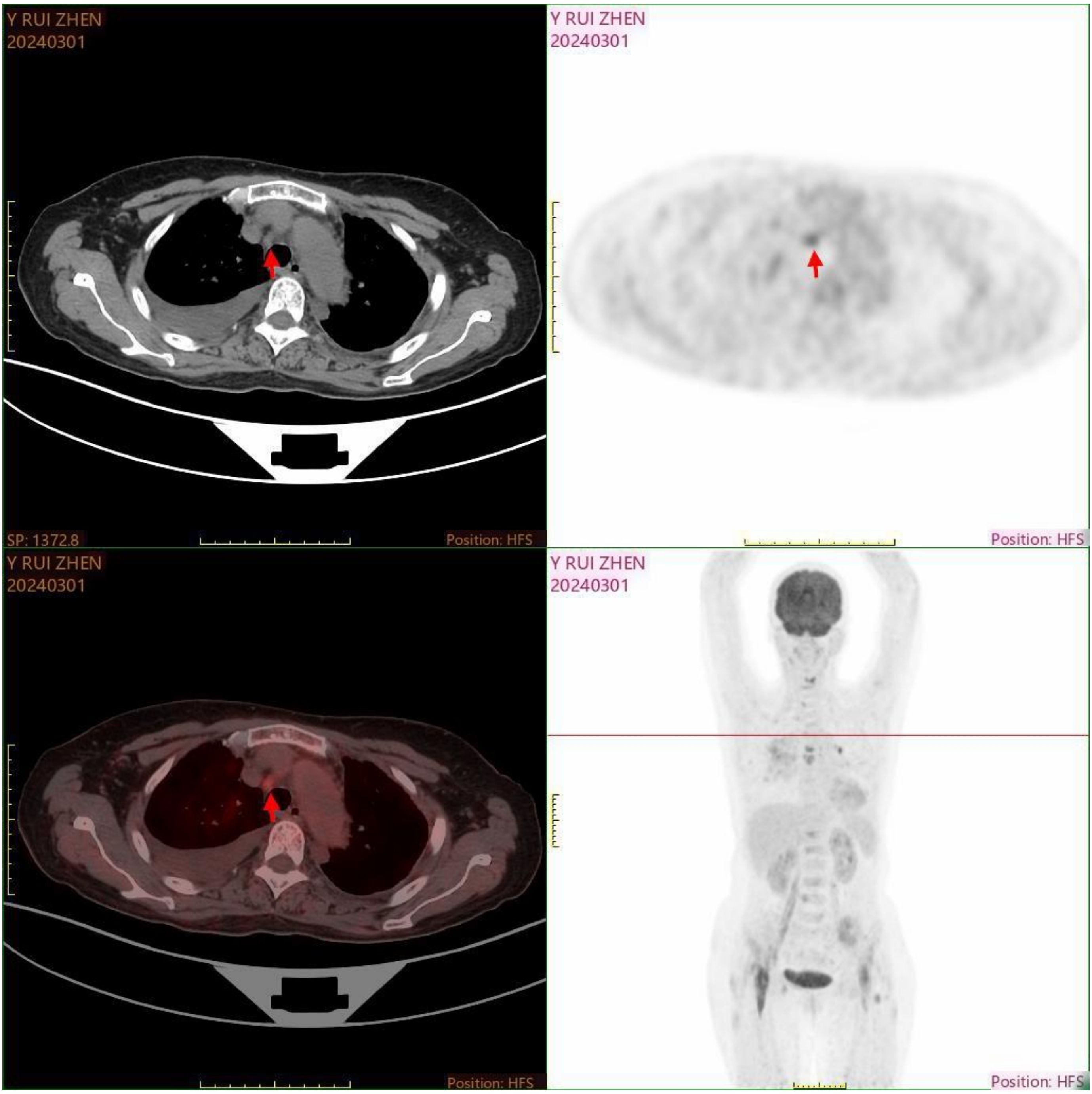

Address:Pavilion 21, No.117 Liuhua Road, Yuexiu Tel: 020-83220688

PET-CT

Name: YEH **Sex:** **Age: 63** **Inspection date: 2024-** **Inspection No.:**

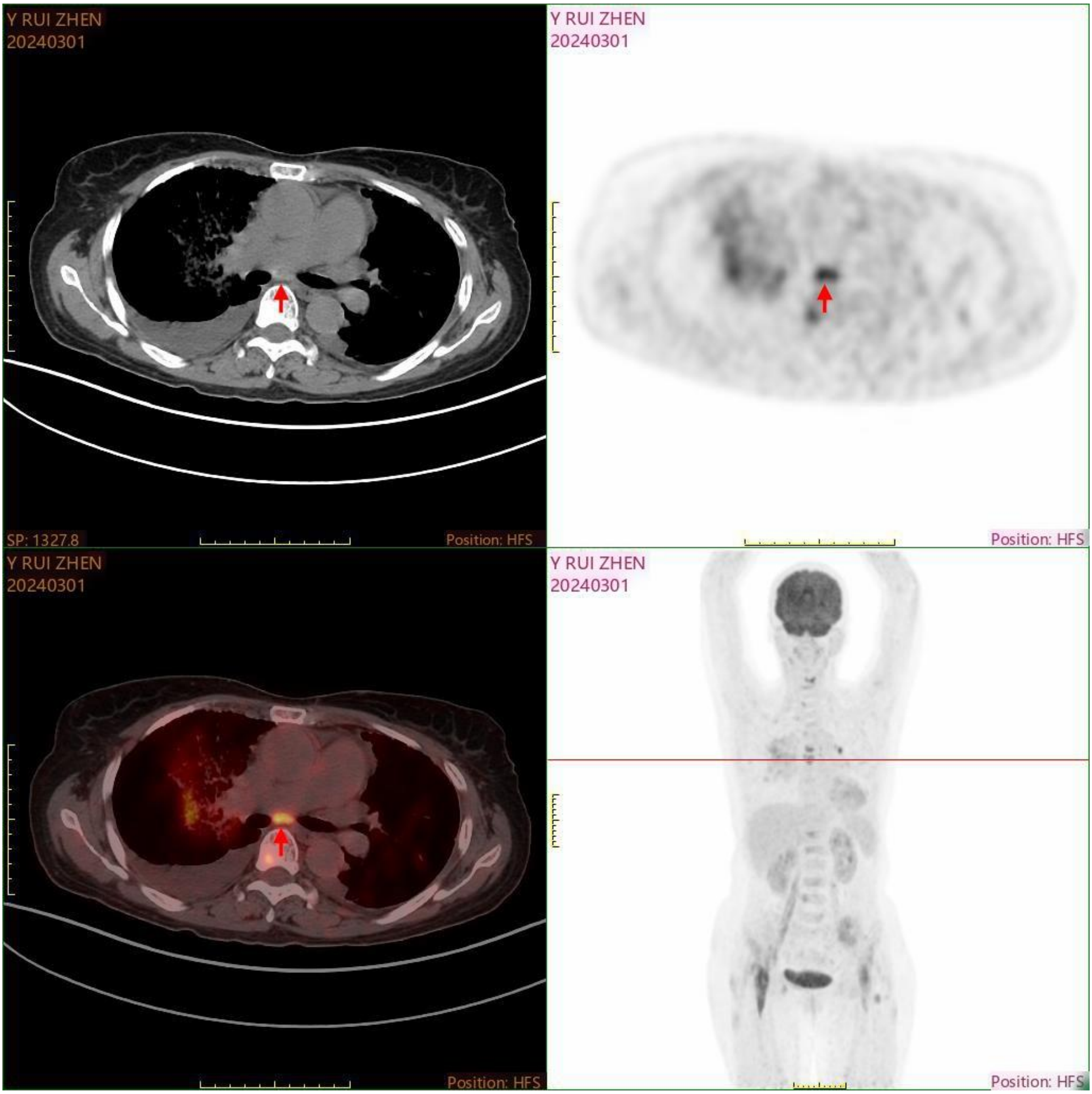

Address:Pavilion 21, No.117 Liuhua Road, Yuexiu Tel: 020-83220688

PET-CT

Name: YEH **Sex:** **Age: 63** **Inspection date: 2024-** **Inspection No.:**

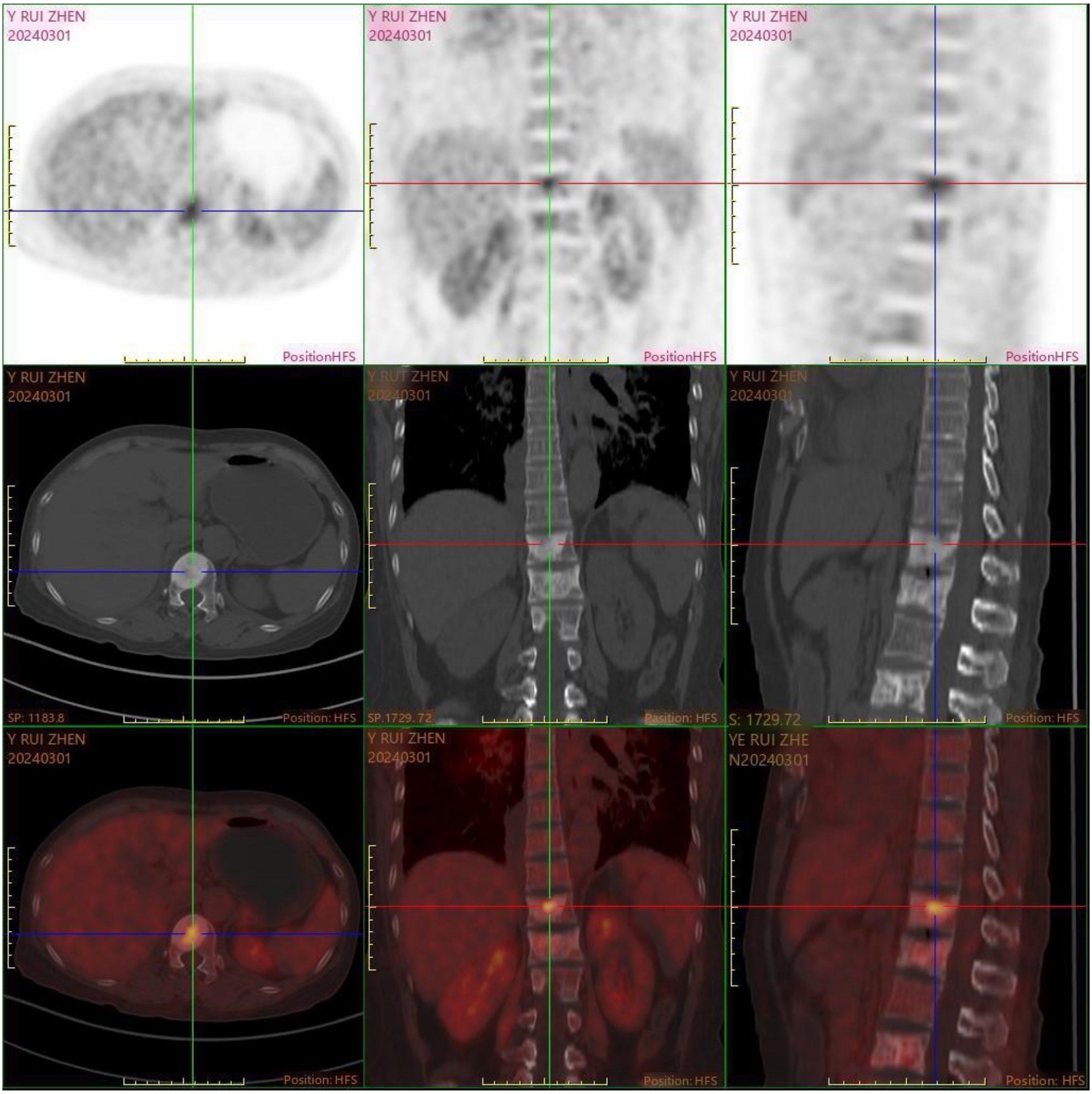

Address:Pavilion 21, No.117 Liuhua Road, Yuexiu Tel: 020-83220688

PET-CT

Name: YEH **Sex:** **Age: 63** **Inspection date: 2024-** **Inspection No.:**

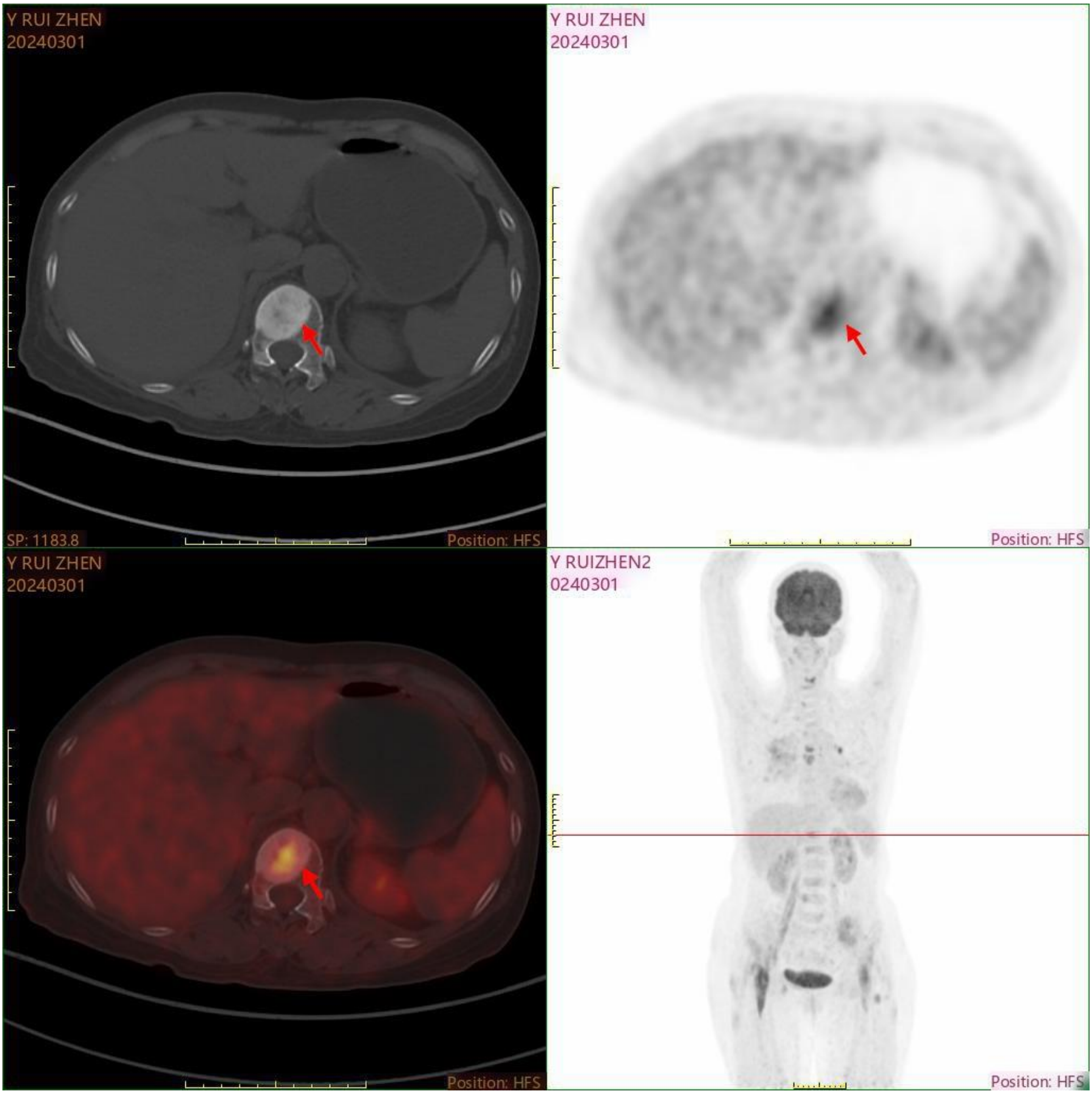

Address:Pavilion 21, No.117 Liuhua Road, Yuexiu Tel: 020-83220688

PET-CT

Name: YEH **Sex:** **Age: 63** **Inspection date: 2024-** **Inspection No.:**

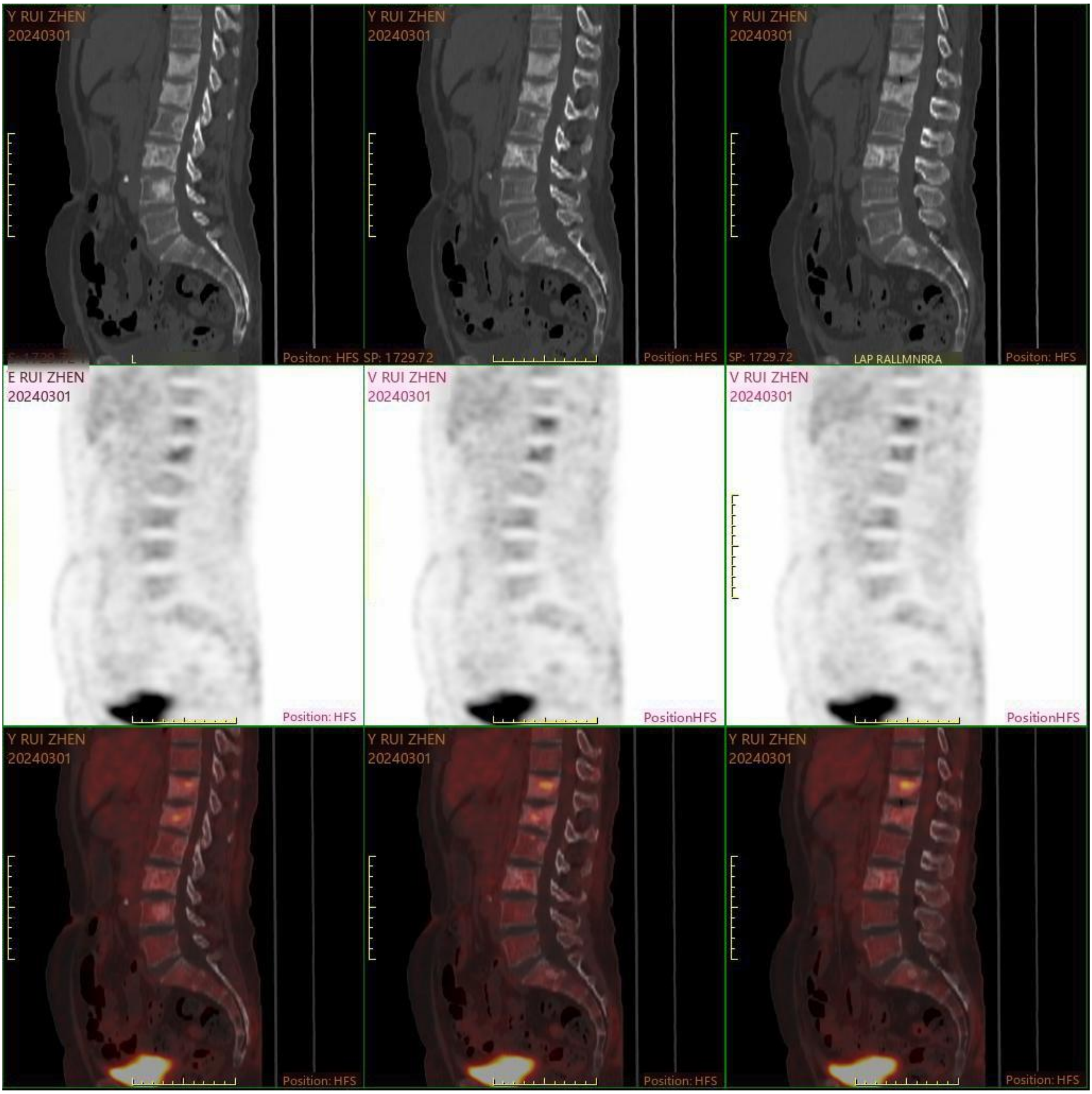

Address:Pavilion 21, No.117 Liuhua Road, Yuexiu Tel: 020-83220688

PET-CT

Name: YEH **Sex:** **Age: 63** **Inspection date: 2024-** **Inspection No.:**

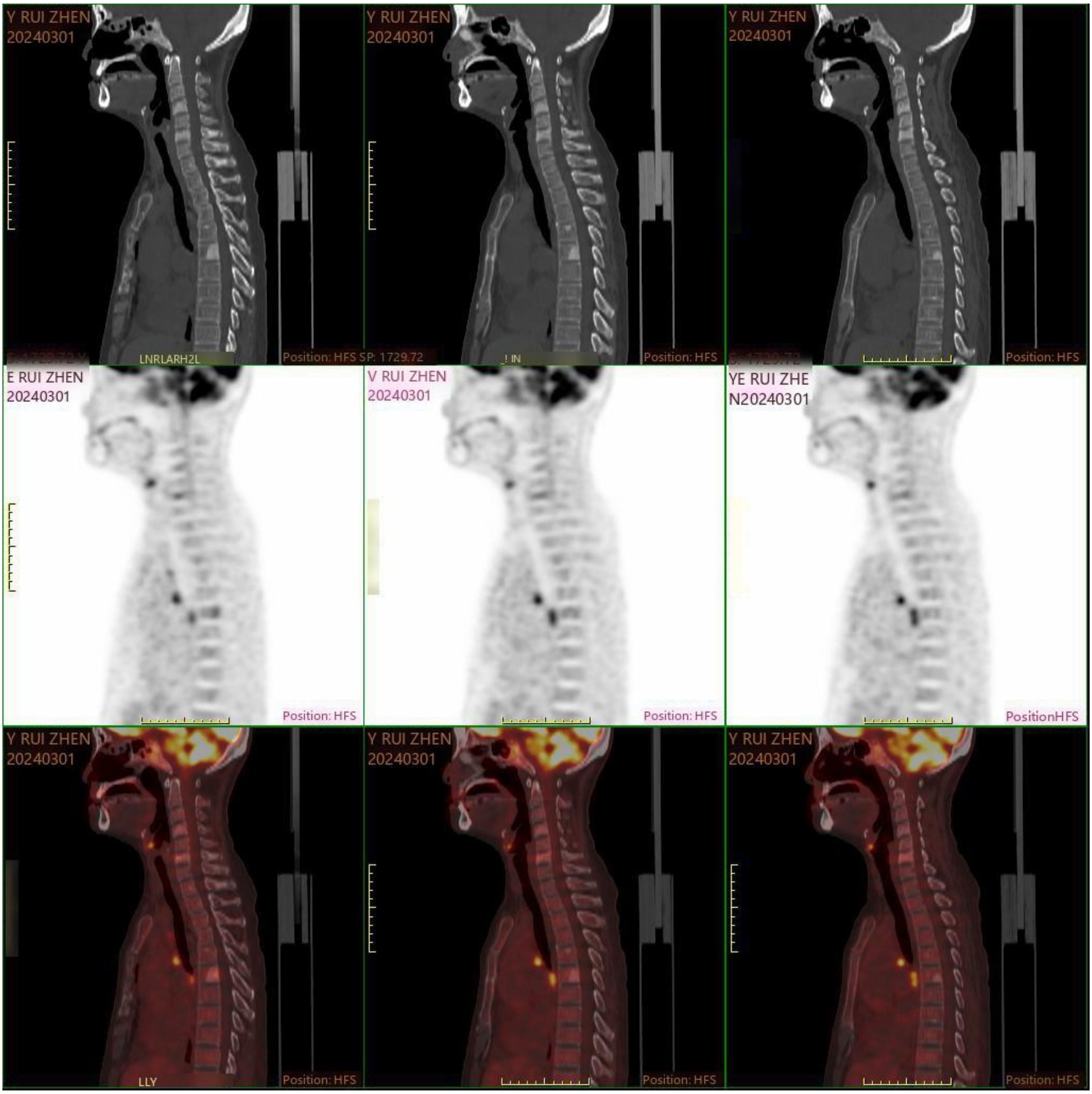

Address:Pavilion 21, No.117 Liuhua Road, Yuexiu Tel: 020-83220688

PET-CT

Name: YEH **Sex:** **Age: 63** **Inspection date: 2024-** **Inspection No.:**

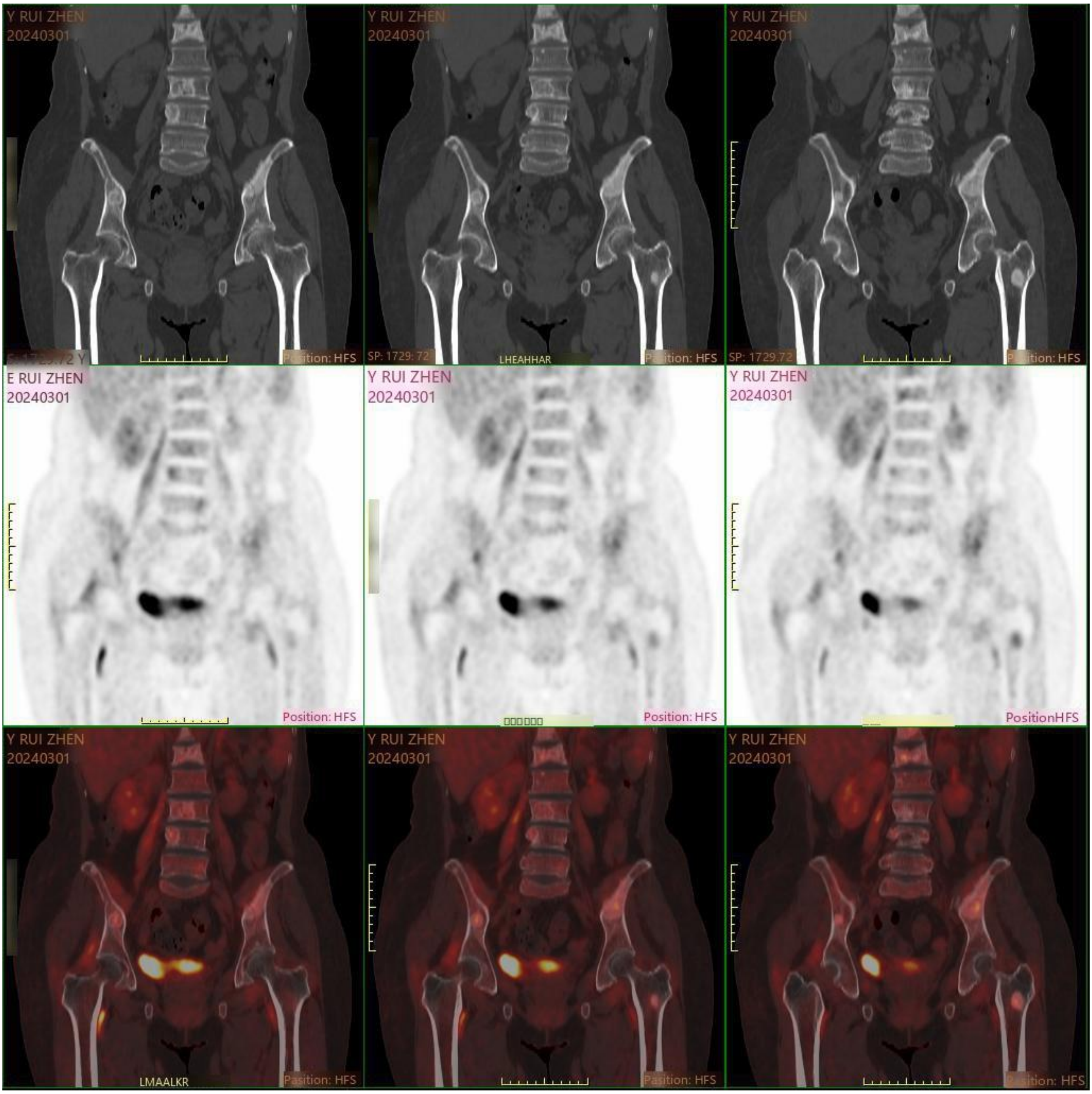

Address:Pavilion 21, No.117 Liuhua Road, Yuexiu Tel: 020-83220688

PET-CT

Name: YEH **Sex:** **Age: 63** **Inspection date: 2024-** **Inspection No.:**

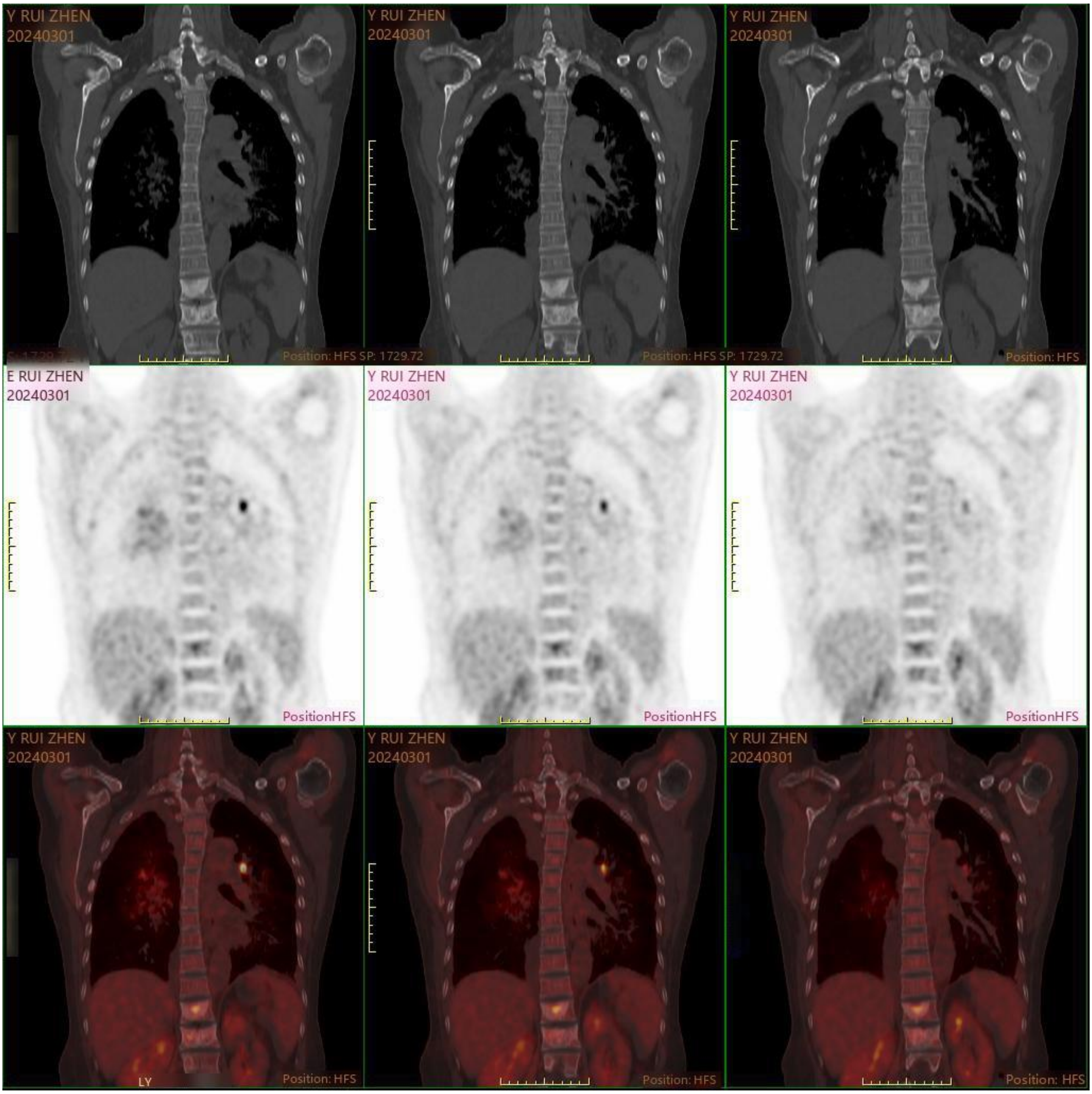

PET-CT

Name: YEH **Sex:** **Age: 63** **Inspection date: 2024-** **Inspection No.:**

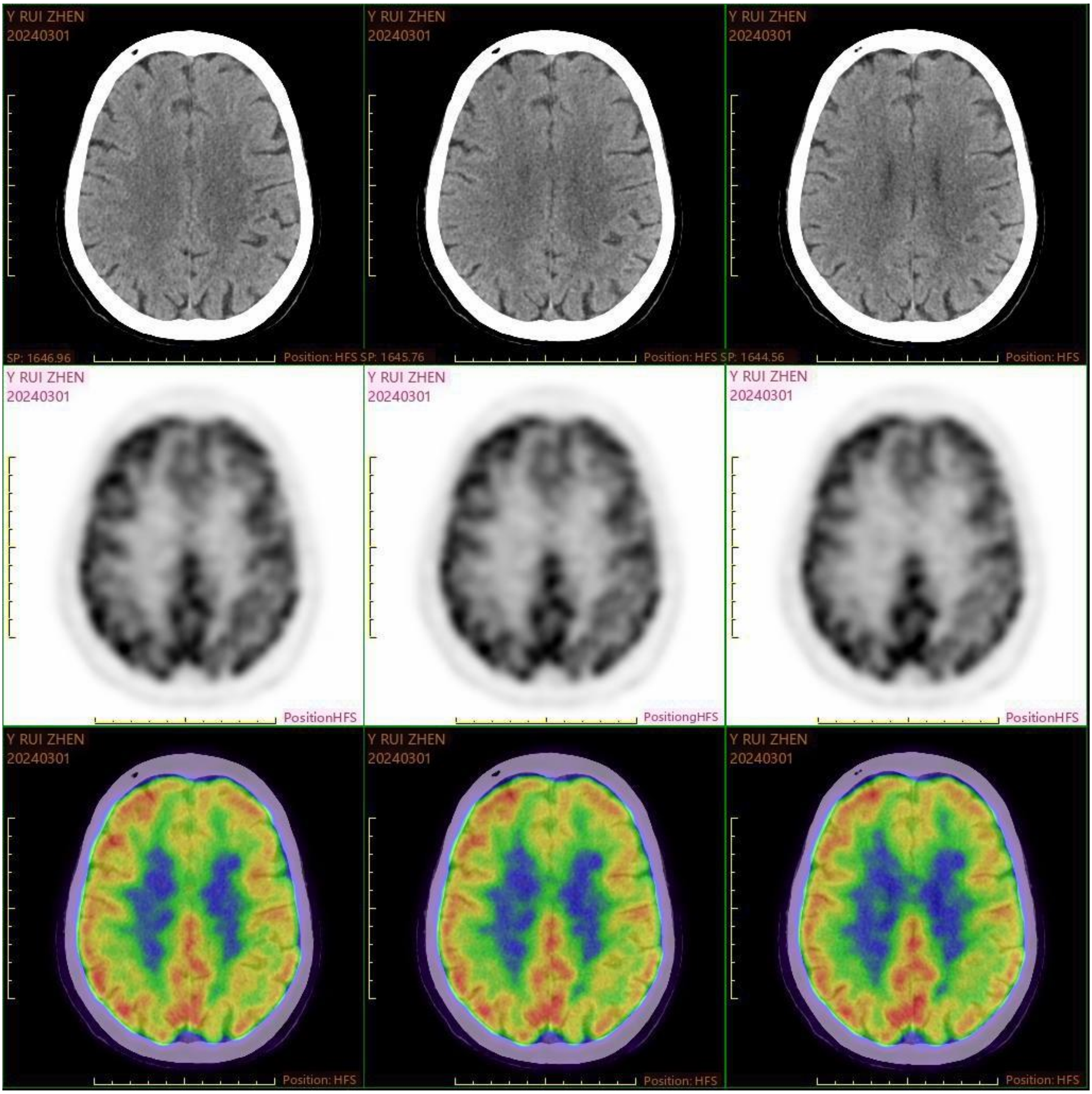

PET-CT

Name: YEH **Sex:** **Age: 63** **Inspection date: 2024-** **Inspection No.:**

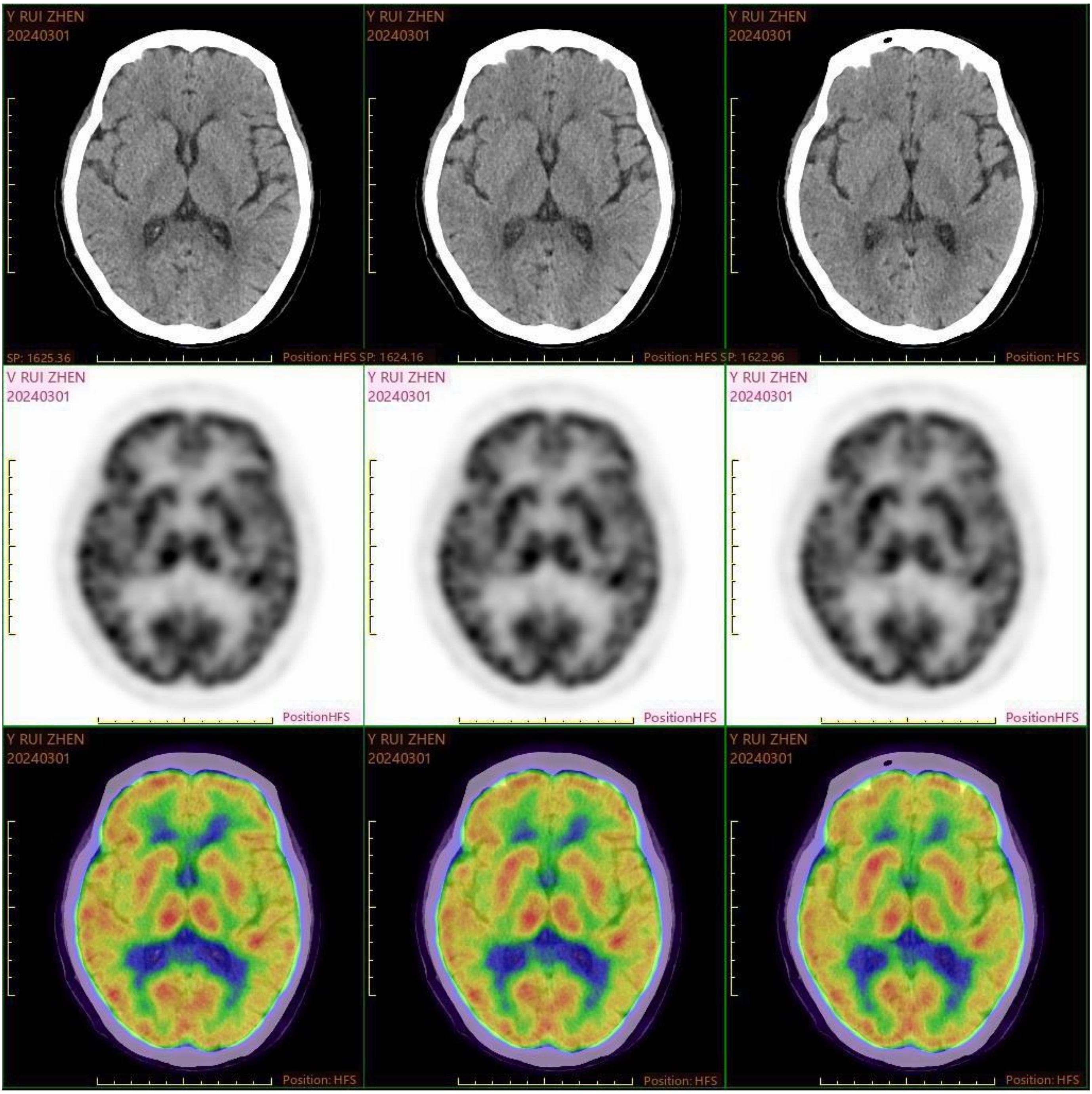

PET-CT

Name: YEH **Sex:** **Age: 63** **Inspection date: 2024-** **Inspection No.:**

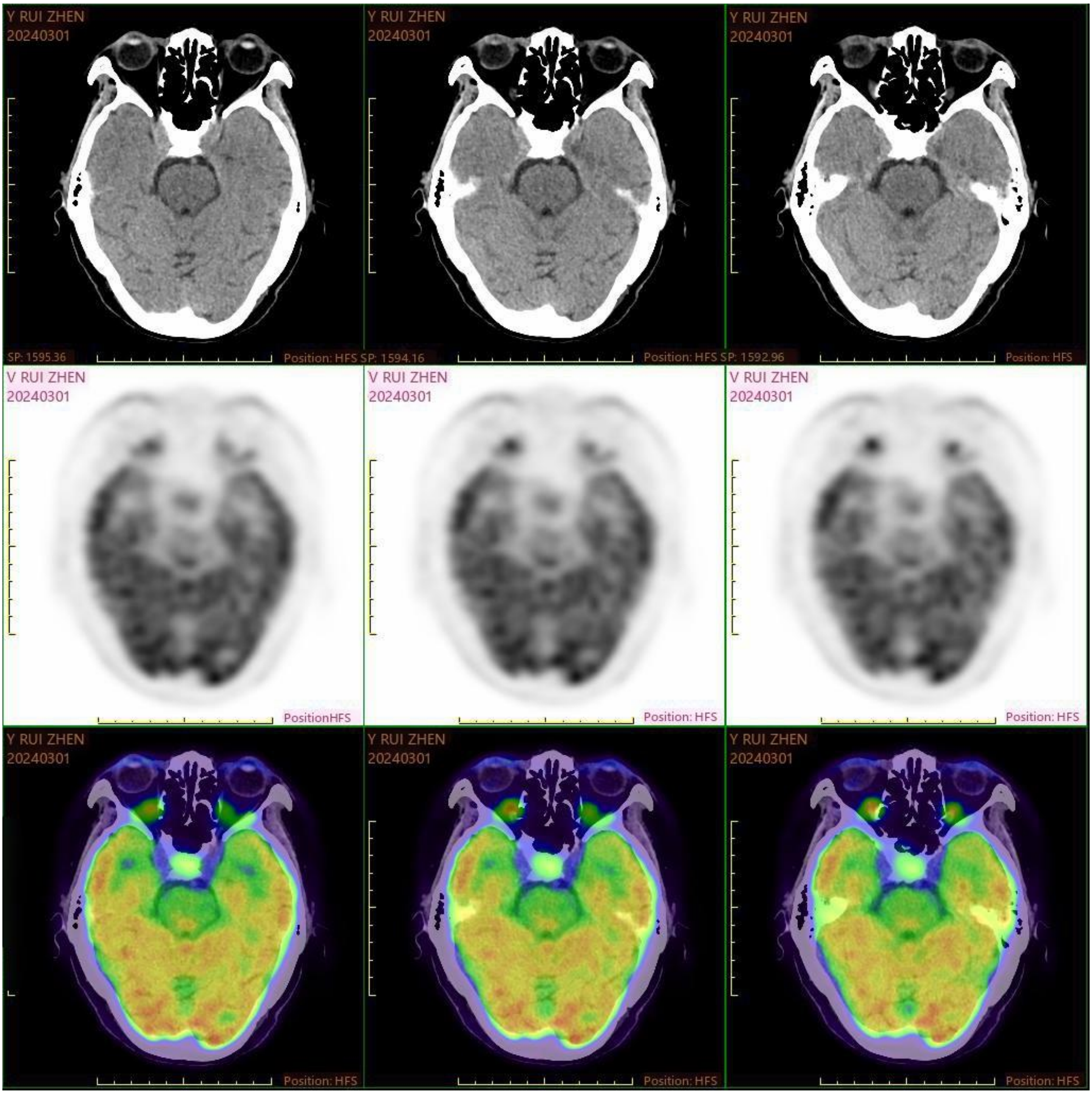

Address:Pavilion 21, No.117 Liuhua Road, Yuexiu Tel: 020-83220688

PET-CT

Name: YEH **Sex:** **Age: 63** **Inspection date: 2024-** **Inspection No.:**

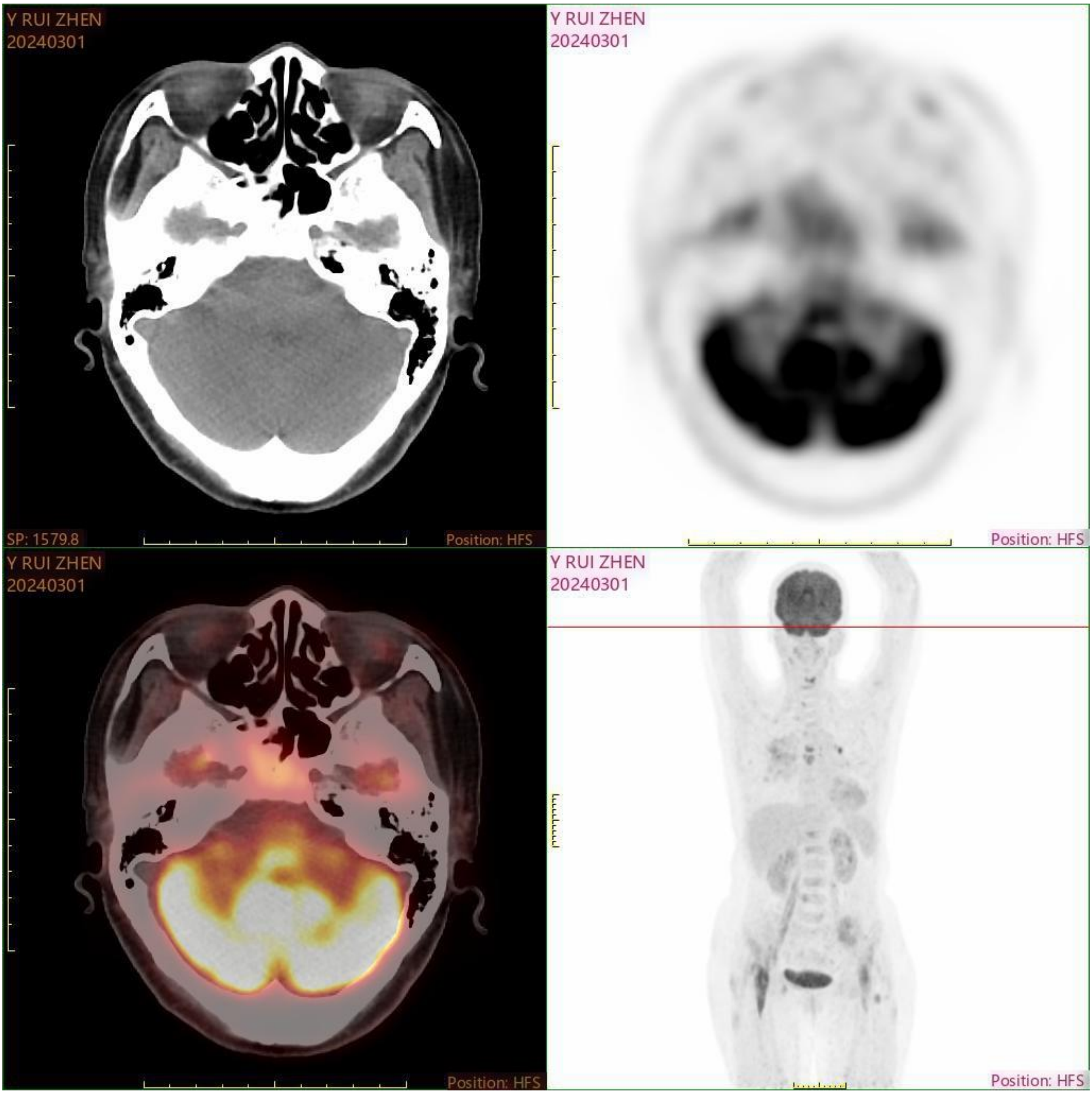

Address:Pavilion 21, No.117 Liuhua Road, Yuexiu Tel: 020-83220688

PET-CT

Name: YEH **Sex:** **Age: 63** **Inspection date: 2024-** **Inspection No.:**

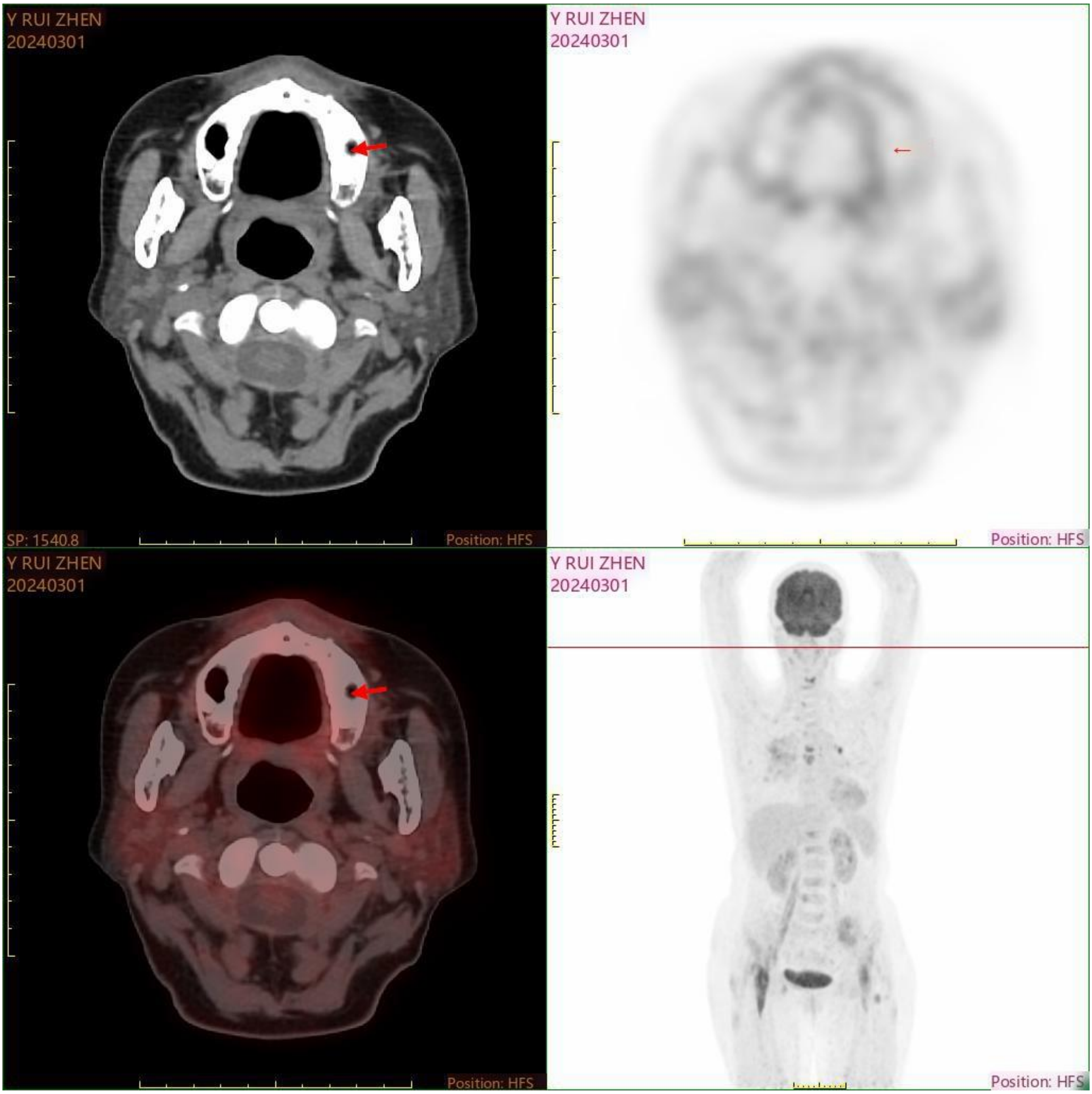

Address:Pavilion 21, No.117 Liuhua Road, Yuexiu Tel: 020-83220688

PET-CT

Name: YEH **Sex:** **Age: 63** **Inspection date: 2024-** **Inspection No.:**

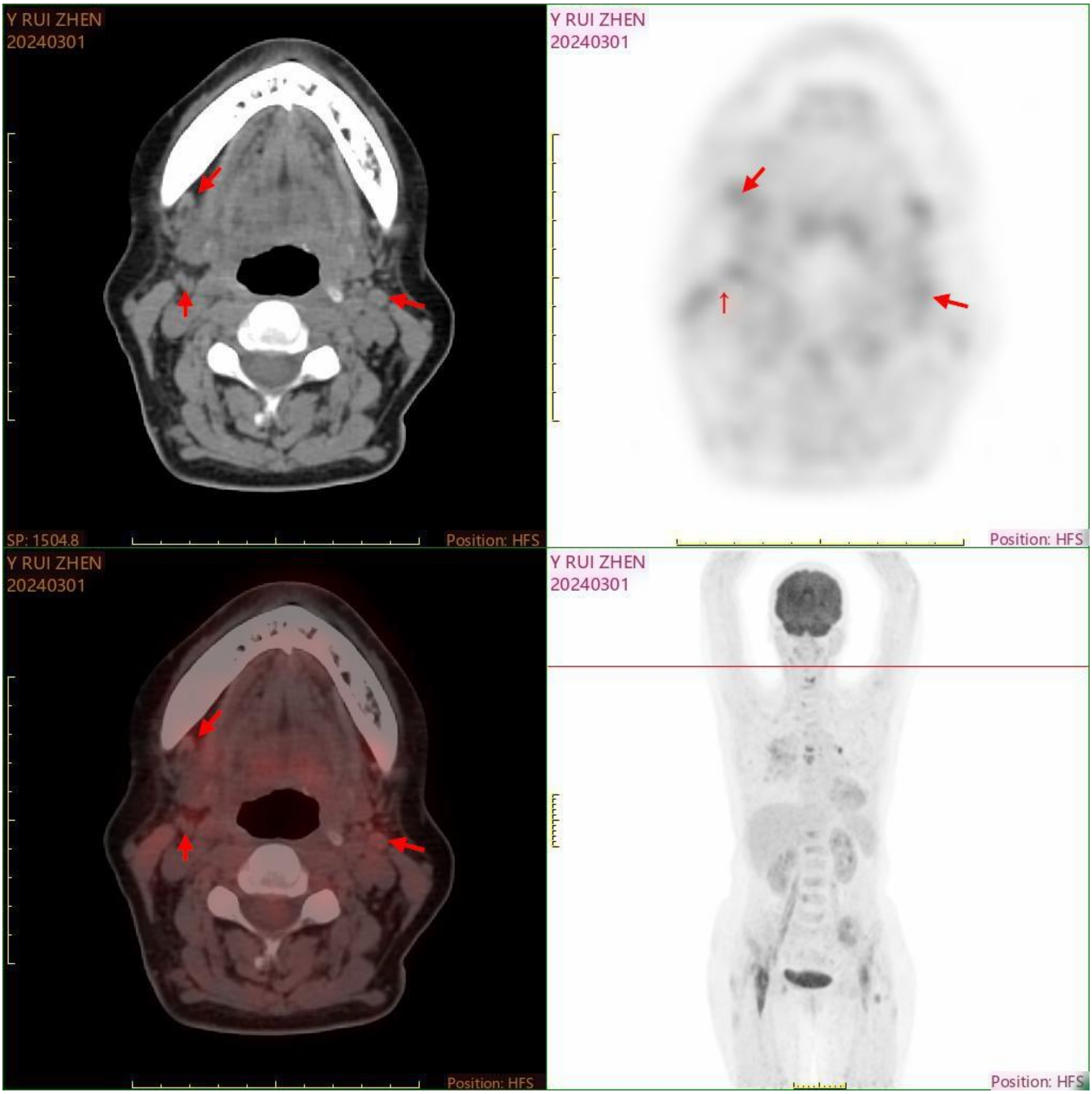

Address:Pavilion 21, No.117 Liuhua Road, Yuexiu
Tel: 020-83220688

PET-CT

Name: YEH **Sex:** **Age: 63** **Inspection date: 2024-** **Inspection No.:**

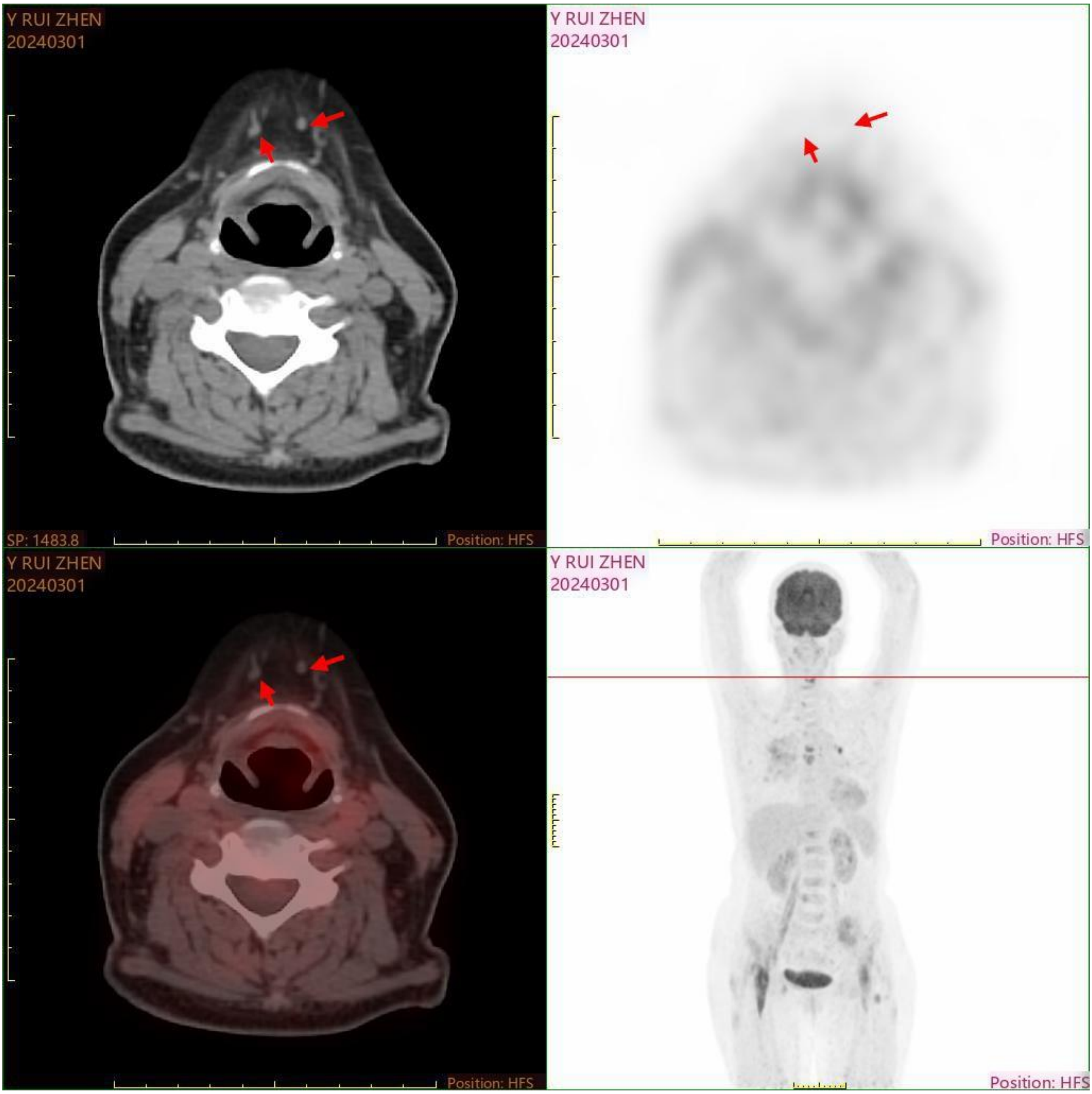

PET-CT

Name: YEH **Sex:** **Age: 63** **Inspection date: 2024-** **Inspection No.:**

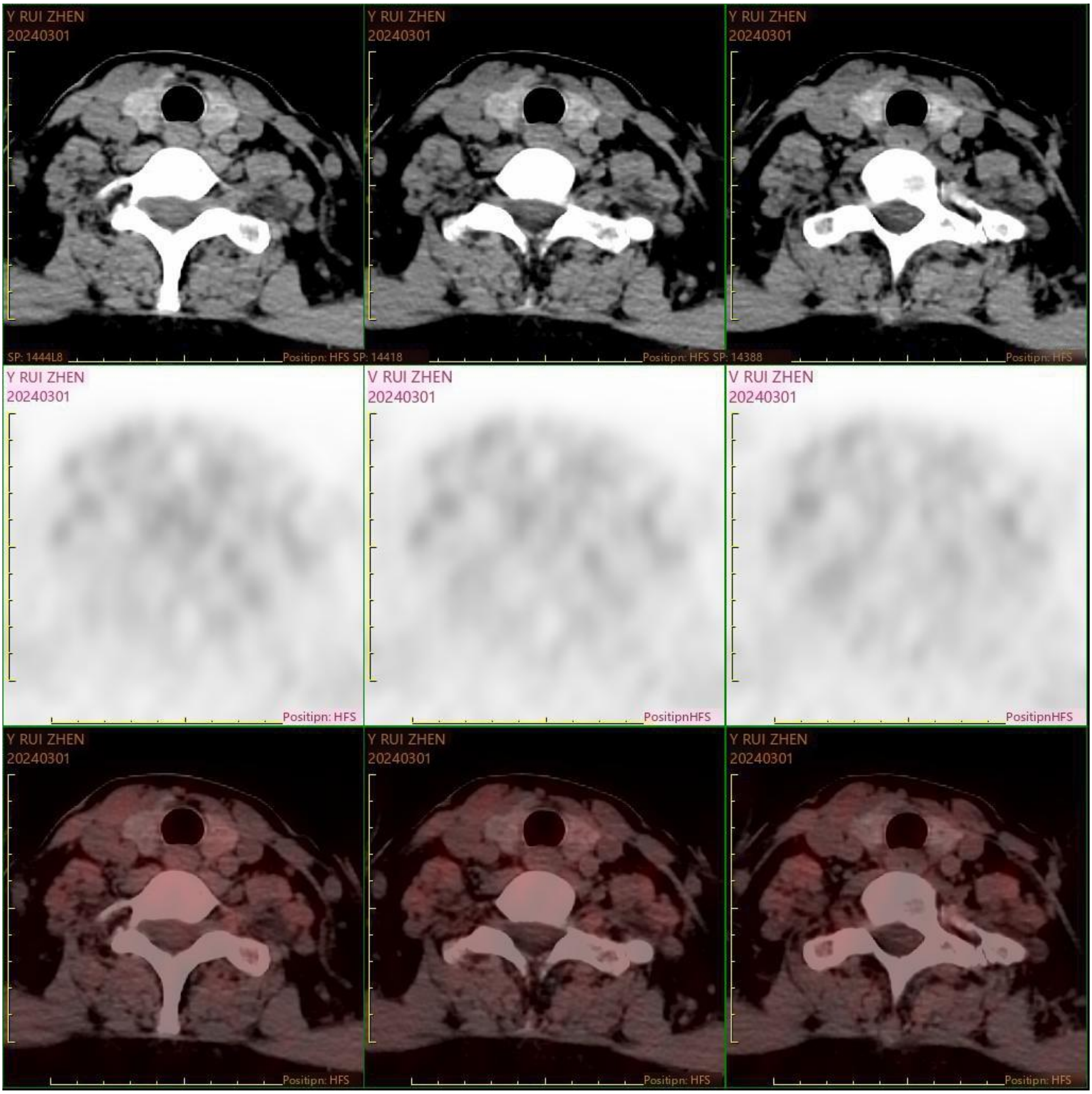

Address:Pavilion 21, No.117 Liuhua Road, Yuexiu Tel: 020-83220688

PET-CT

Name: YEH **Sex:** **Age: 63** **Inspection date: 2024-** **Inspection No.:**

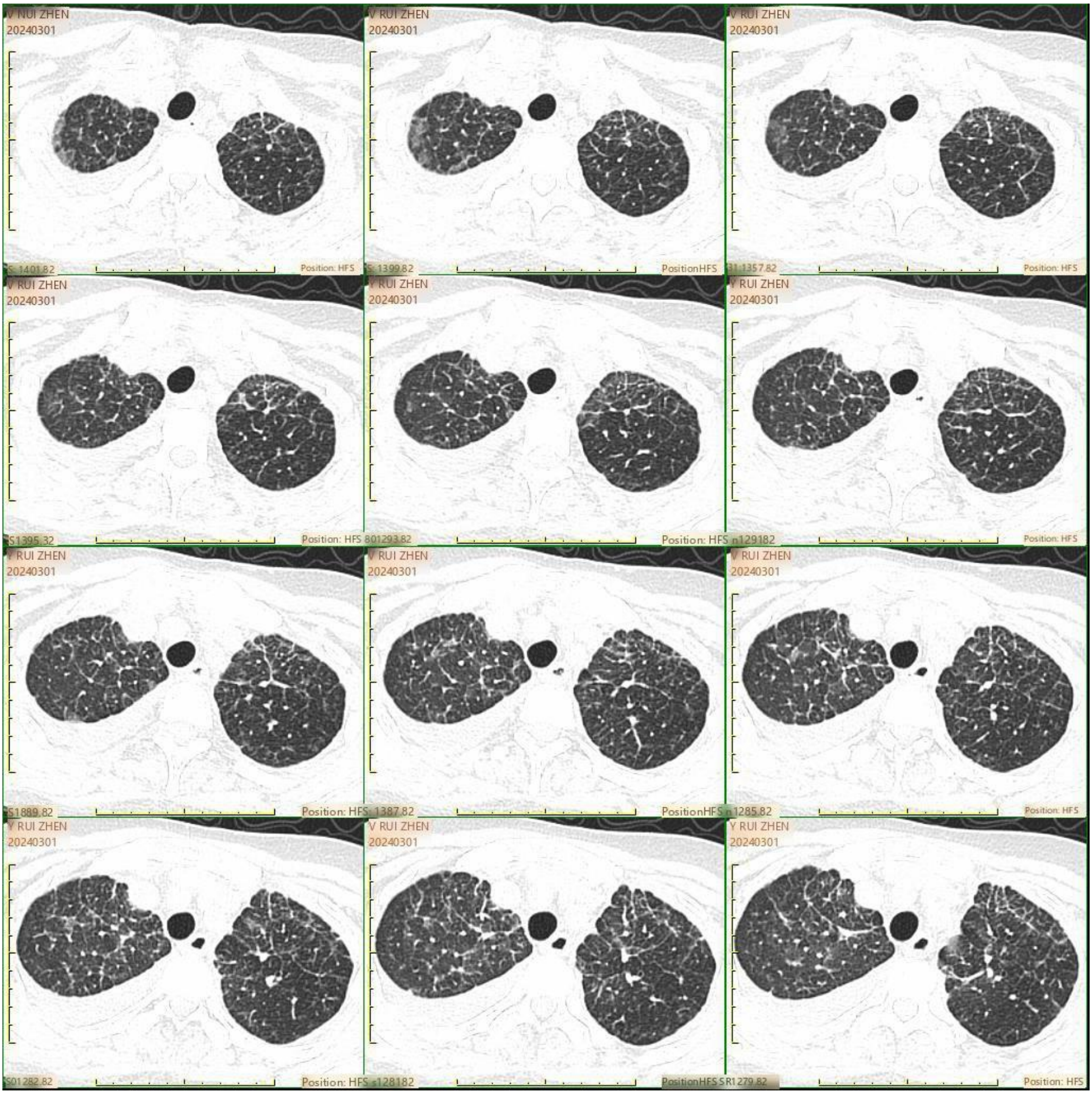

Address:Pavilion 21, No.117 Liuhua Road, Yuexiu
Tel: 020-83220688

PET-CT

Name: YEH **Sex:** **Age: 63** **Inspection date: 2024-** **Inspection No.:**

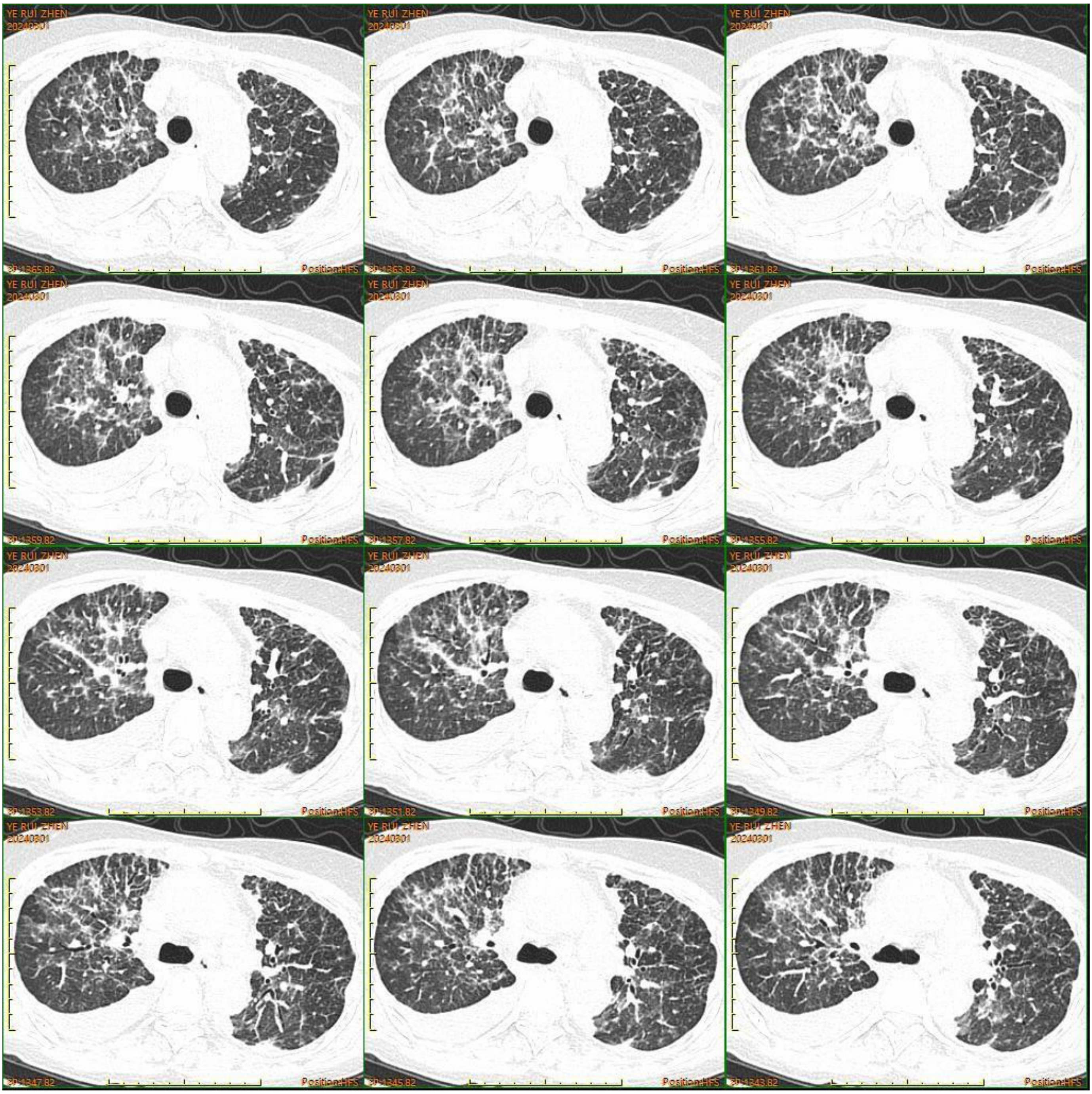

Address:Pavilion 21, No.117 Liuhua Road, Yuexiu Tel: 020-83220688

PET-CT

Name: YEH **Sex:** **Age: 63** **Inspection date: 2024-** **Inspection No.:**

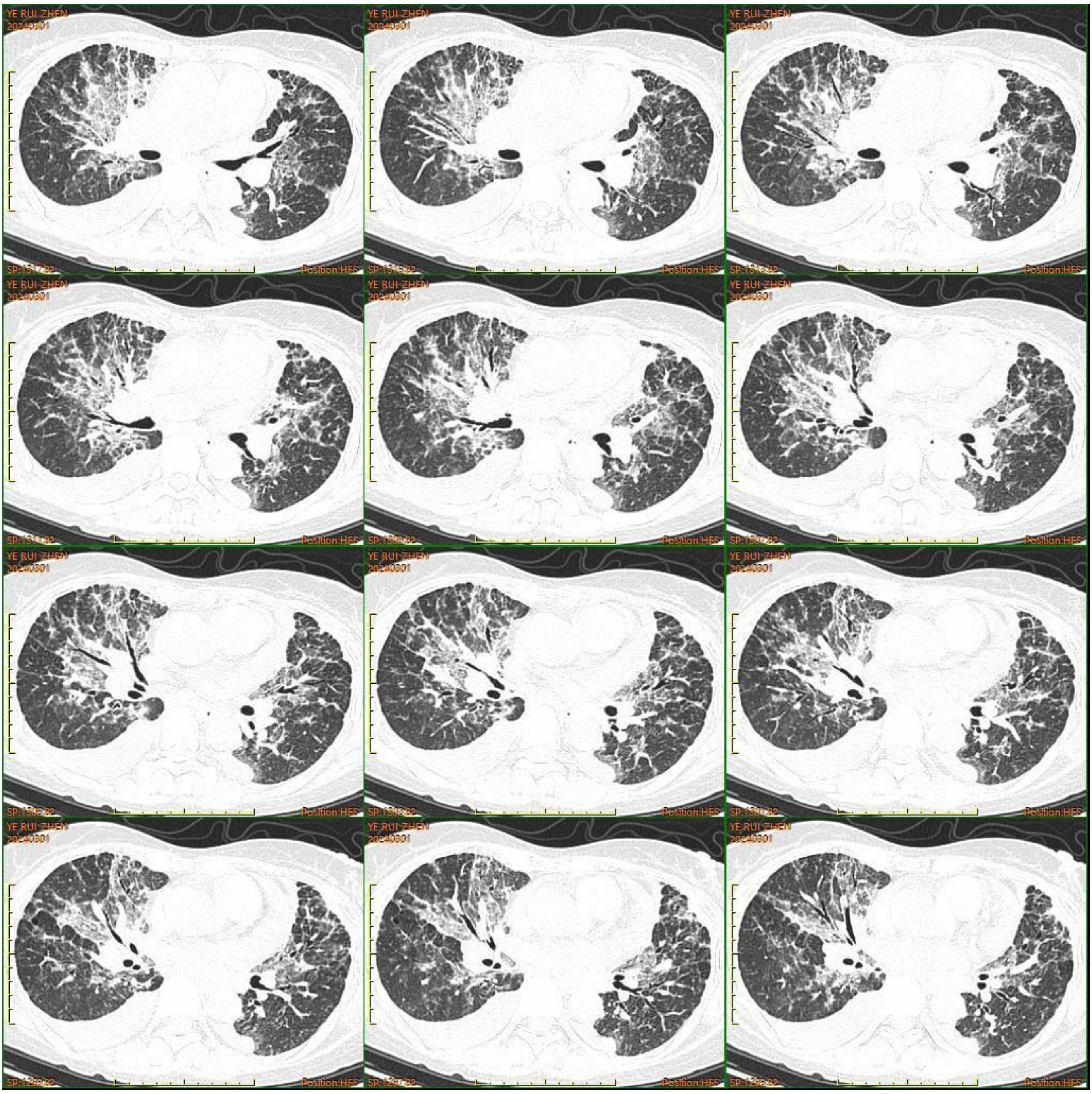

Address:Pavilion 21, No.117 Liuhua Road, Yuexiu
Tel: 020-83220688

PET-CT

Name: YEH **Sex:** **Age: 63** **Inspection date: 2024-** **Inspection No.:**

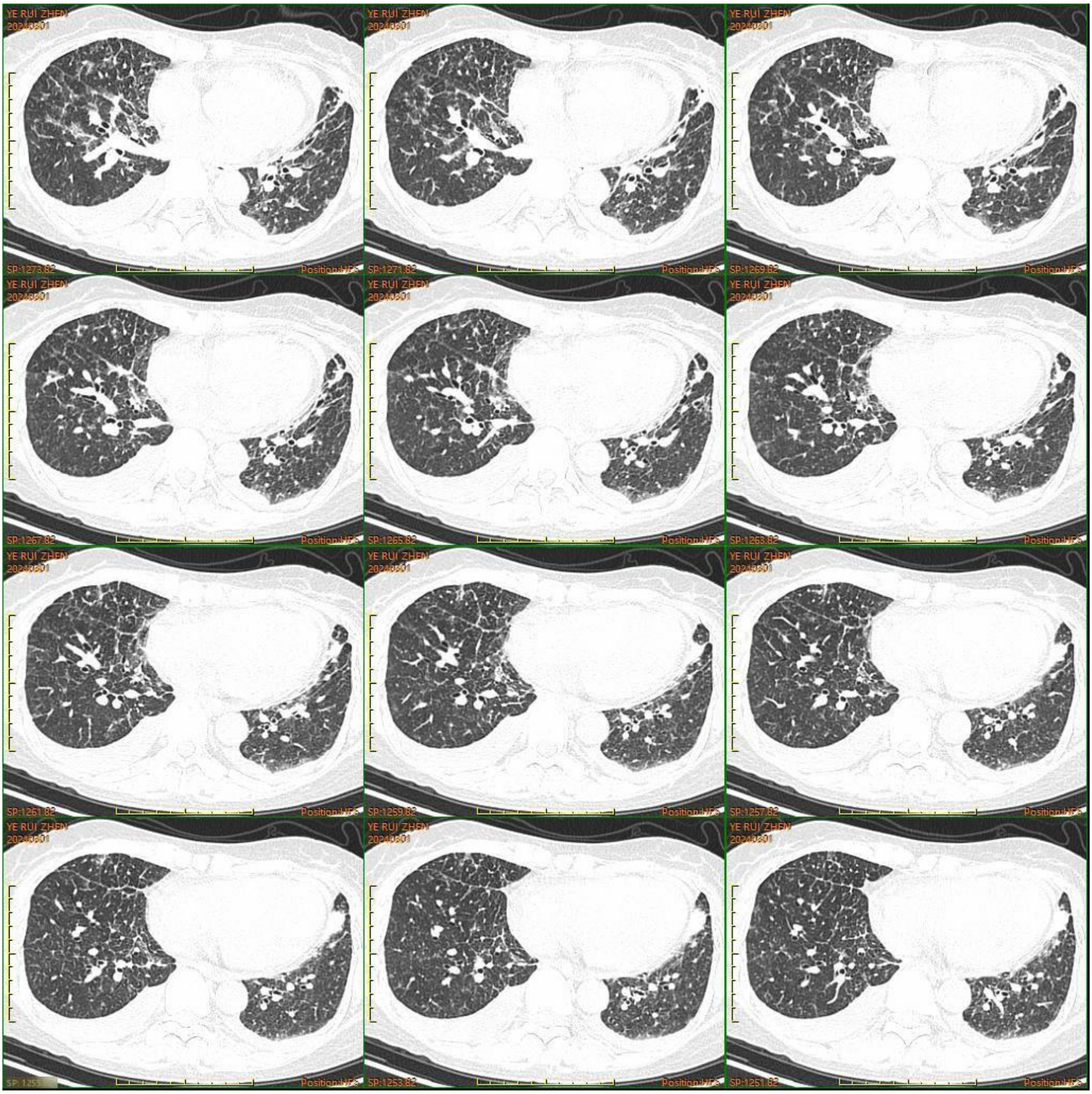

Address:Pavilion 21, No.117 Liuhua Road, Yuexiu Tel: 020-83220688

PET-CT

Name: YEH **Sex:** **Age: 63** **Inspection date: 2024-** **Inspection No.:**

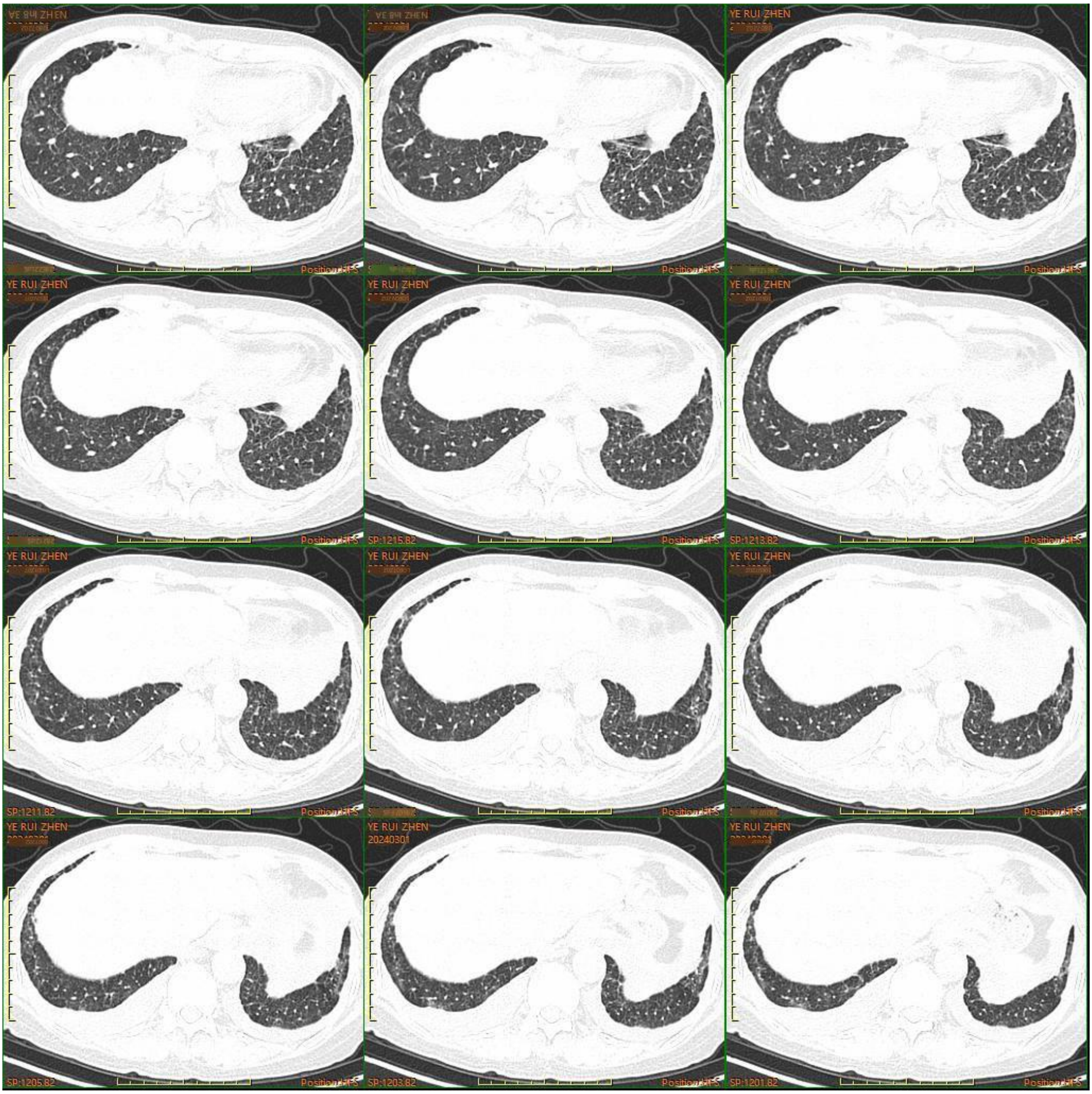

Address:Pavilion 21, No.117 Liuhua Road, Yuexiu
Tel: 020-83220688

PET-CT

Name: YEH **Sex:** **Age: 63** **Inspection date: 2024-** **Inspection No.:**

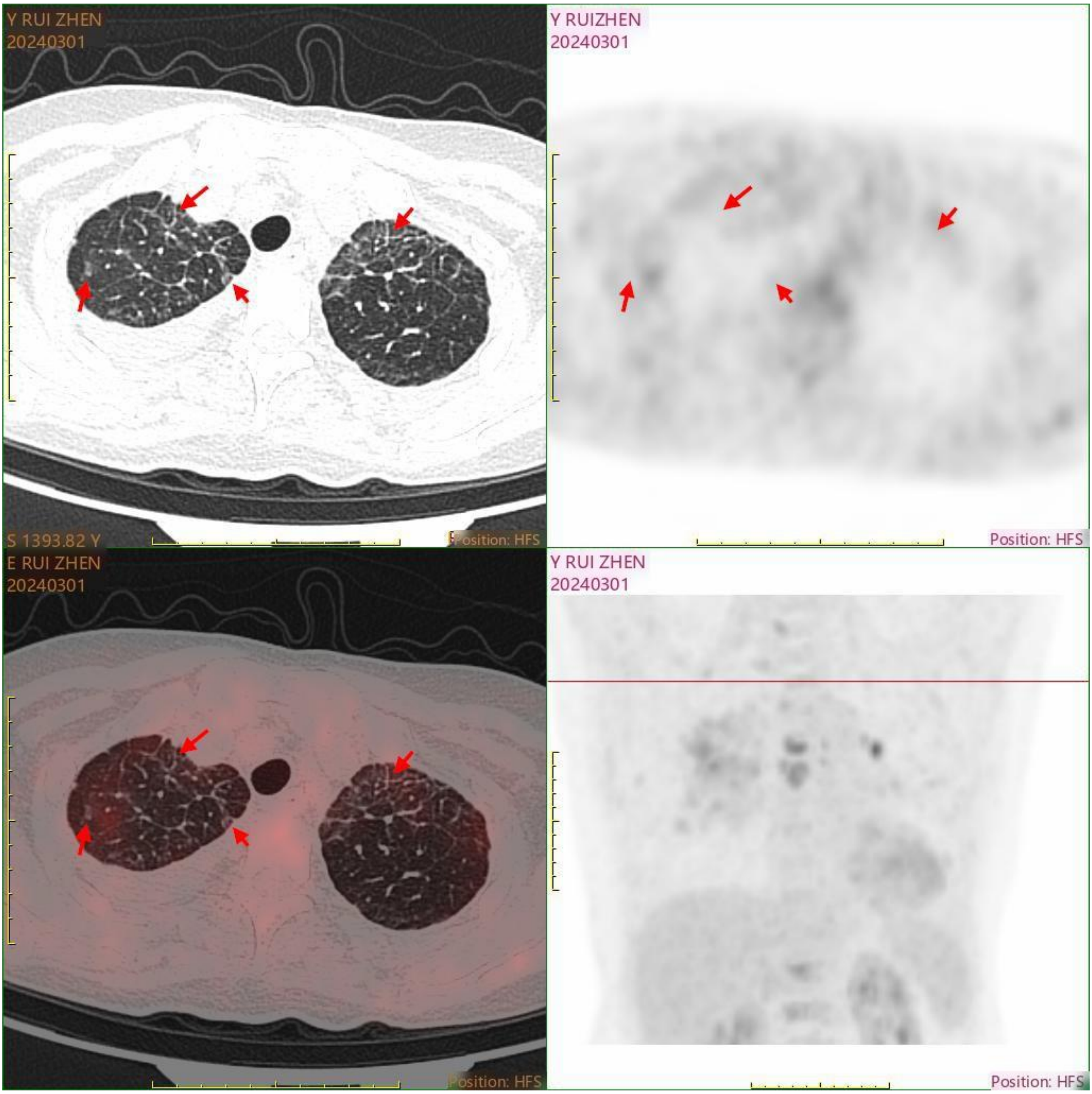

Address:Pavilion 21, No.117 Liuhua Road, Yuexiu
Tel: 020-83220688

PET-CT

Name: YEH **Sex:** **Age: 63** **Inspection date: 2024-** **Inspection No.:**

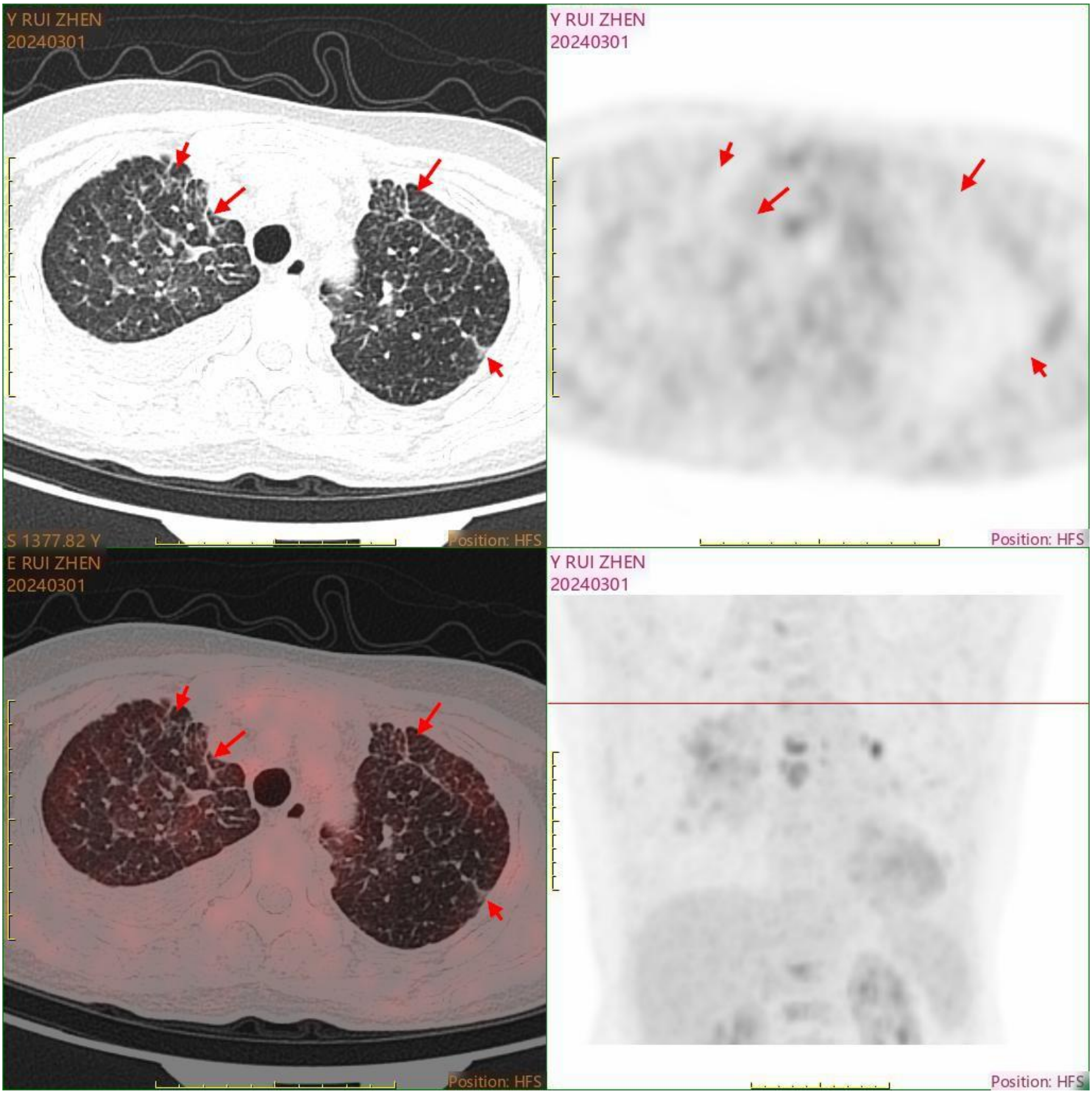

Address:Pavilion 21, No.117 Liuhua Road, Yuexiu Tel: 020-83220688

PET-CT

Name: YEH **Sex:** **Age: 63** **Inspection date: 2024-** **Inspection No.:**

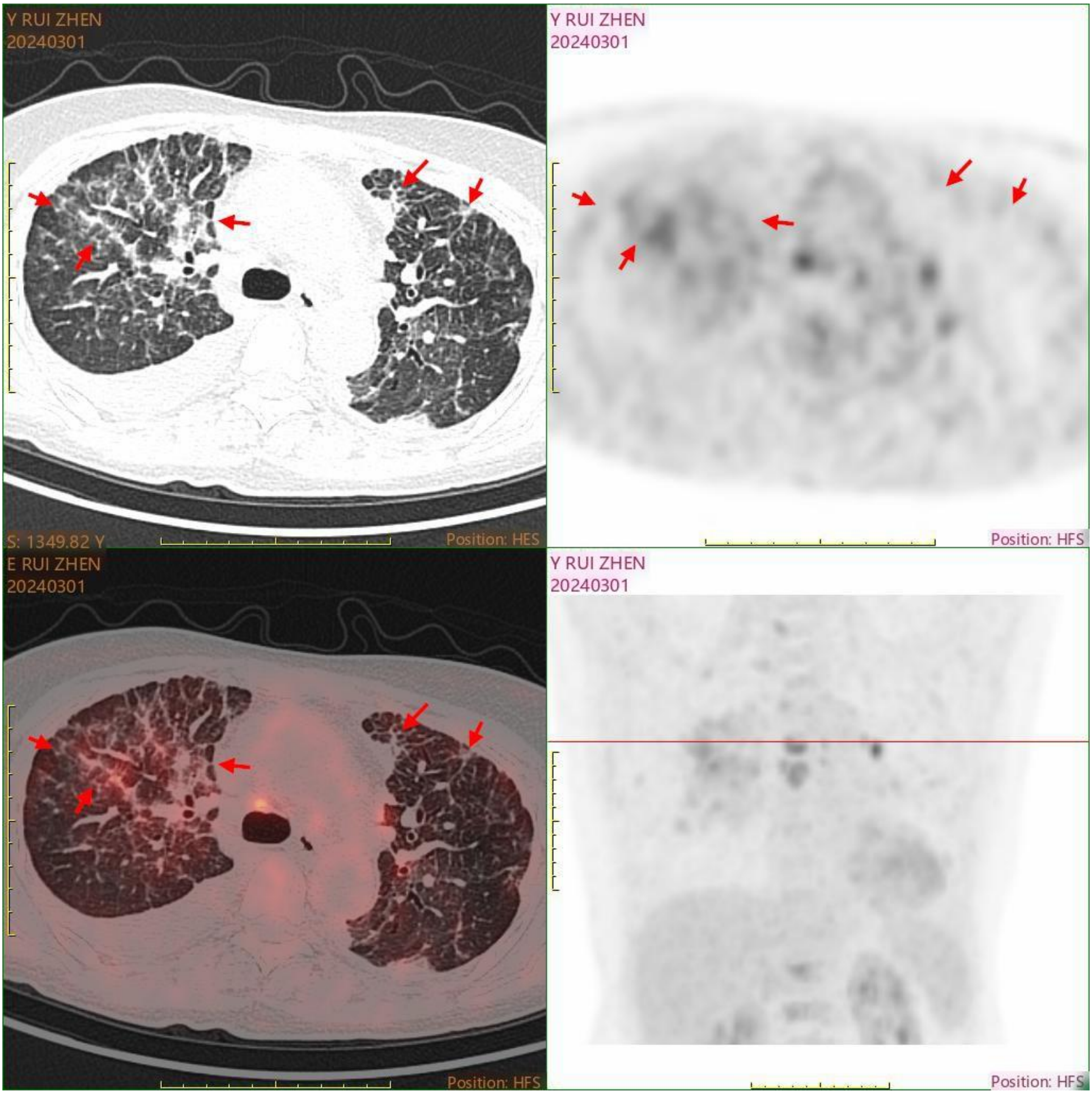

Address:Pavilion 21, No.117 Liuhua Road, Yuexiu
Tel: 020-83220688

PET-CT

Name: YEH **Sex:** **Age: 63** **Inspection date: 2024-** **Inspection No.:**

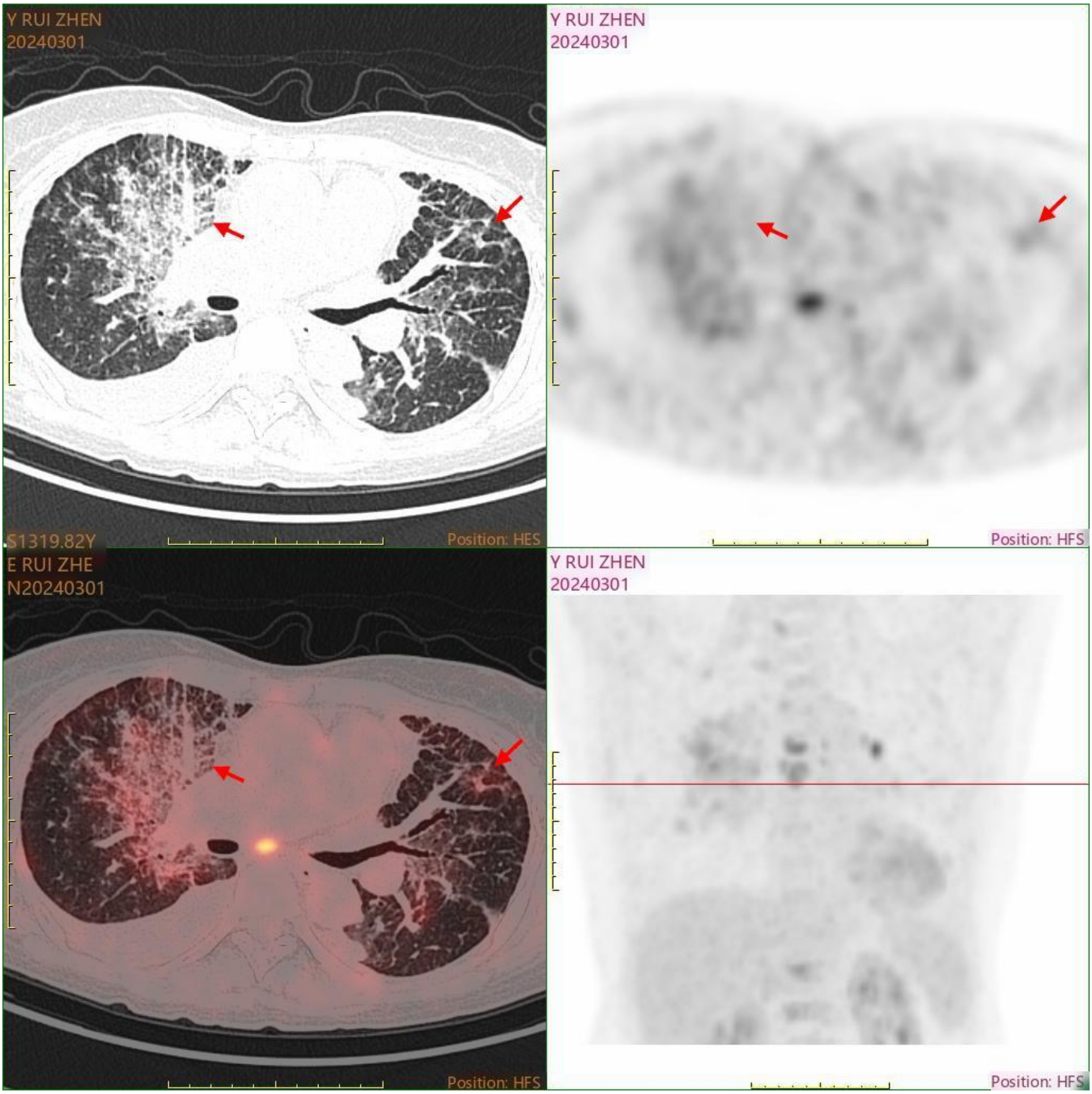

Address:Pavilion 21, No.117 Liuhua Road, Yuexiu Tel: 020-83220688

PET-CT

Name: YEH **Sex:** **Age: 63** **Inspection date: 2024-** **Inspection No.:**

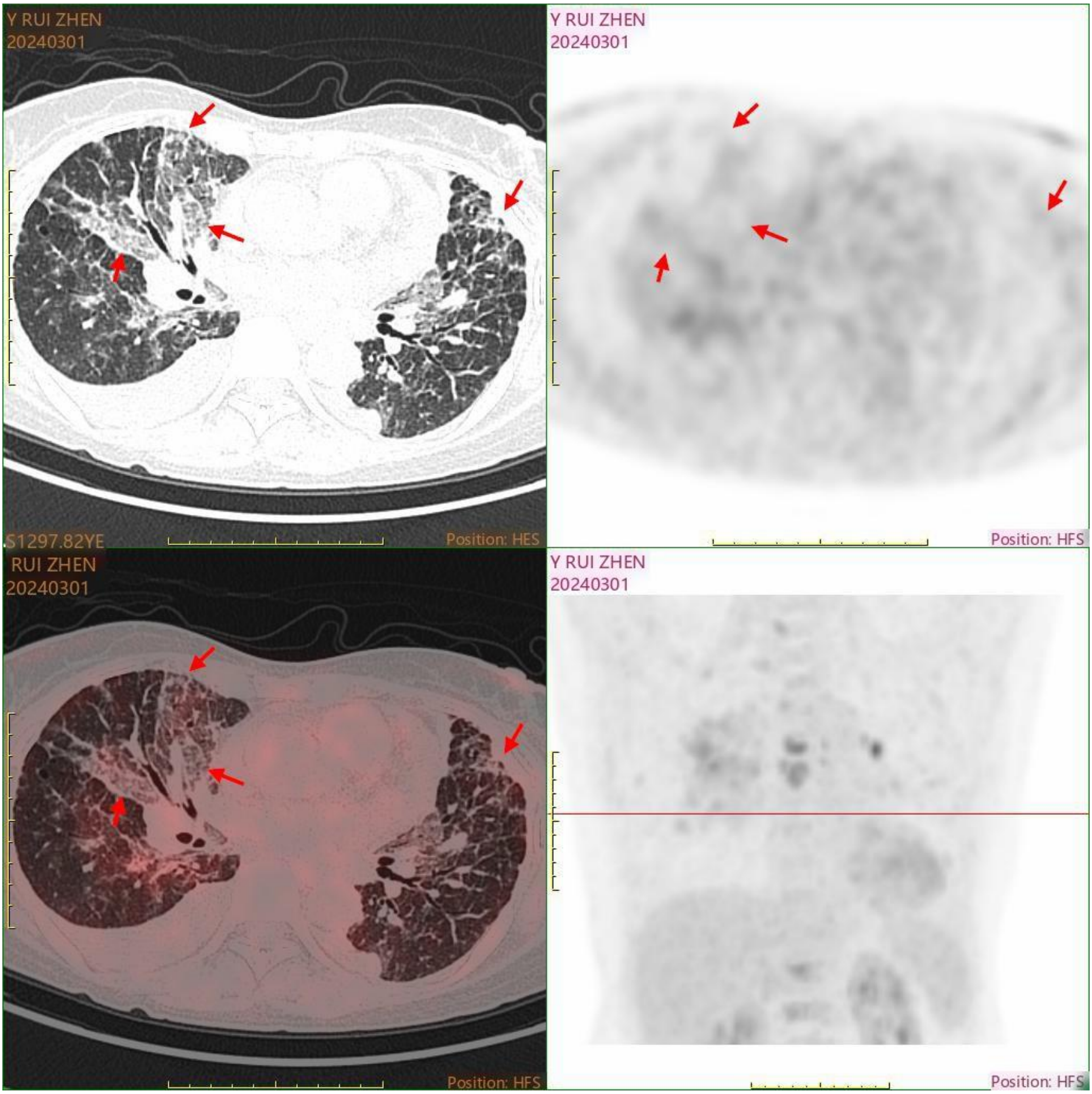

Address:Pavilion 21, No.117 Liuhua Road, Yuexiu Tel: 020-83220688

PET-CT

Name: YEH **Sex:** **Age: 63** **Inspection date: 2024-** **Inspection No.:**

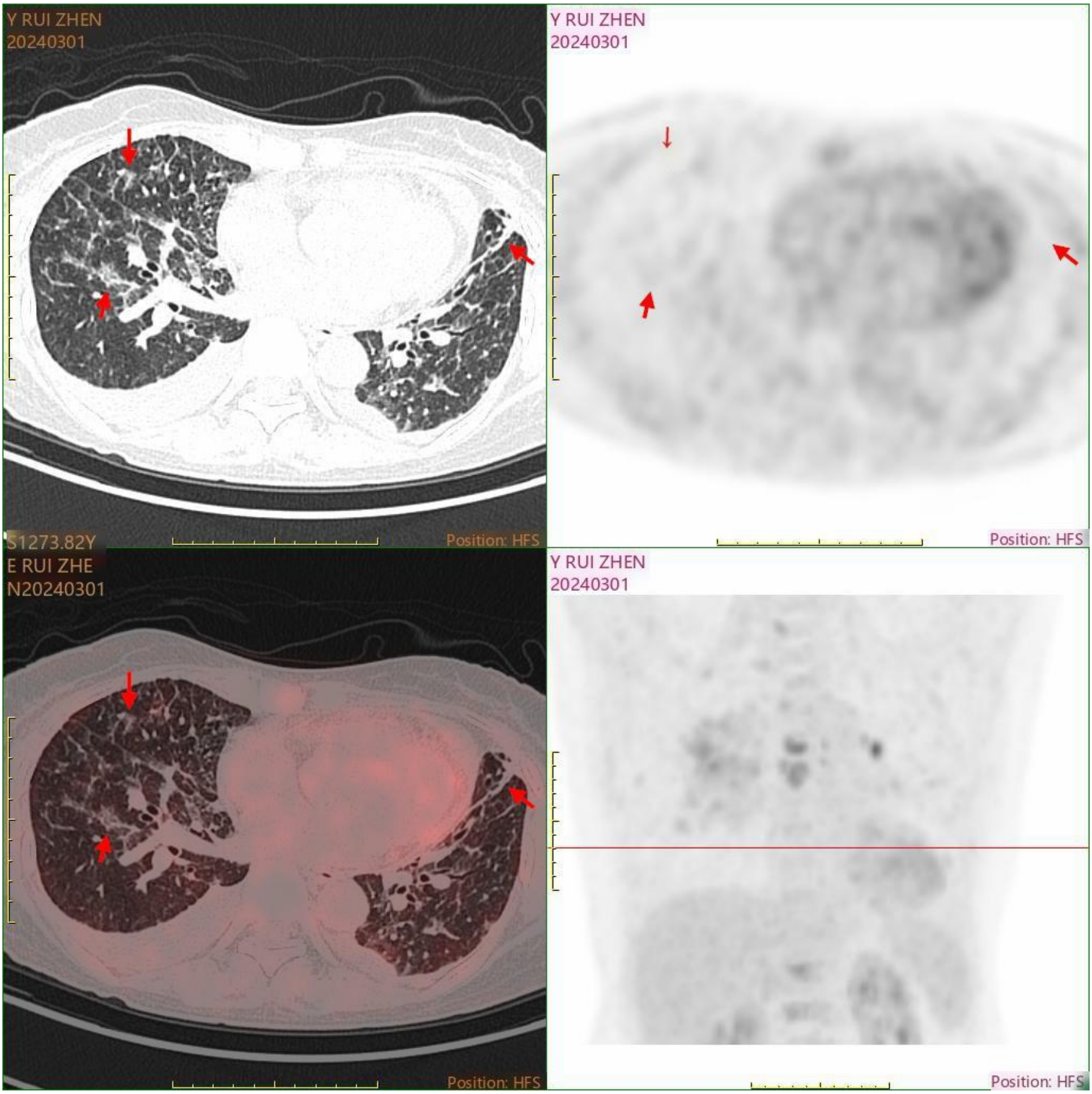

Address:Pavilion 21, No.117 Liuhua Road, Yuexiu Tel: 020-83220688

PET-CT

Name: YEH **Sex:** **Age: 63** **Inspection date: 2024-** **Inspection No.:**

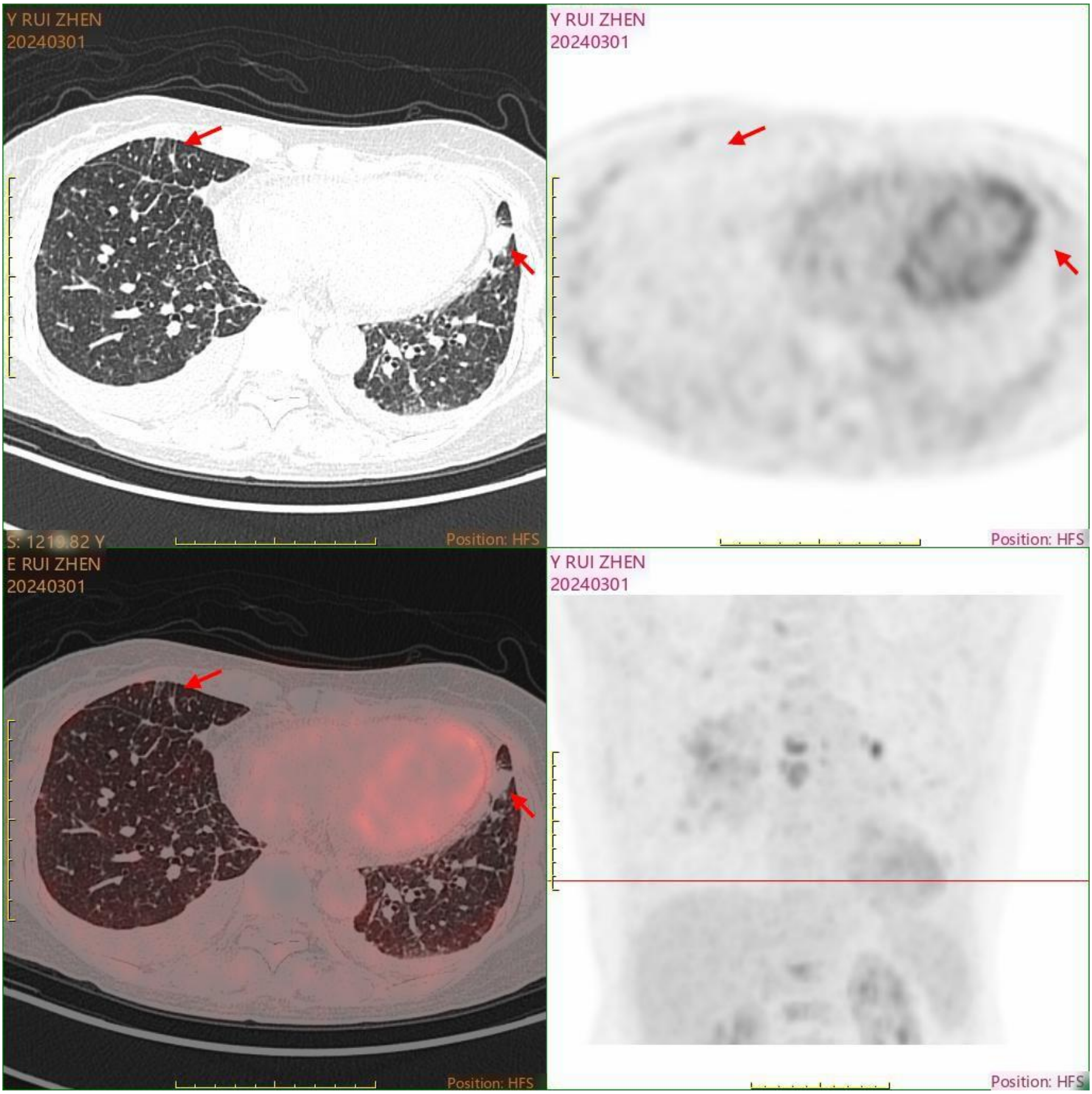

Address:Pavilion 21, No.117 Liuhua Road, Yuexiu
Tel: 020-83220688

PET-CT

Name: YEH **Sex:** **Age: 63** **Inspection date: 2024-** **Inspection No.:**

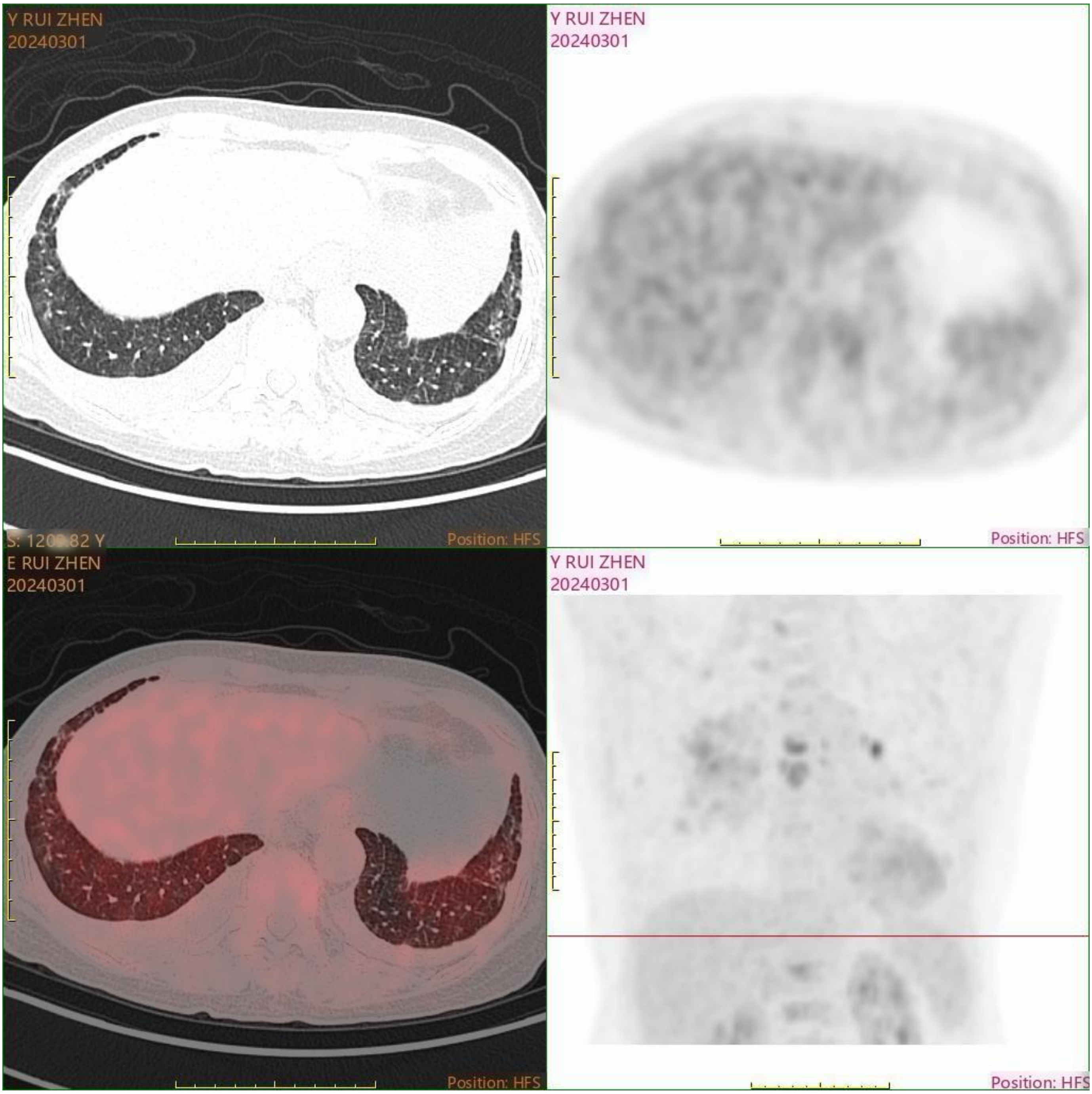

Address:Pavilion 21, No.117 Liuhua Road, Yuexiu Tel: 020-83220688

PET-CT

Name: YEH **Sex:** **Age: 63** **Inspection date: 2024-** **Inspection No.:**

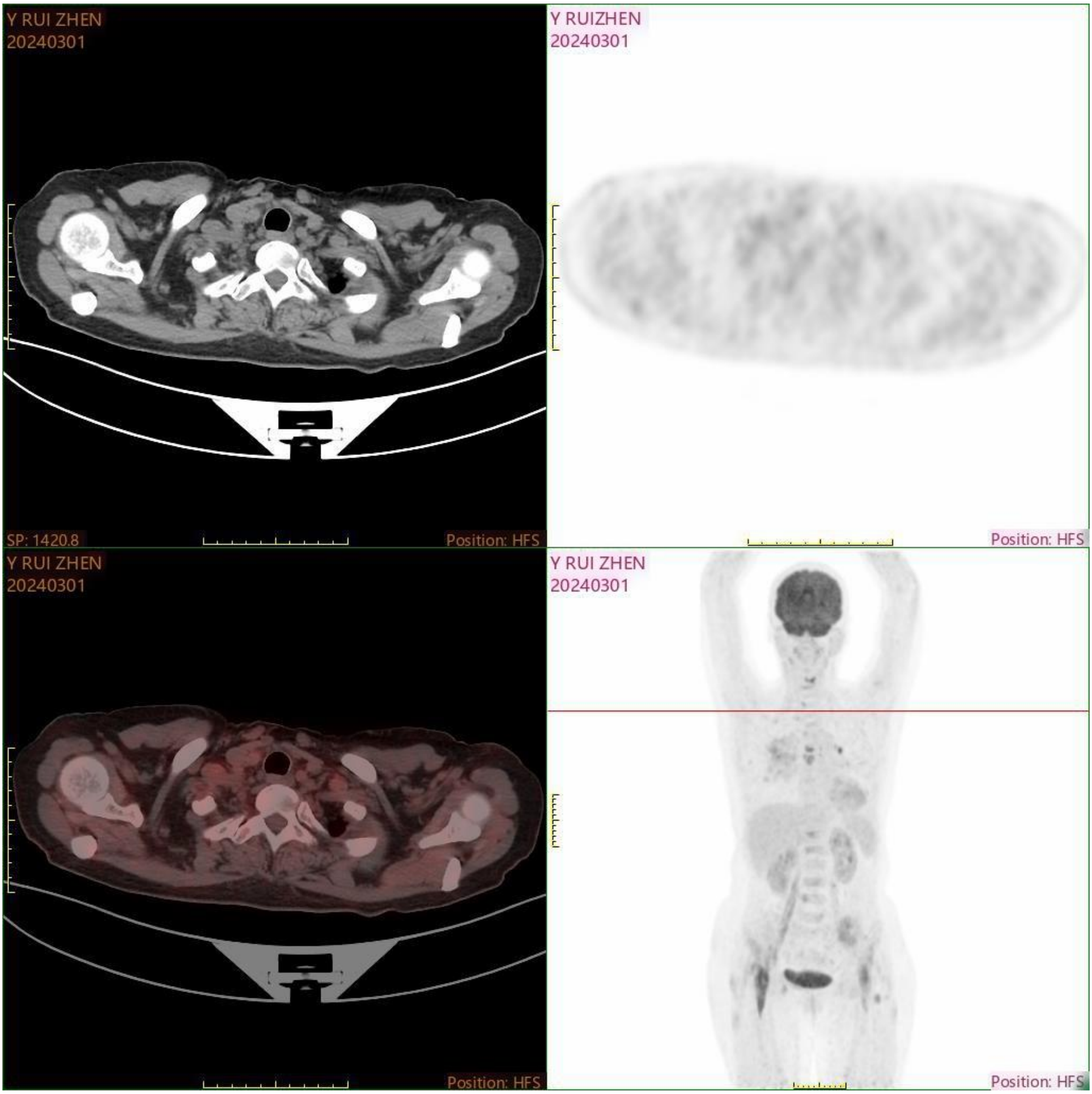

Address:Pavilion 21, No.117 Liuhua Road, Yuexiu Tel: 020-83220688

PET-CT

Name: YEH **Sex:** **Age: 63** **Inspection date: 2024-** **Inspection No.:**

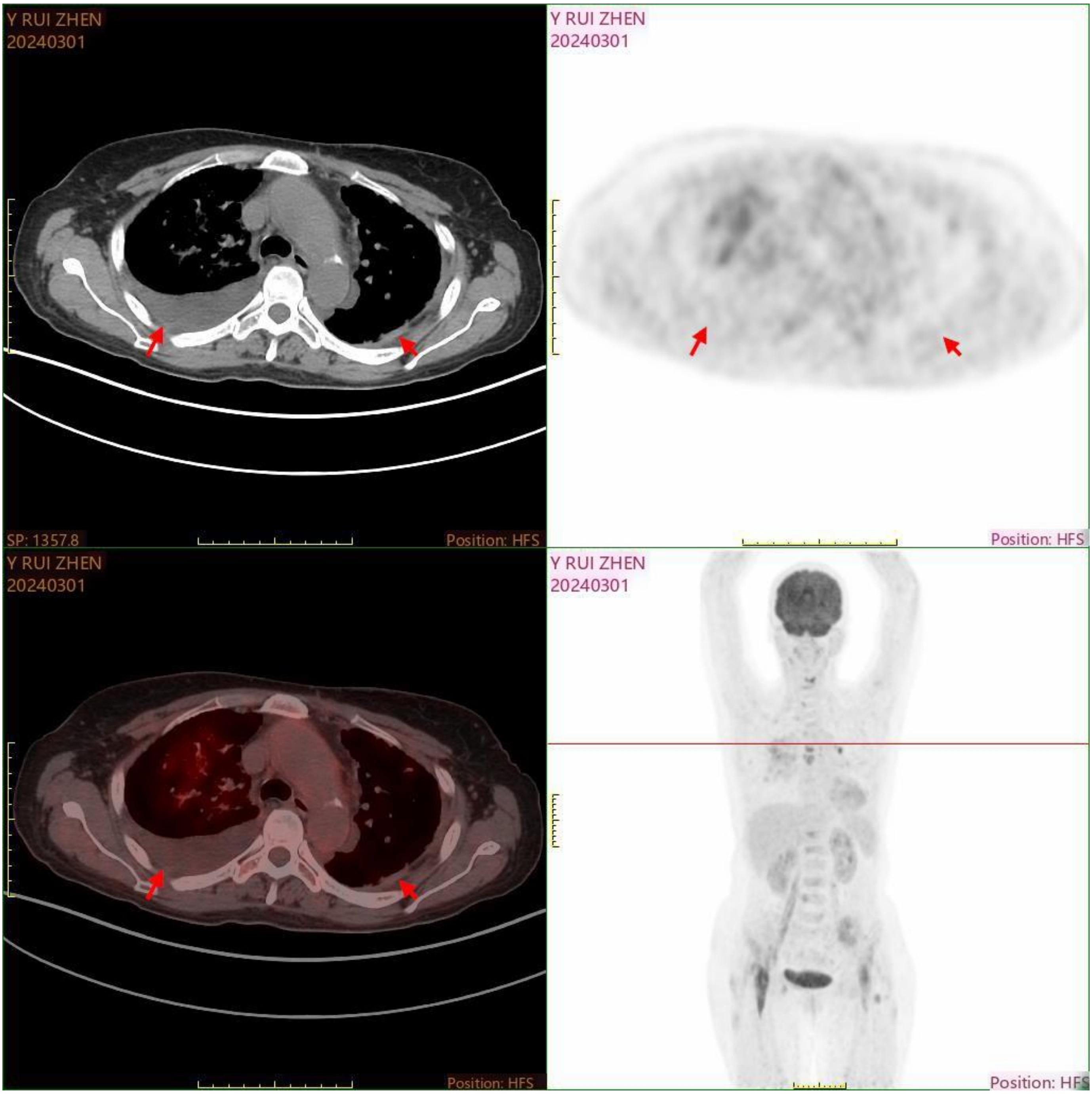

Address:Pavilion 21, No.117 Liuhua Road, Yuexiu Tel: 020-83220688

PET-CT

Name: YEH **Sex:** **Age: 63** **Inspection date: 2024-** **Inspection No.:**

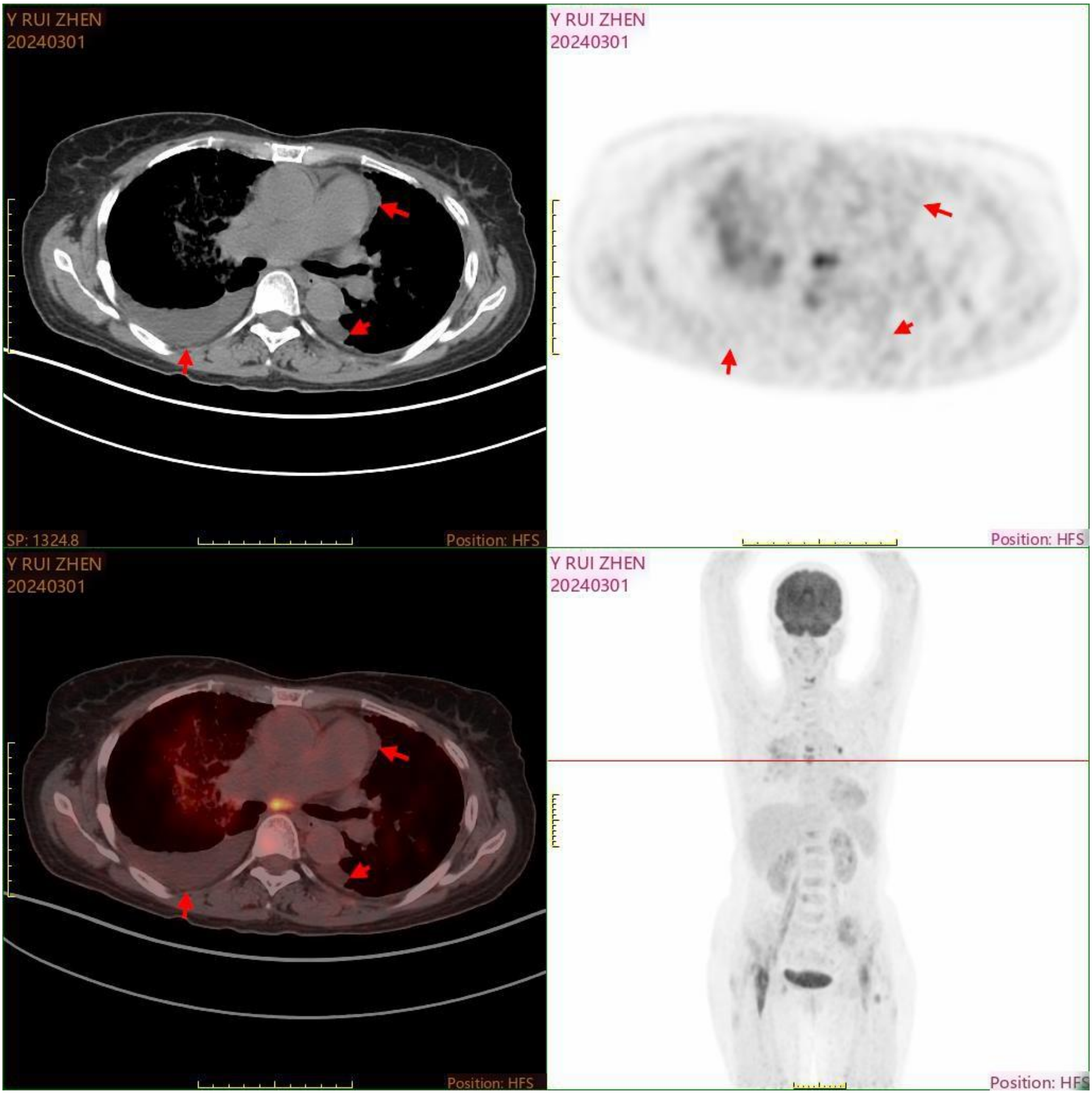

Address:Pavilion 21, No.117 Liuhua Road, Yuexiu Tel: 020-83220688

PET-CT

Name: YEH **Sex:** **Age: 63** **Inspection date: 2024-** **Inspection No.:**

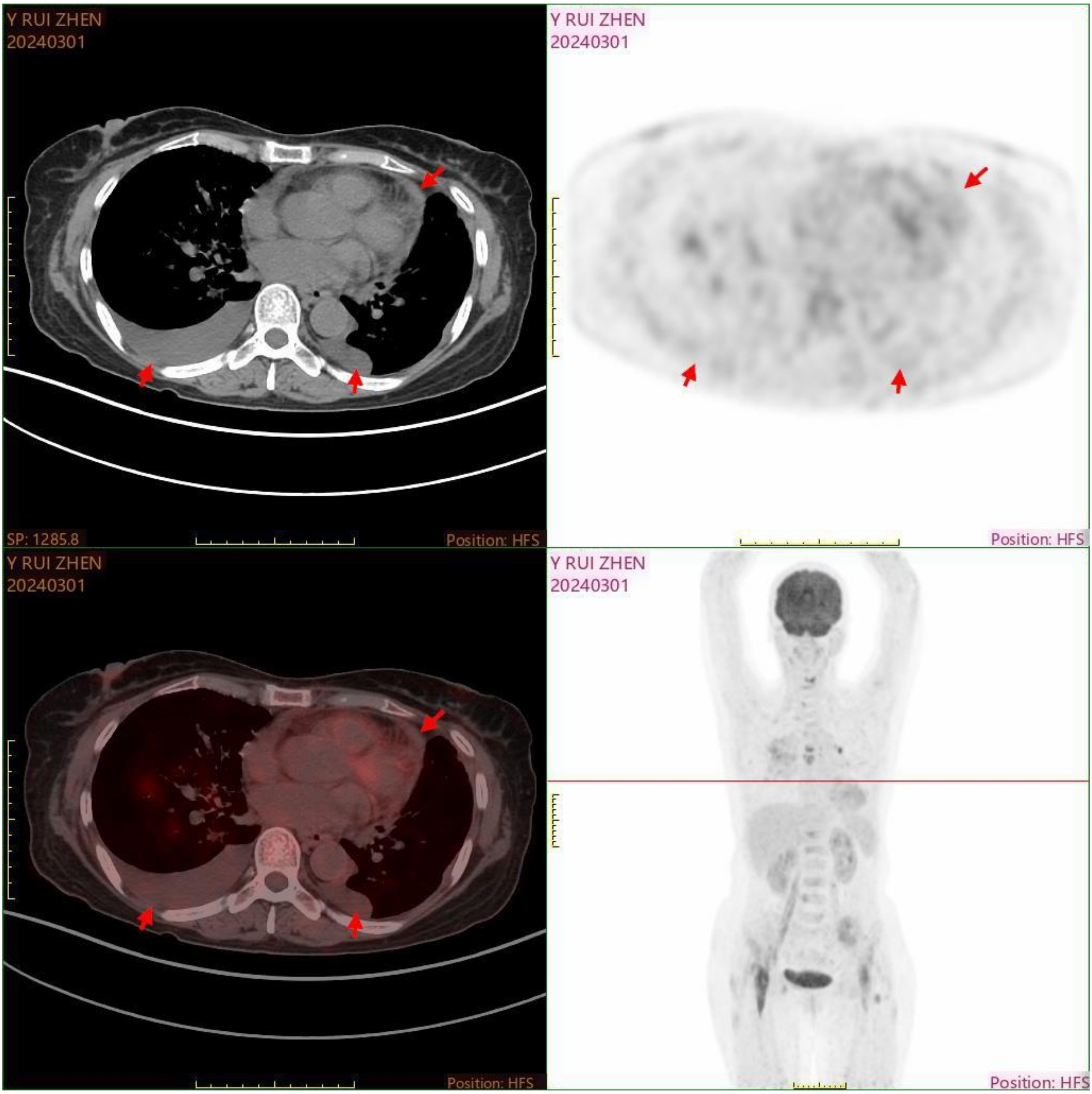

PET-CT

Name: YEH **Sex:** **Age: 63** **Inspection date: 2024-** **Inspection No.:**

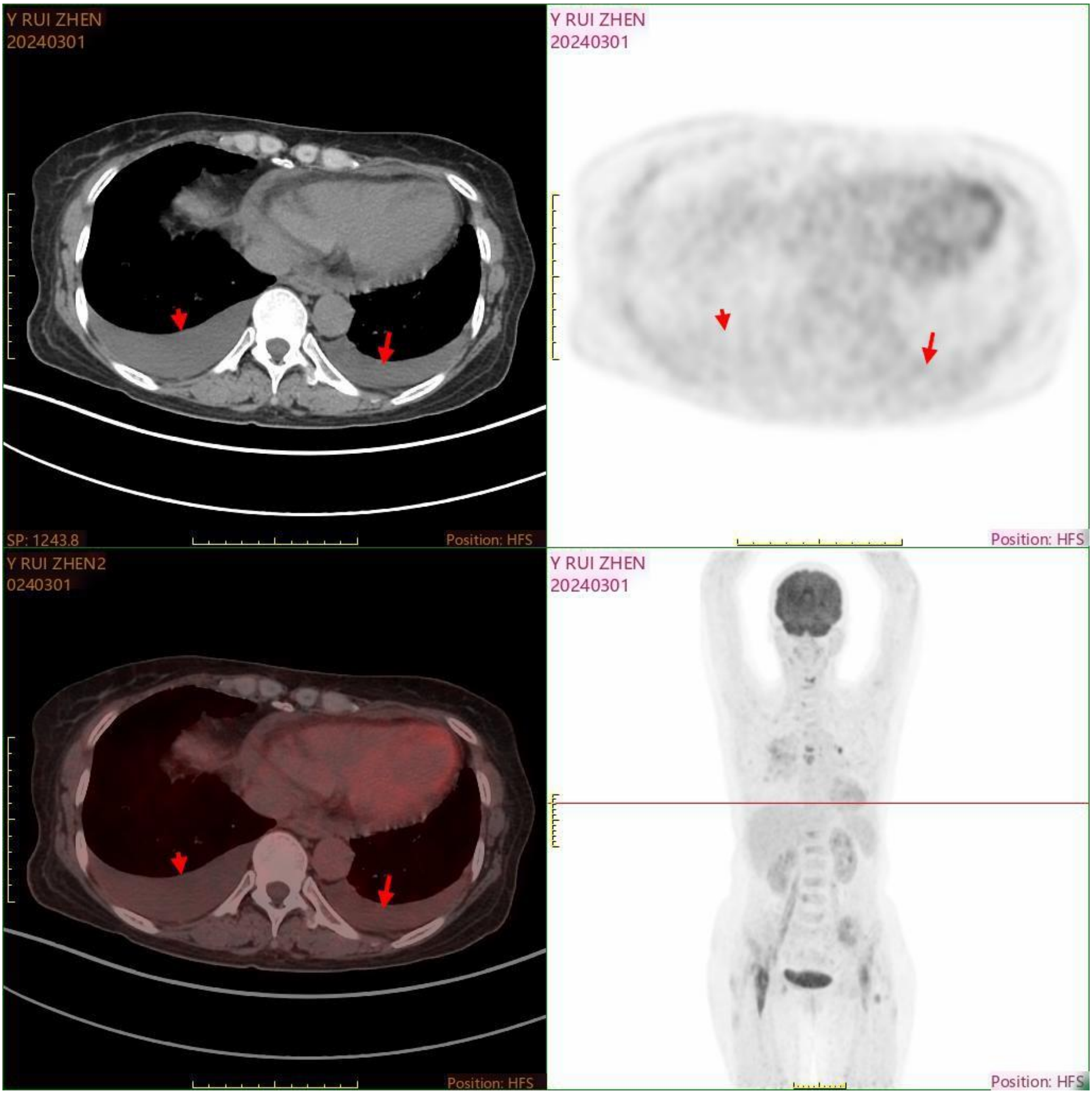

Address:Pavilion 21, No.117 Liuhua Road, Yuexiu Tel: 020-83220688

PET-CT

Name: YEH **Sex:** **Age: 63** **Inspection date: 2024-** **Inspection No.:**

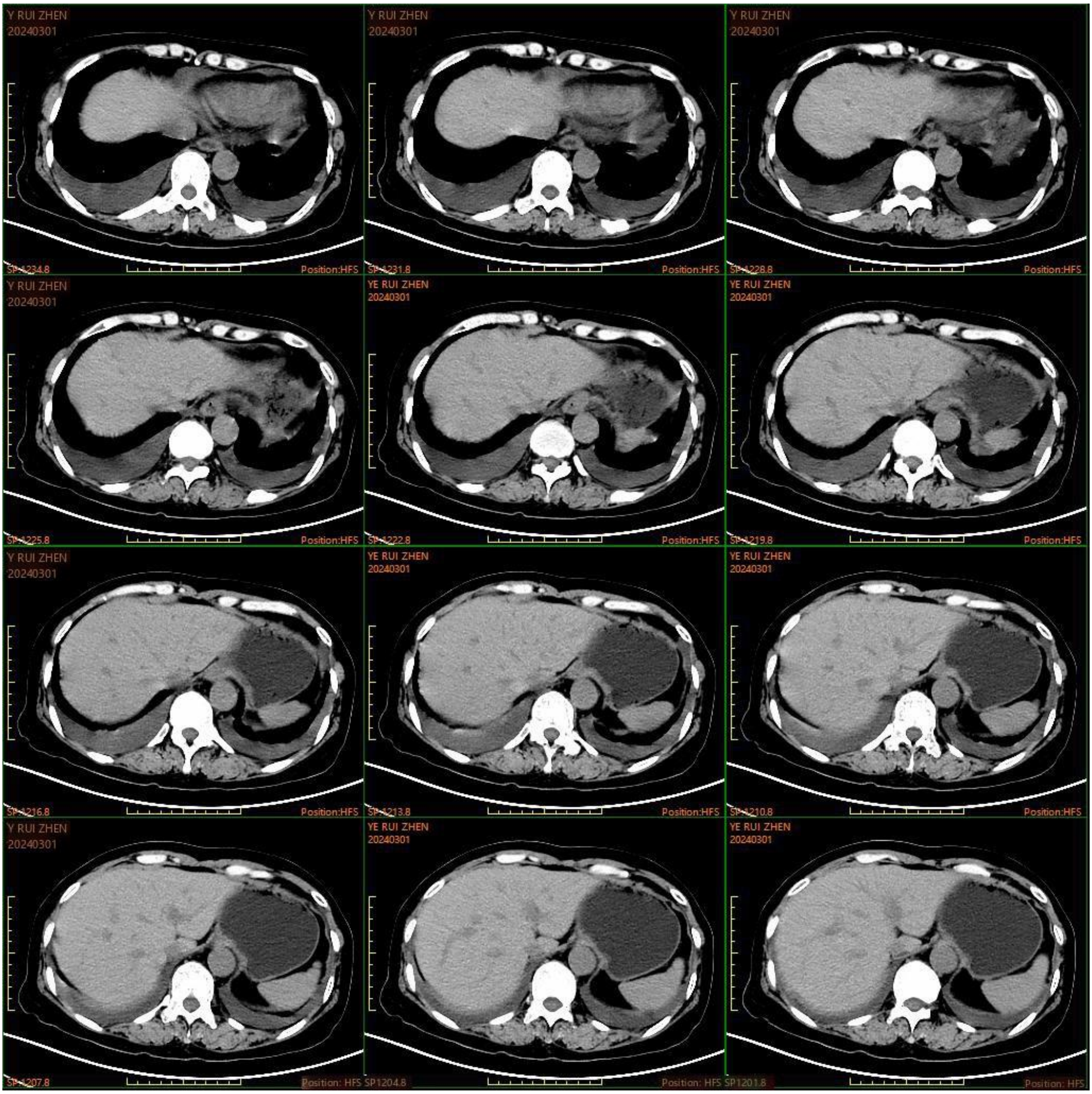

Address:Pavilion 21, No.117 Liuhua Road, Yuexiu
Tel: 020-83220688

PET-CT

Name: YEH **Sex:** **Age: 63** **Inspection date: 2024-** **Inspection No.:**

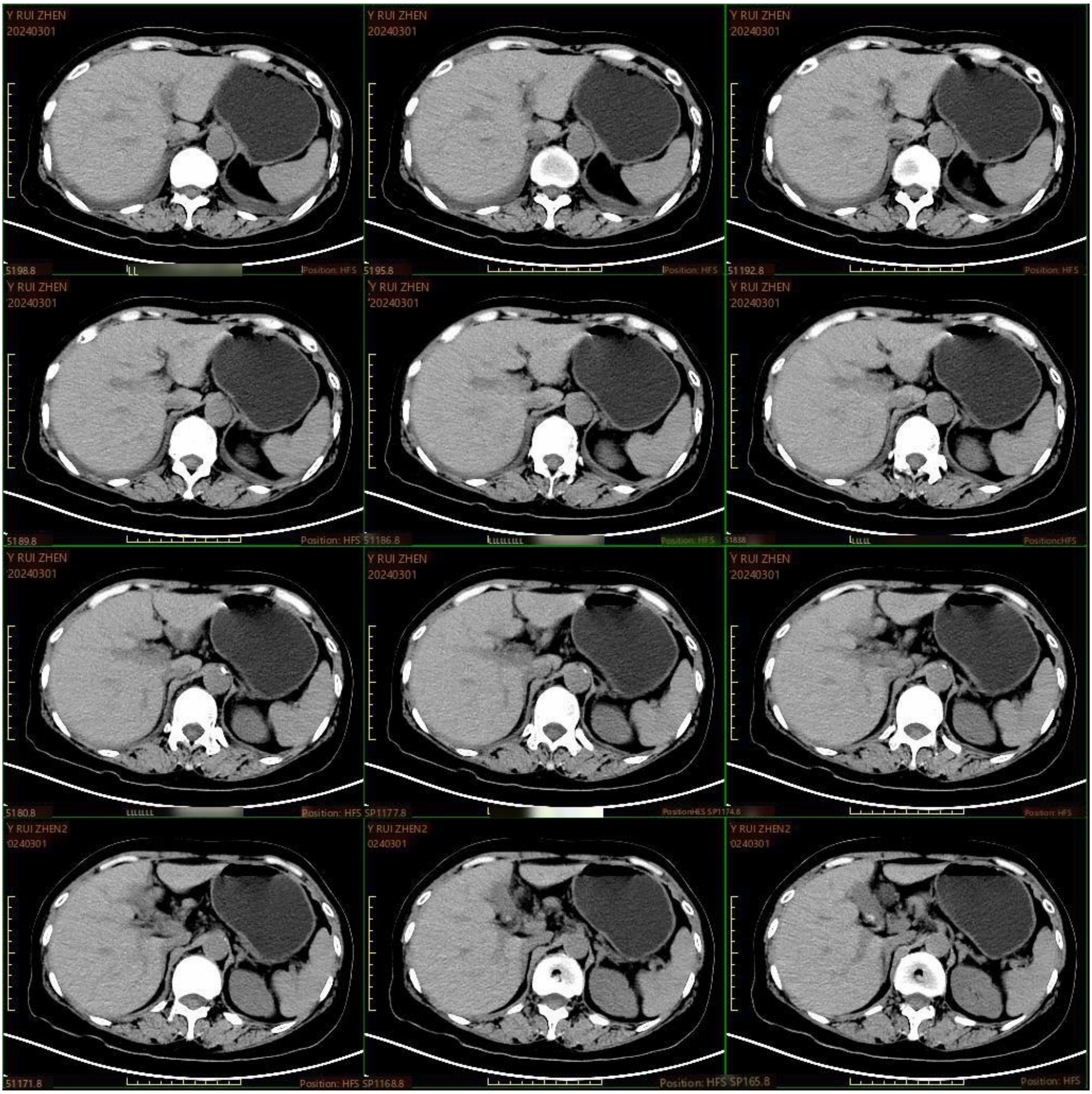

Address:Pavilion 21, No.117 Liuhua Road, Yuexiu
Tel: 020-83220688

Guangzhou Huyun Medical Imaging Diagnosis Center

Guangzhou Huyun Medical Imaging Diagnostic Center

PET-CT

Name: YEH **Sex:** **Age: 63** **Inspection date: 2024-** **Inspection No.:**

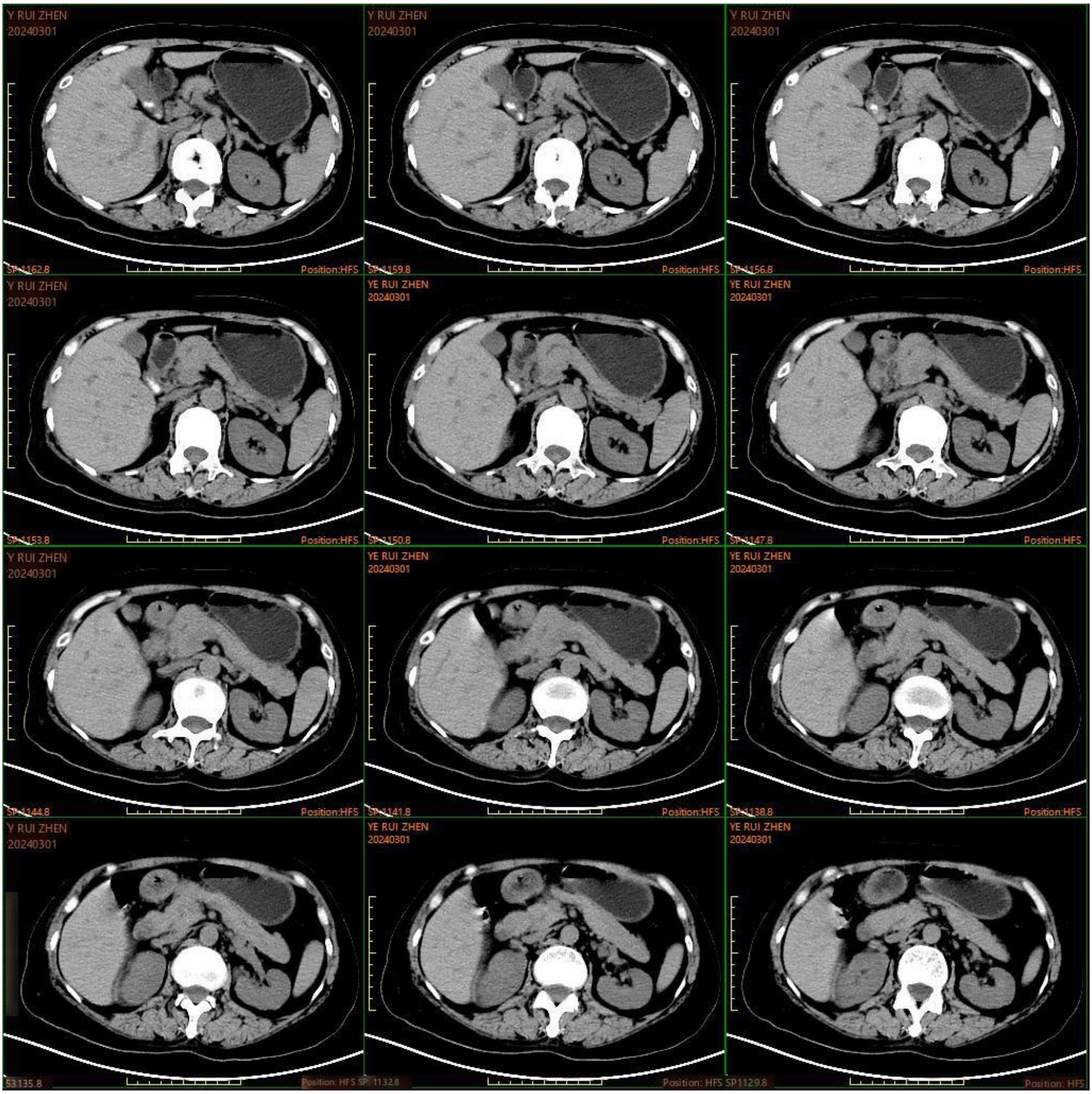

Address:Pavilion 21, No.117 Liuhua Road, Yuexiu Tel: 020-83220688

PET-CT

Name: YEH **Sex:** **Age: 63** **Inspection date: 2024-** **Inspection No.:**

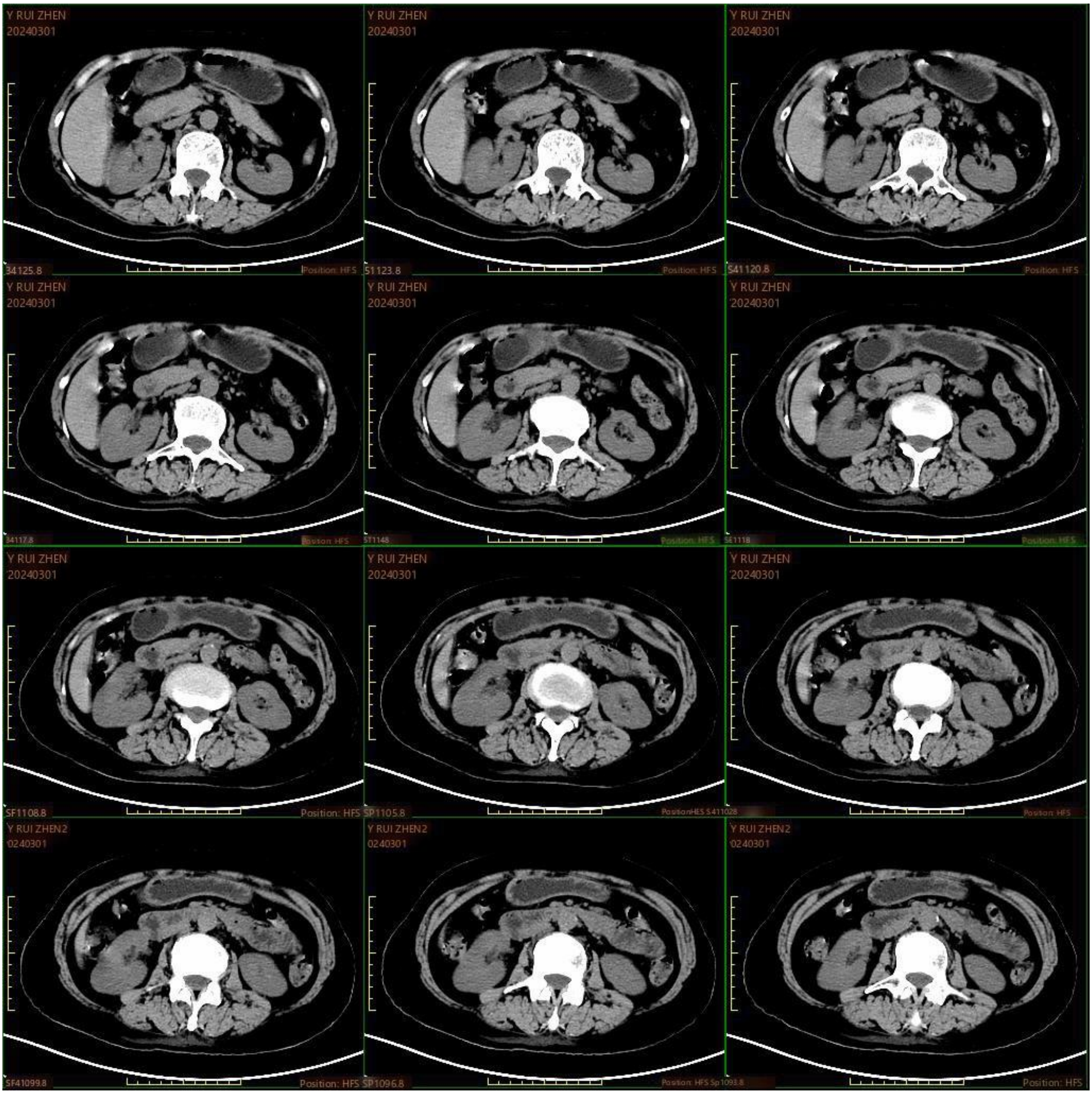

Address:Pavilion 21, No.117 Liuhua Road, Yuexiu Tel: 020-83220688

PET-CT

Name: YEH **Sex:** **Age: 63** **Inspection date: 2024-** **Inspection No.:**

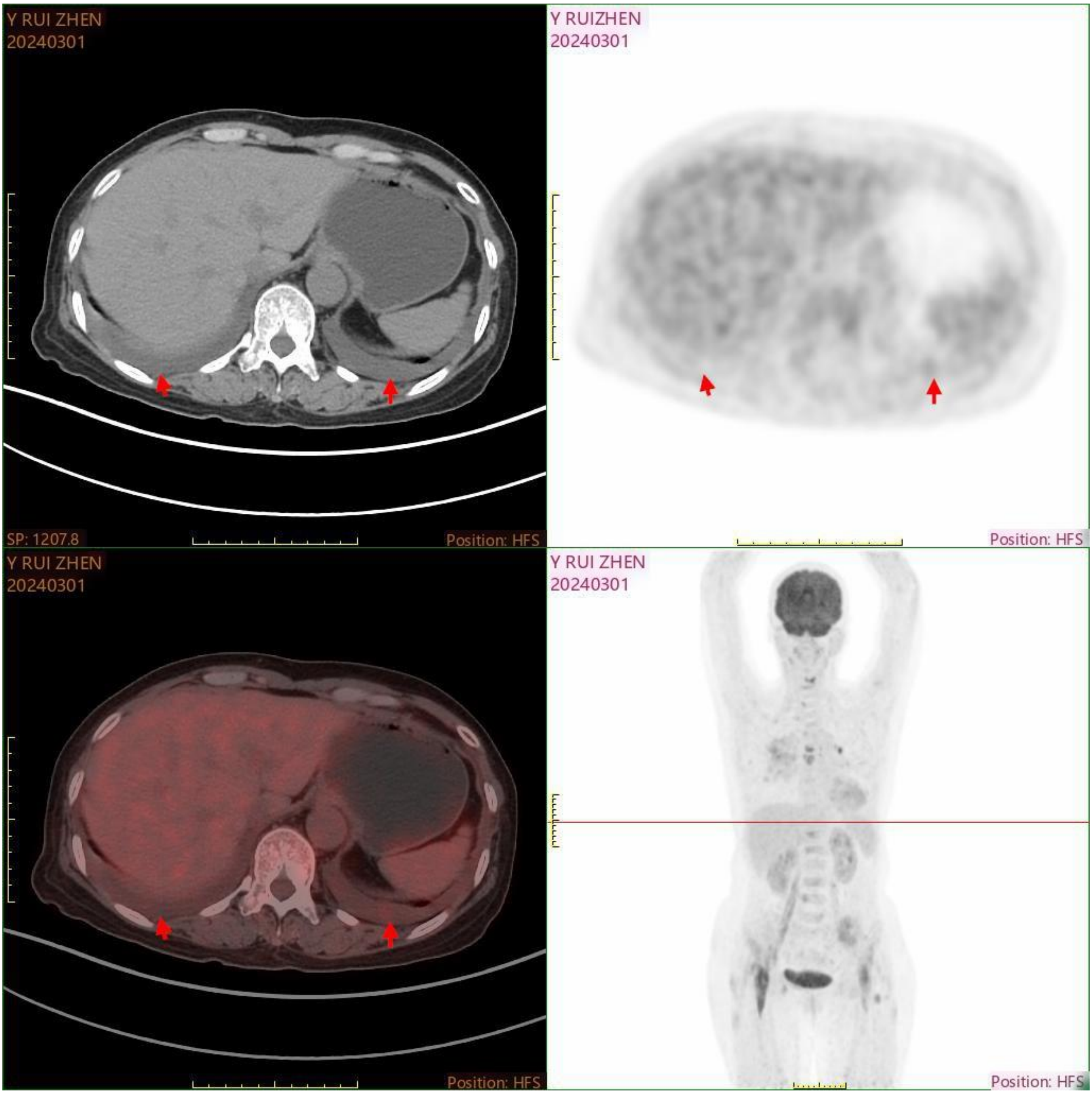

PET-CT

Name: YEH **Sex:** **Age: 63** **Inspection date: 2024-** **Inspection No.:**

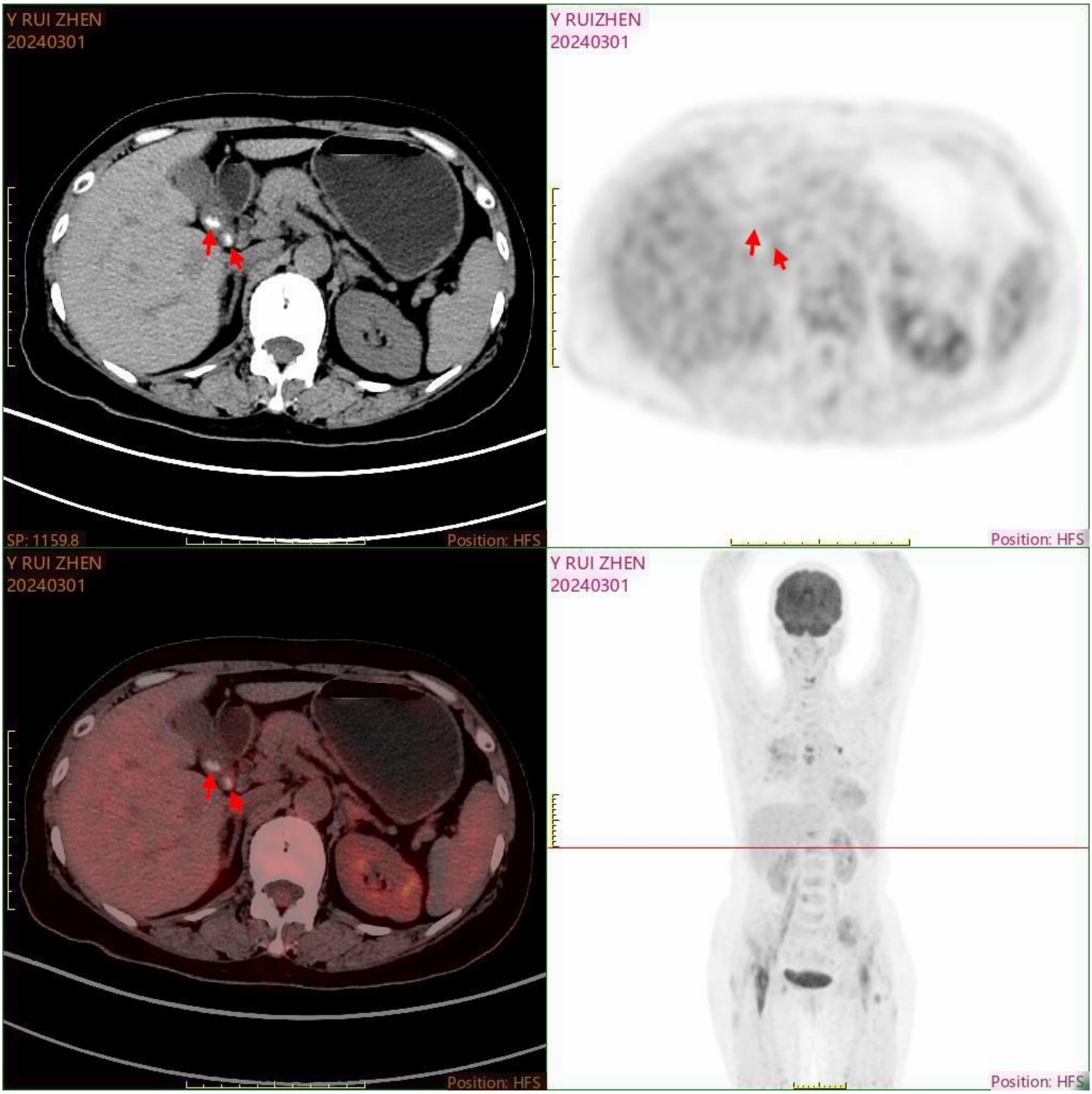

Address:Pavilion 21, No.117 Liuhua Road, Yuexiu Tel: 020-83220688

PET-CT

Name: YEH **Sex:** **Age: 63** **Inspection date: 2024-** **Inspection No.:**

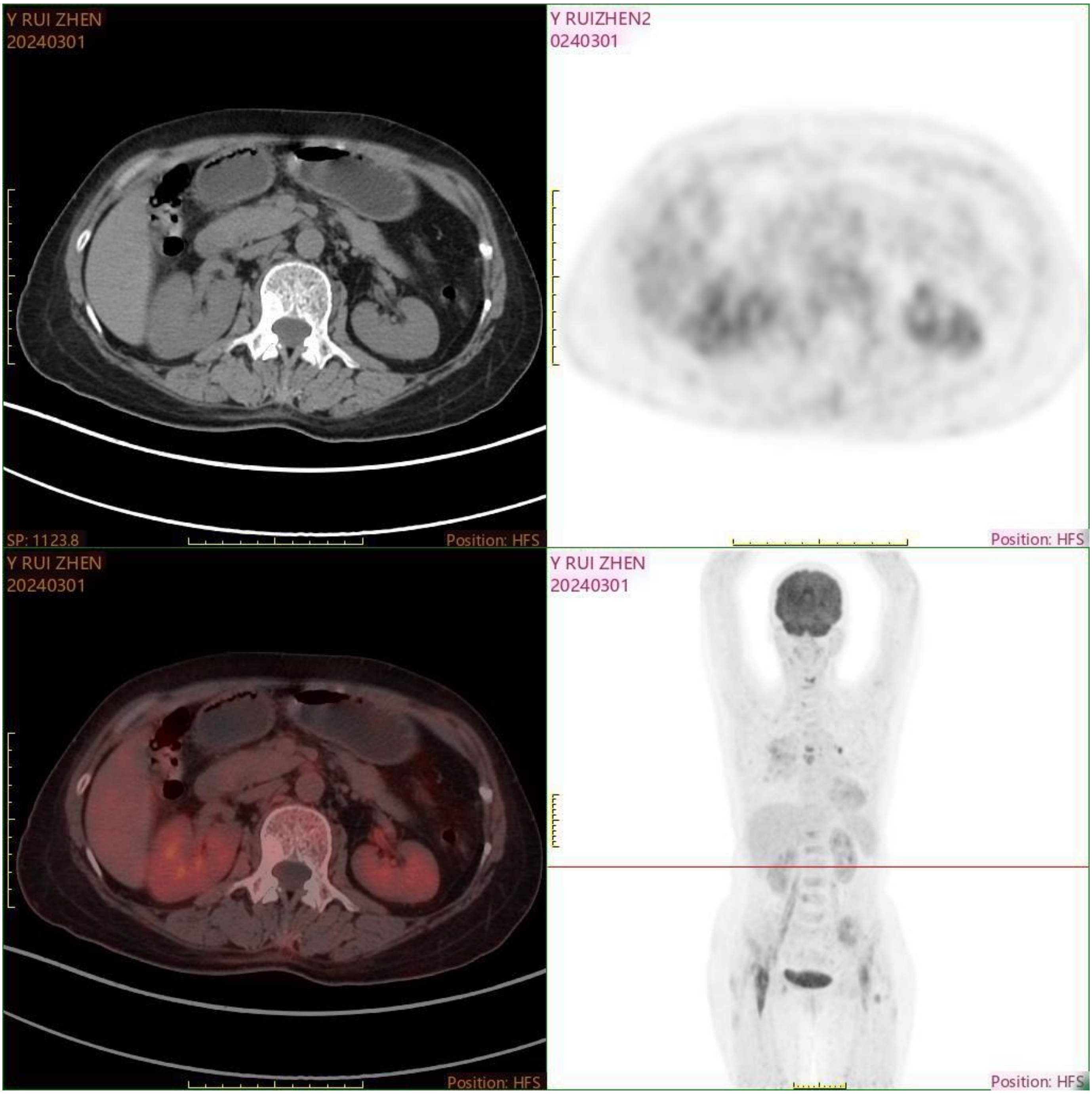

Address:Pavilion 21, No.117 Liuhua Road, Yuexiu Tel: 020-83220688

PET-CT

Name: YEH **Sex:** **Age: 63** **Inspection date: 2024-** **Inspection No.:**

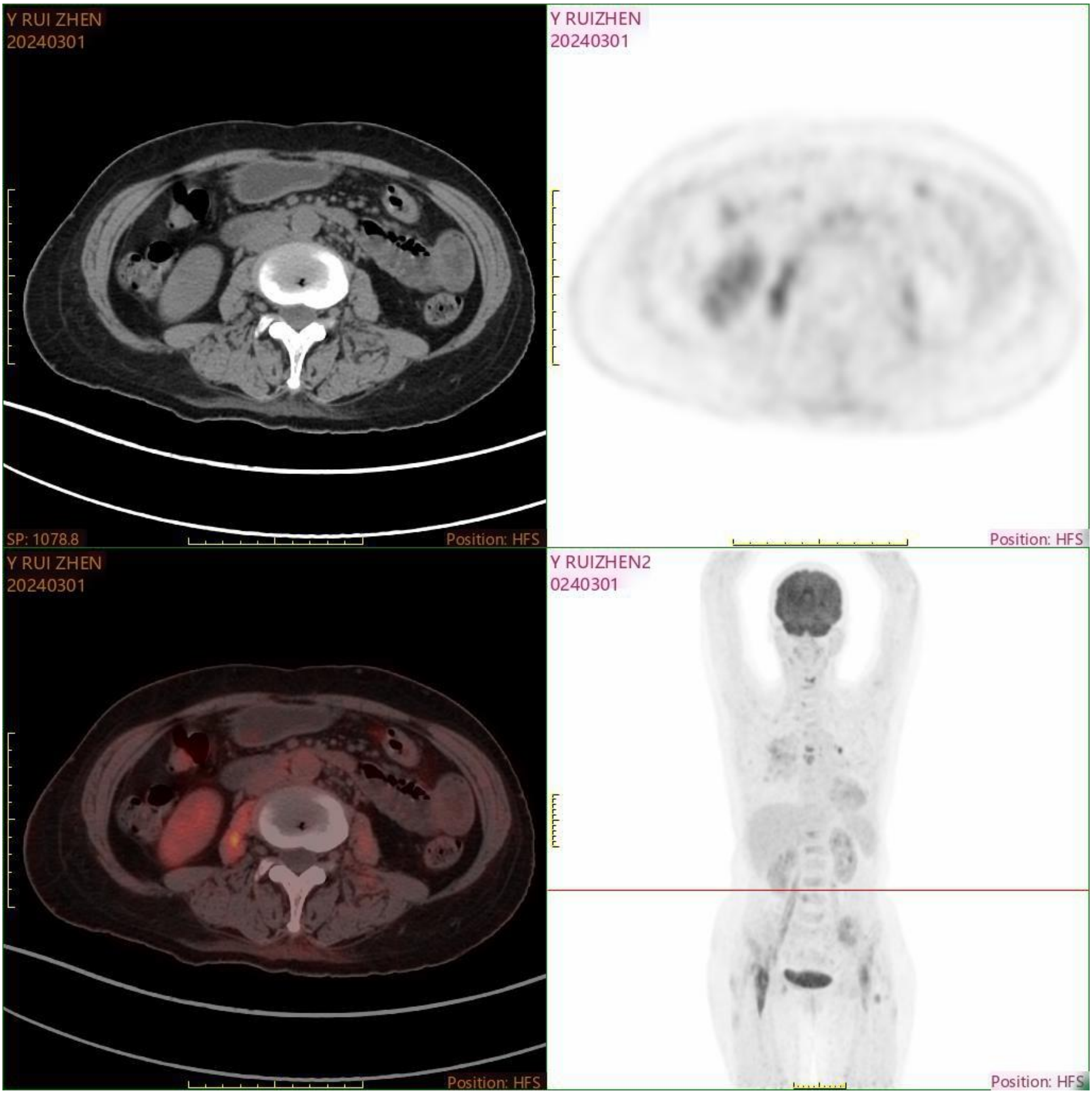

Address:Pavilion 21, No.117 Liuhua Road, Yuexiu Tel: 020-83220688

PET-CT

Name: YEH **Sex:** **Age: 63** **Inspection date: 2024-** **Inspection No.:**

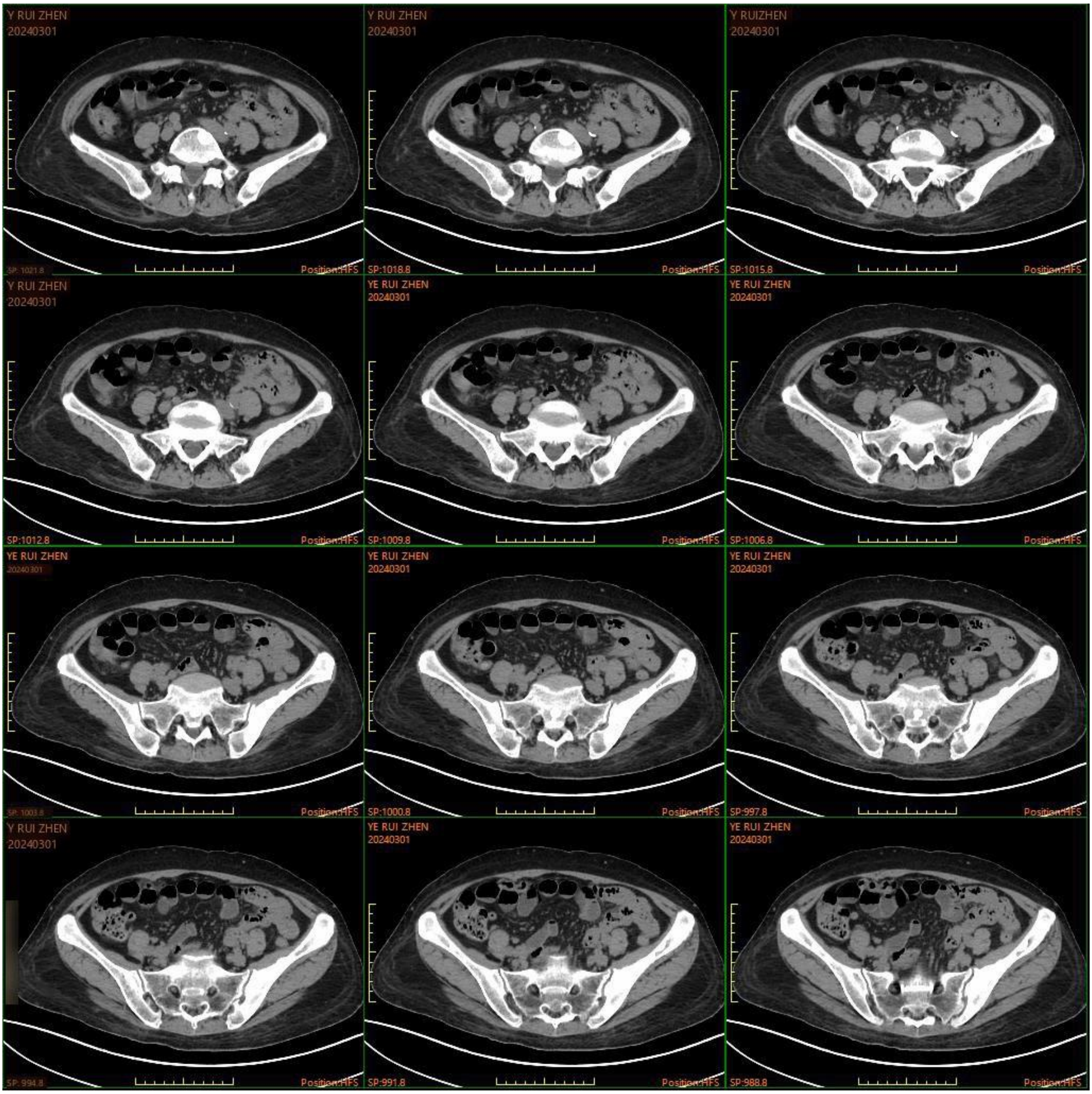

Address:Pavilion 21, No.117 Liuhua Road, Yuexiu Tel: 020-83220688

PET-CT

Name: YEH **Sex:** **Age: 63** **Inspection date: 2024-** **Inspection No.:**

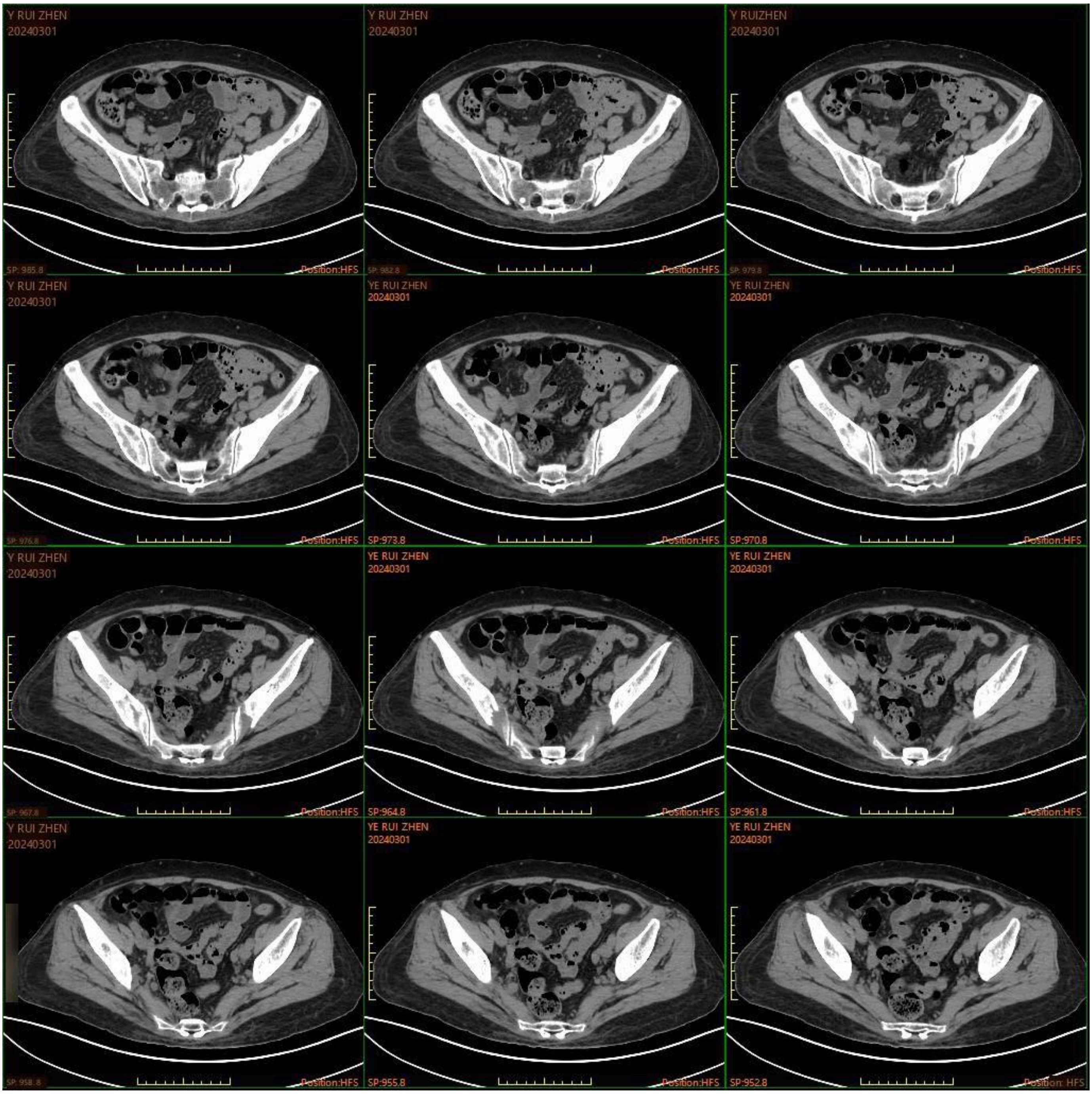

Address:Pavilion 21, No.117 Liuhua Road, Yuexiu Tel: 020-83220688

PET-CT

Name: YEH **Sex:** **Age: 63** **Inspection date: 2024-** **Inspection No.:**

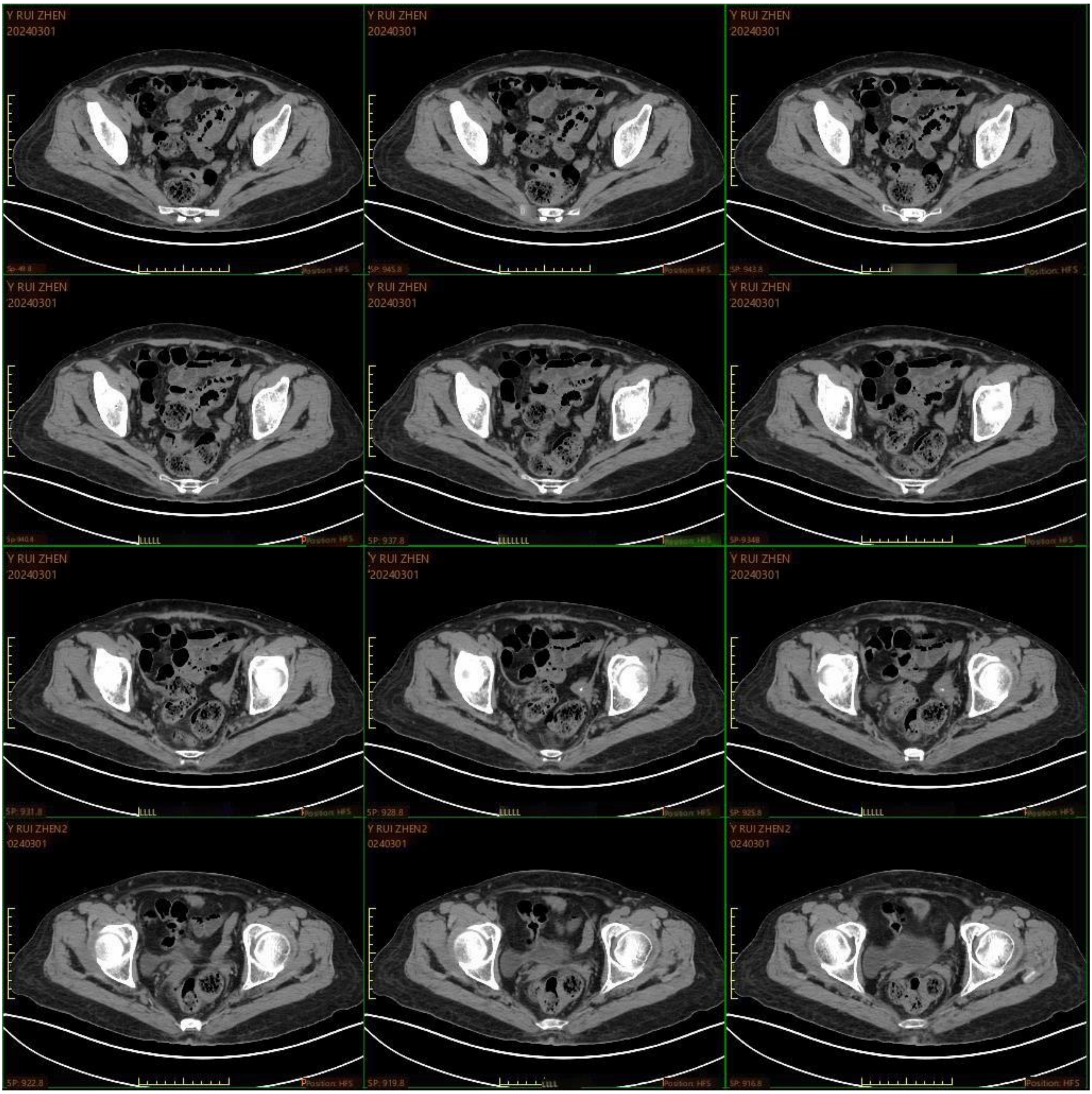

Address:Pavilion 21, No.117 Liuhua Road, Yuexiu Tel: 020-83220688

PET-CT

Name: YEH **Sex:** **Age: 63** **Inspection date: 2024-** **Inspection No.:**

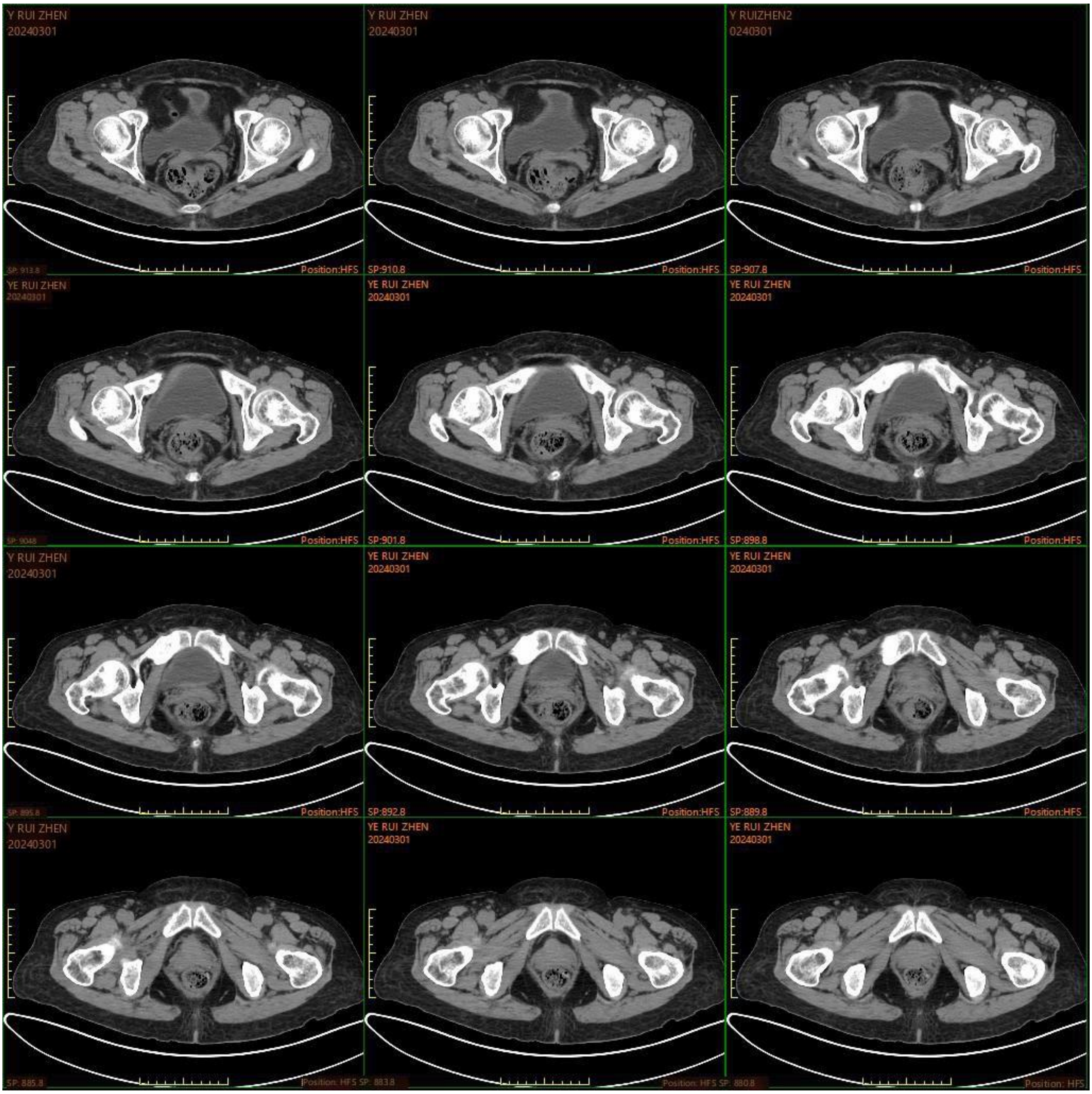

Address:Pavilion 21, No.117 Liuhua Road, Yuexiu Tel: 020-83220688

PET-CT

Name: YEH **Sex:** **Age: 63** **Inspection date: 2024-** **Inspection No.:**

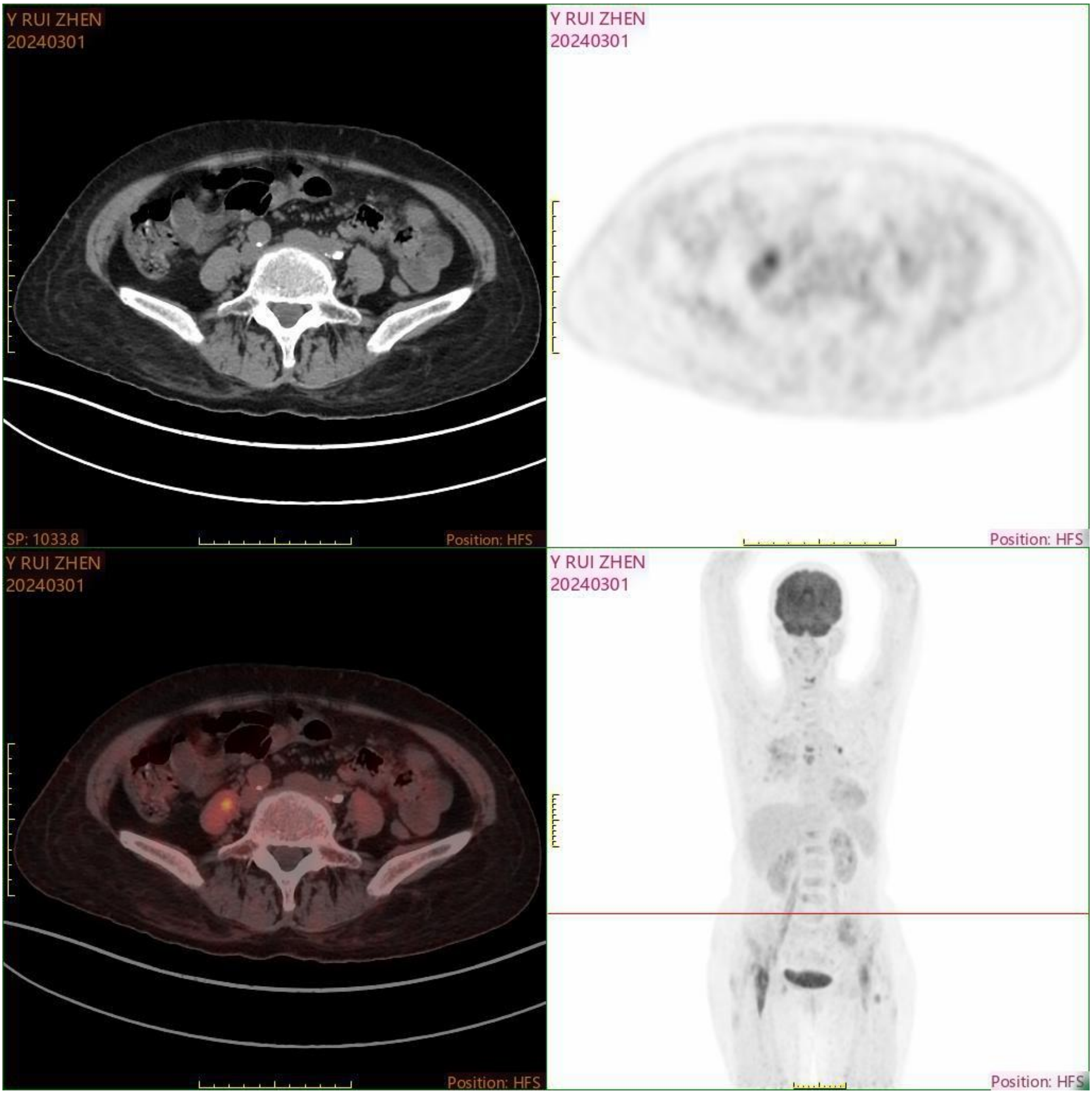

Address:Pavilion 21, No.117 Liuhua Road, Yuexiu
Tel: 020-83220688

PET-CT

Name: YEH **Sex:** **Age: 63** **Inspection date: 2024-** **Inspection No.:**

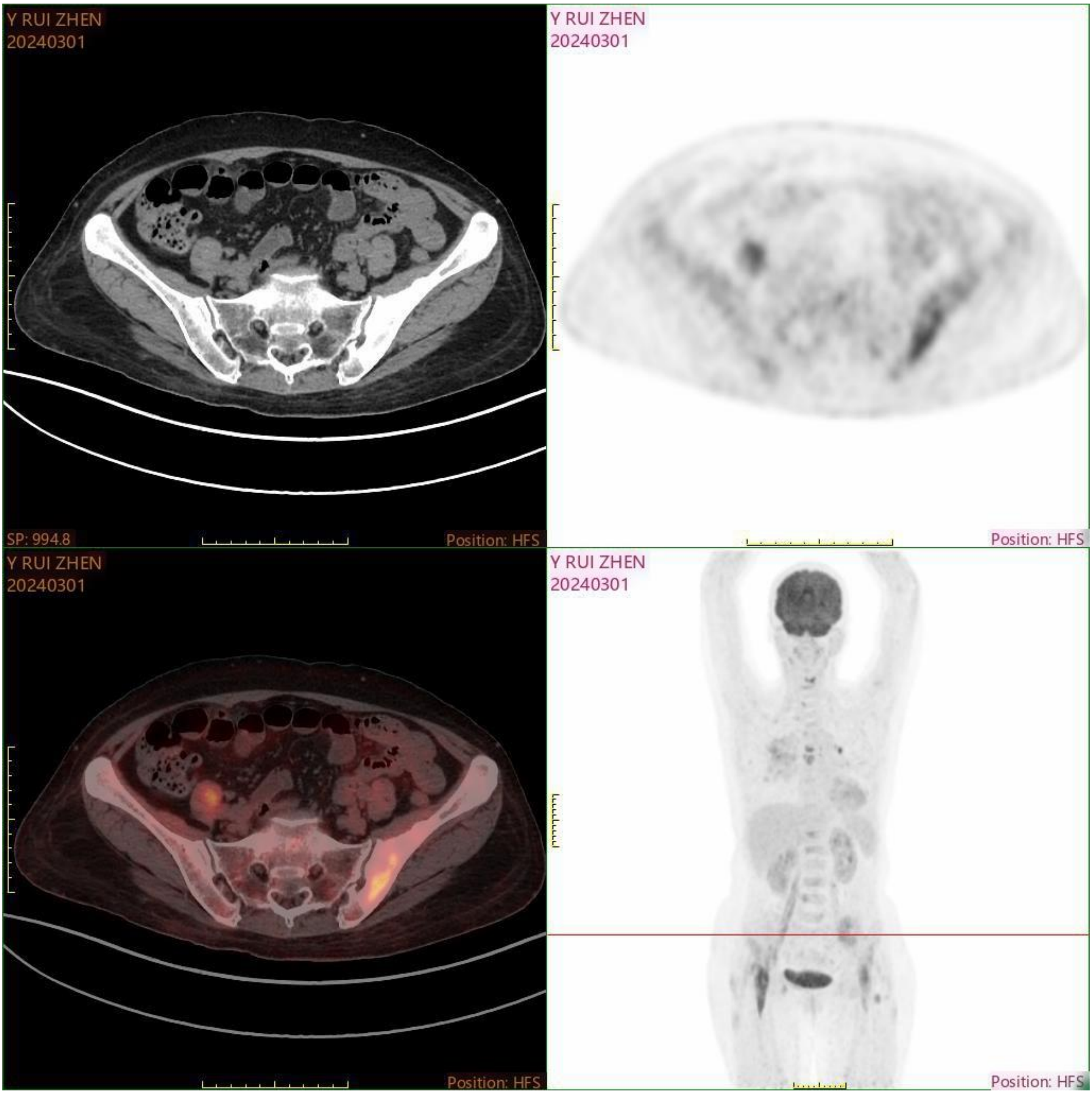

Address:Pavilion 21, No.117 Liuhua Road, Yuexiu
Tel: 020-83220688

PET-CT

Name: YEH **Sex:** **Age: 63** **Inspection date: 2024-** **Inspection No.:**

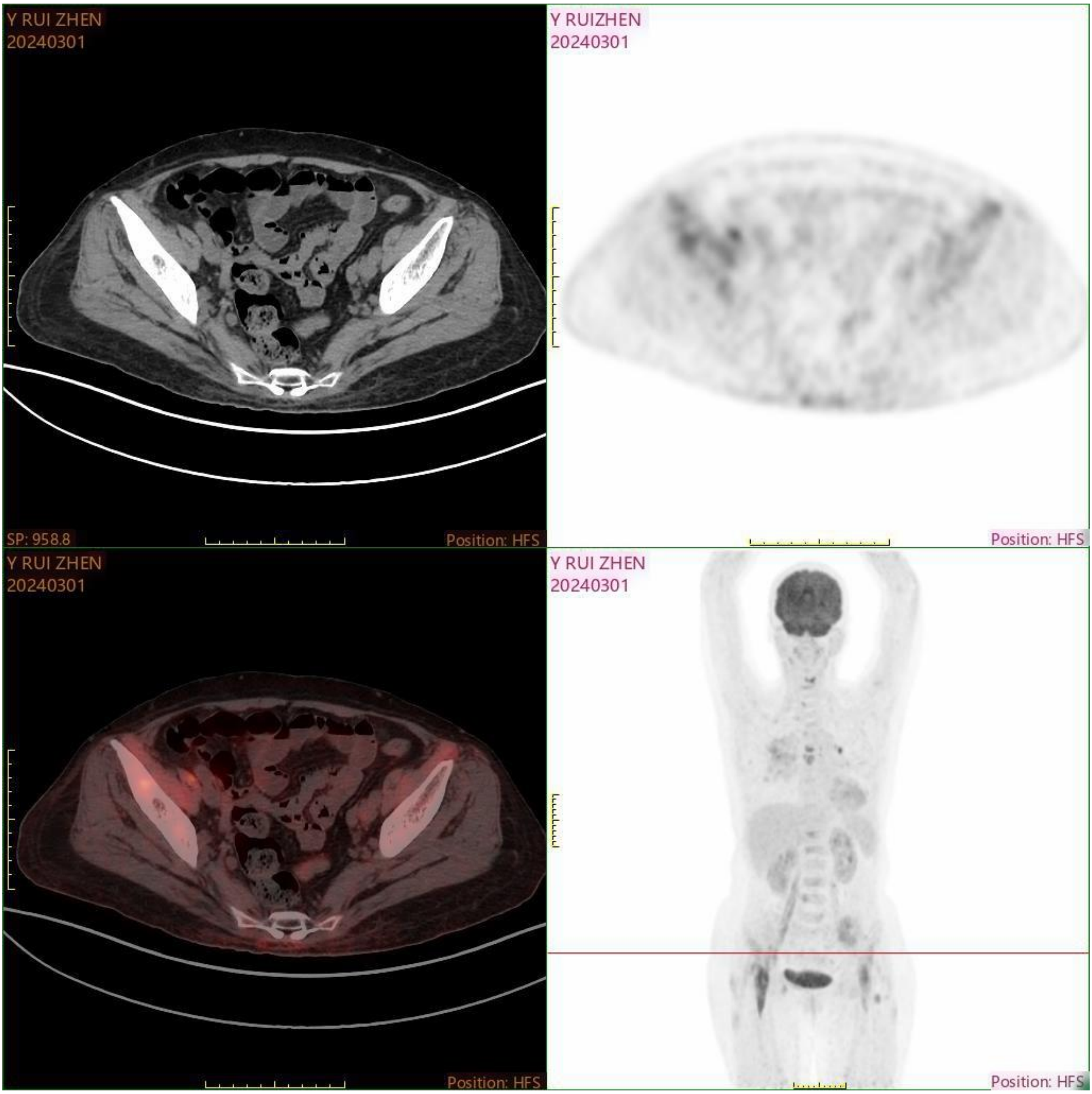

Address:Pavilion 21, No.117 Liuhua Road, Yuexiu
Tel: 020-83220688

PET-CT

Name: YEH **Sex:** **Age: 63** **Inspection date: 2024-** **Inspection No.:**

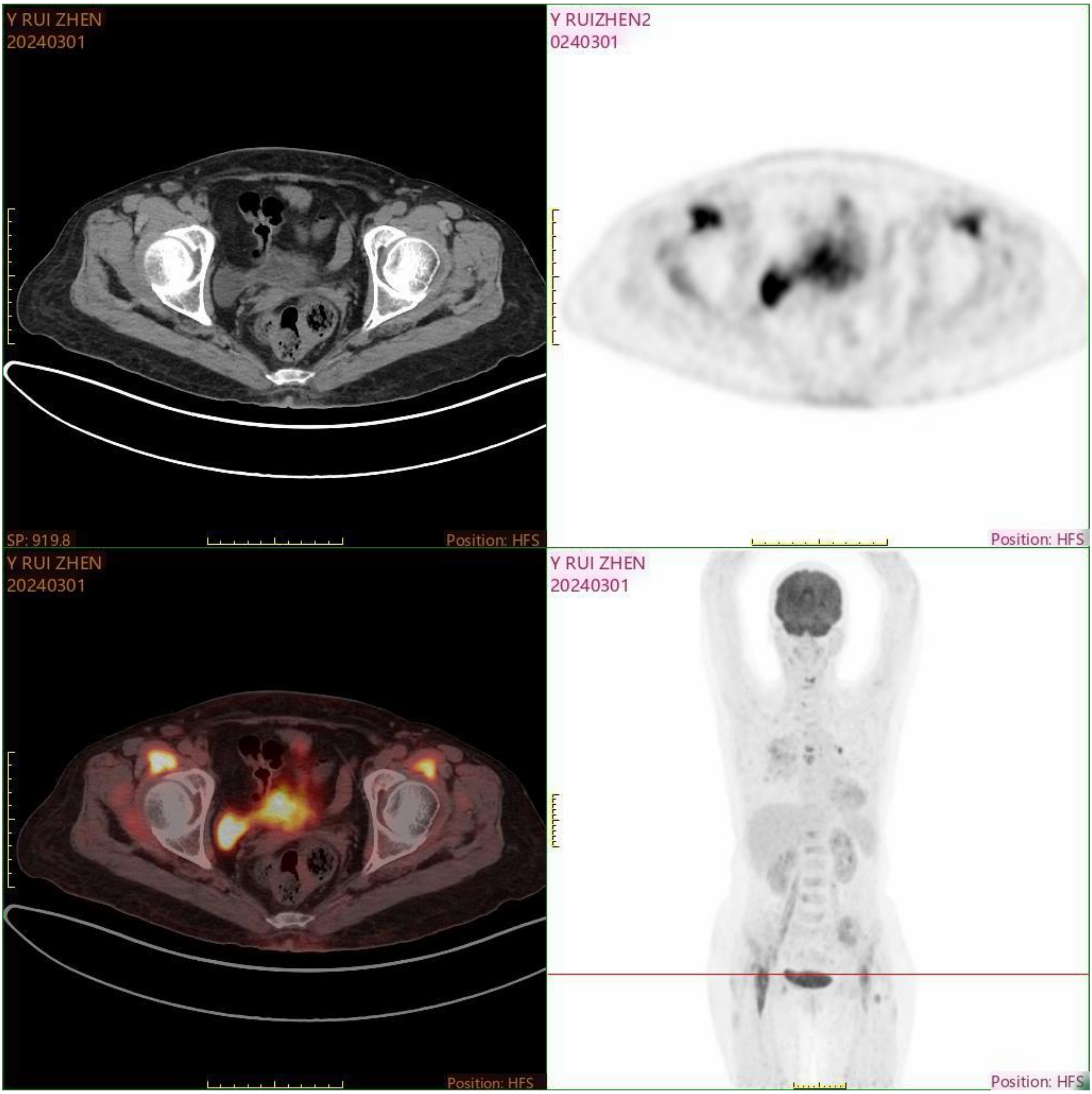

Address:Pavilion 21, No.117 Liuhua Road, Yuexiu Tel: 020-83220688

PET-CT

Name: YEH **Sex:** **Age: 63** **Inspection date: 2024-** **Inspection No.:**

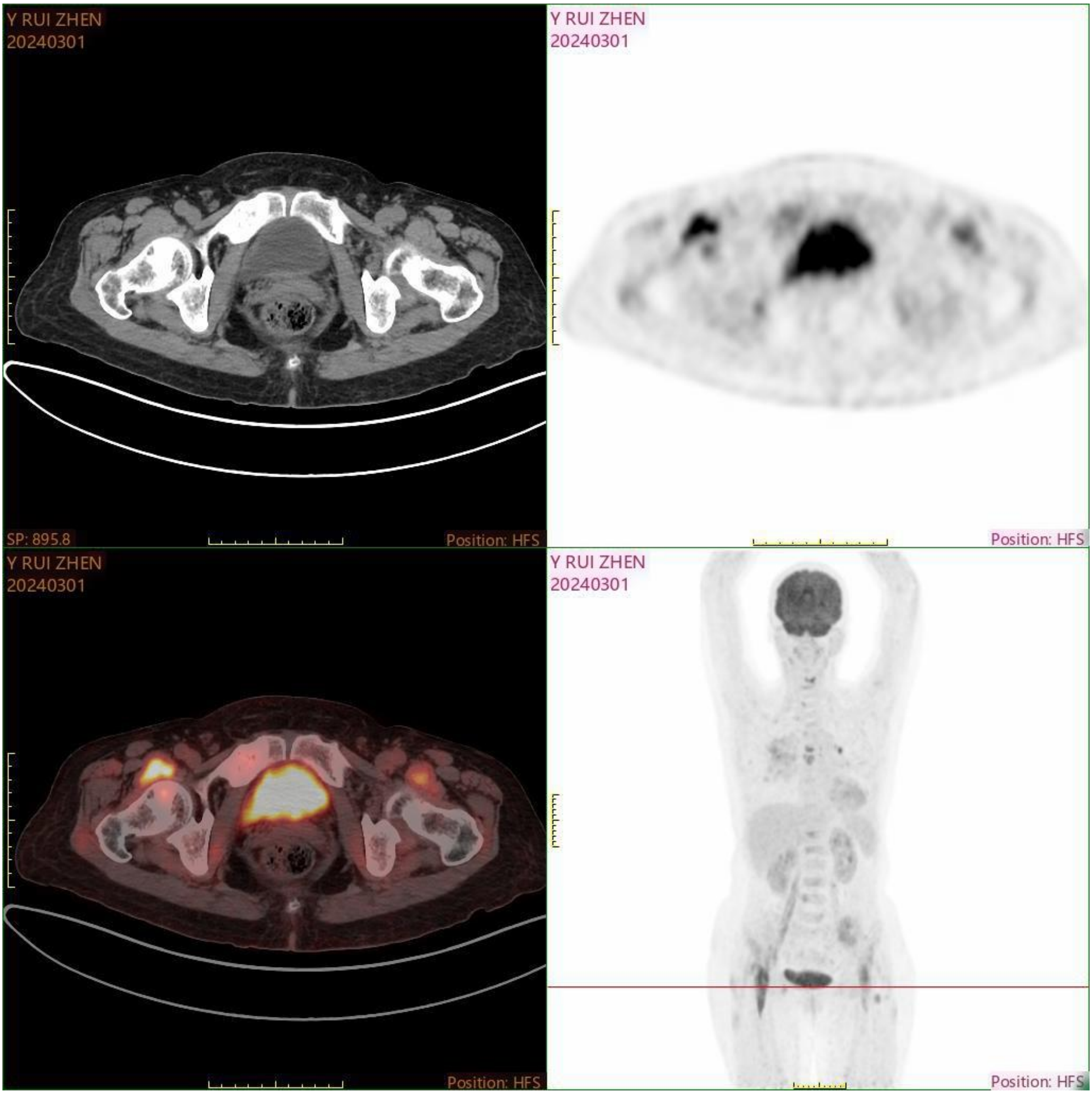

Address:Pavilion 21, No.117 Liuhua Road, Yuexiu
Tel: 020-83220688

PET-CT

Name: YEH **Sex:** **Age: 63** **Inspection date: 2024-** **Inspection No.:**

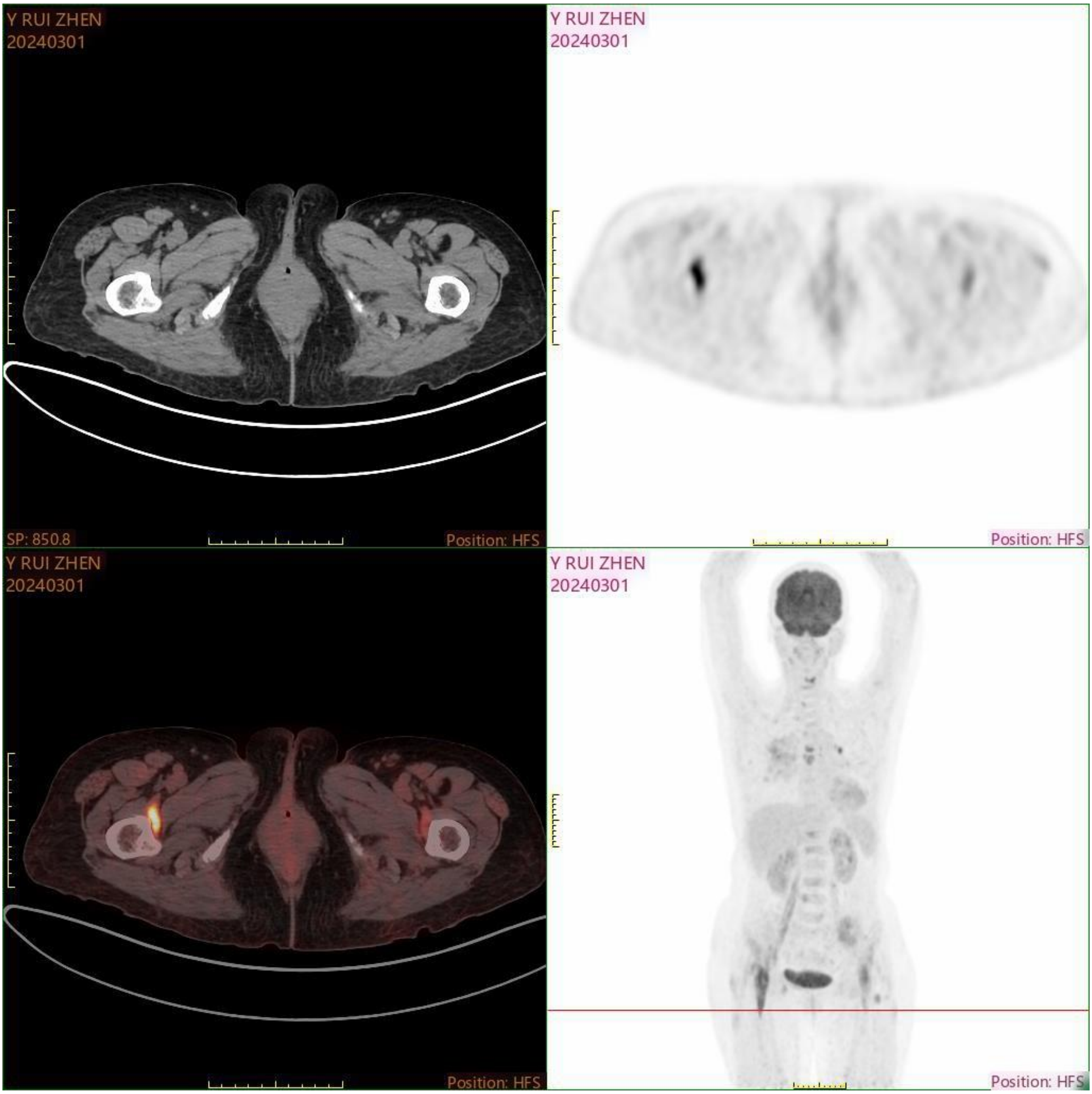

Address:Pavilion 21, No.117 Liuhua Road, Yuexiu Tel: 020-83220688

PET-CT

Name: YEH **Sex:** **Age: 63** **Inspection date: 2024-** **Inspection No.:**

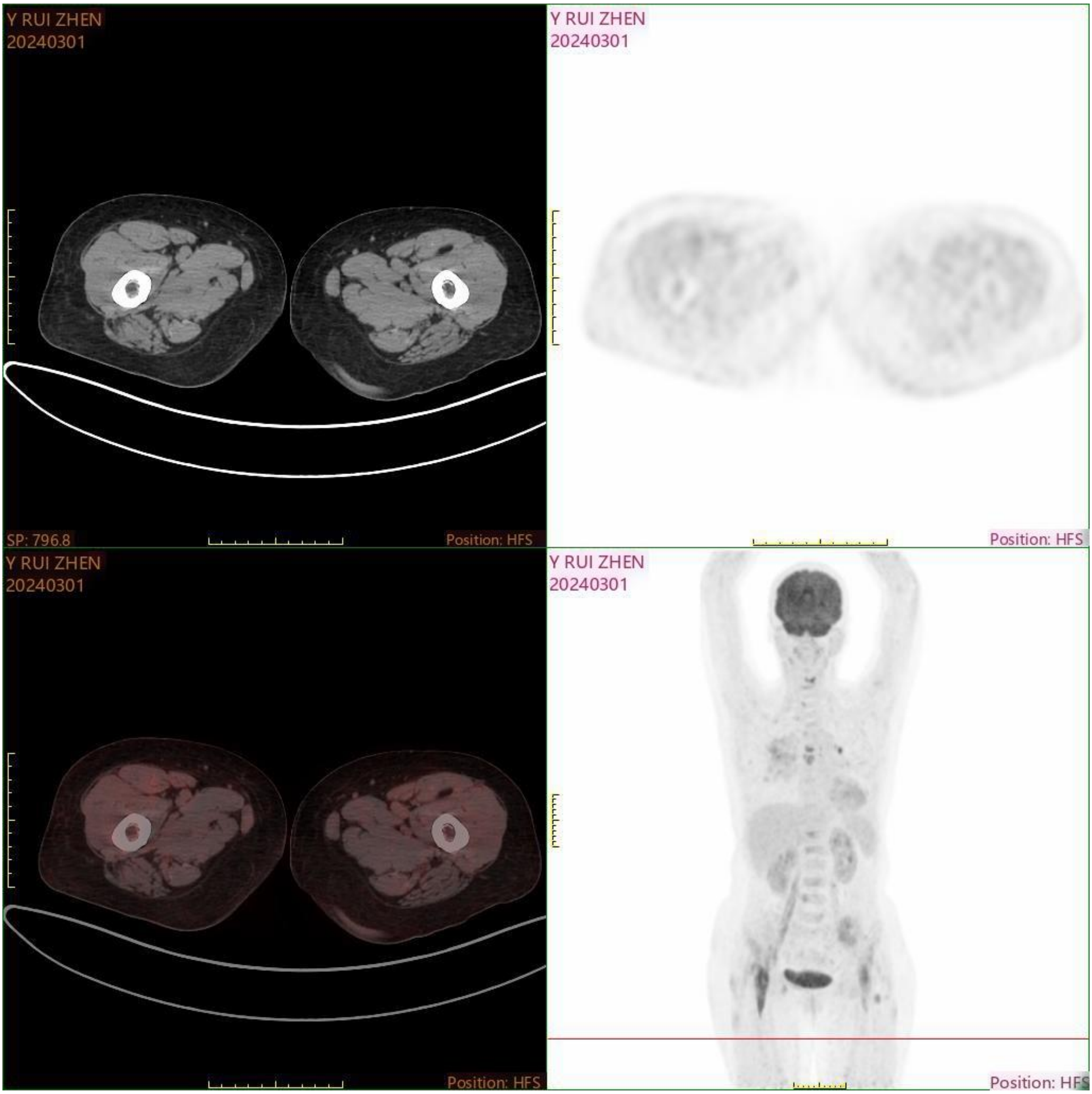

Address:Pavilion 21, No.117 Liuhua Road, Yuexiu
Tel: 020-83220688
